# How Is Scale Incorporated Into the Economic Evaluation of Interventions to Prevent Obesity or to Improve Obesity‐Related Risk Factors: A Systematic Scoping Review

**DOI:** 10.1111/obr.13942

**Published:** 2025-05-21

**Authors:** Carina Dalton, Marufa Sultana, Kaitlyn McKenna, Vicki Brown

**Affiliations:** ^1^ Deakin Health Economics, Institute for Health Transformation Deakin University Geelong Australia

**Keywords:** economic evaluation, health economics, obesity, scale up

## Abstract

**Introduction:**

Obesity prevention interventions commonly need to be implemented at scale, to address what is a significant population‐level issue. While systematic reviews on the economic evidence for interventions preventing obesity or reducing obesity‐related risk factors exist, to date there has been no empirical focus on the methods used to quantify the impacts of scale on intervention cost‐effectiveness. This systematic scoping review aimed to synthesize the methods used to incorporate scale considerations and provide future directions for incorporating scale into economic evaluation of public health interventions.

**Methods:**

A systematic search was undertaken by two reviewers using six databases in June 2023 to identify published economic evaluations of obesity prevention interventions, from which primary studies that quantitatively incorporated scale into their analyses were identified and included. Narrative synthesis of methods used to incorporate scale considerations.

**Results:**

Fifty‐one relevant primary studies were identified, comprising five within‐trial and 46 modeled economic evaluations of 132 discrete interventions. Within‐trial economic evaluations commonly estimated intervention cost assuming scale, and generally used simplistic methods and assumptions to do so. Only three modeled economic evaluations of interventions actually implemented at scale were identified. The methods used to estimate scale impacts on costs, effects, and populations exposed to interventions were heterogeneous, with few studies including equity‐informed analyses.

**Conclusions:**

More guidance is needed on how to appropriately incorporate scale into economic evaluations, whether conducted within‐trial or using modeling approaches. This is especially important due to the necessity of population‐level interventions to address major health issues like obesity.

## 1 Introduction

Obesity is a major public health issue experienced globally [1]. Given the significant health, economic, and social impacts, it is well‐recognized that effective and cost‐effective population‐level interventions delivered at scale are required to moderate or modulate the effects of global obesity drivers [2].

Scale‐up is defined as the “deliberate efforts to increase the impact of successfully tested health innovations to benefit more people and to foster policy and program development on a lasting basis” [3]. Public health interventions that show promise in controlled or research settings must be implemented cost‐effectively at scale, to improve population health and deliver positive returns on investment to funders [4, 5]. Additionally, the costs and impacts of interventions unsuitable for testing using traditional research methods (such as randomized controlled trials (RCTs)) must be understood. Evidence suggests that innovative methods, such as natural experiments, could be more utilized in evaluating such interventions [6].

Traditional health economic evaluation involves the comparative analysis of at least two alternatives in terms of both costs and consequences [7]. Economic evaluations can be conducted alongside RCTs in controlled environments, but these conditions may not necessarily represent real‐world conditions accurately. Alternatively, economic evaluations can also be performed using modeling techniques based on best available evidence, to extrapolate the costs and effects of interventions across a longer time horizon and/or broader decision context or for interventions not conducive to scientific testing using methods such as RCTs [7, 8].

Evidence suggests that intervention effects may change when implemented at scale, as compared to when measured in a controlled research environment, often referred to as the “scale‐up penalty” or “voltage drop” [9, 10]. The costs of interventions at scale may also differ [11], but little evidence exists on how this should be accounted for in economic evaluations. Recommended guidelines for health economic evaluation, such as the Second Panel on Cost‐Effectiveness in Health [12], do not provide specific advice on how health economists should determine and report policy and practice‐relevant information related to implementation and scale‐up. The Consolidated Health Economic Evaluation Reporting Standards (CHEERS) recommend reporting uncertainty and discussions of generalizability [13], but do not provide in‐depth guidance specifically related to the concept of scale and how it may impact cost‐effectiveness findings. In addition, implementation and scale‐up frameworks that are used by implementation scientists to support the translation of research to real‐world settings, such as RE‐AIM [14], vary in their integration of efficiency and cost‐effectiveness considerations into implementation and scale‐up theory and practice [15].

Numerous systematic reviews on the cost‐effectiveness of interventions to prevent obesity or obesity‐related risk factors, such as physical inactivity or unhealthy diets, have been published (e.g., [16, 17, 18, 19, 20]). However, an in‐depth exploration of how the economic evidence incorporates considerations of scale into the analyses has not been undertaken. Given the evidence on “voltage drop” and the potential for uncertainty regarding intervention costs at scale, it is crucial to consider how scale is incorporated into economic evaluations of obesity prevention interventions as it may impact estimates of cost‐effectiveness. This systematic scoping review therefore aims to synthesize the economic evidence of obesity prevention interventions or interventions aiming to improve obesity‐related risk factors delivered at scale; to examine the methods used within the evaluations to reflect scale; and to provide future directions for building rigorous economic evidence of scaled‐up obesity prevention interventions.

## 2 Methods

This scoping review is reported following the Preferred Reporting Items for Systematic Review and Meta‐Analysis extension for scoping reviews (Supporting Information S1: File 1) [21]. The protocol for the scoping review was registered with Open Science Framework in July 2023 (https://osf.io/7df9u/).

A two‐step literature review approach was adopted. First, a scoping search of published systematic reviews was undertaken. Second, primary studies in identified systematic reviews were assessed for final inclusion in our review.

### 2.1 Eligibility Criteria

Systematic reviews meeting the following criteria were included:

- Peer‐reviewed systematic reviews published in English at any time;
- Systematically synthesized the evidence from full economic evaluation studies (i.e., studies comparing costs and consequences of at least two alternatives) that aimed to prevent obesity or to improve obesity‐related risk factors (physical inactivity, sedentary behavior, poor diet, insufficient sleep) in child or adult populations.

Systematic reviews were excluded if they focused on economic evaluations of interventions in specific disease groups (e.g., cancer patients).

Primary studies that met the following criteria, and were identified from the included systematic reviews, were then selected for final inclusion into our scoping review:

- Economic evaluations that incorporate considerations of scale through potential for increased impact so as to benefit more people [3]. Considerations of scale could be hypothetical or actual:
  ○ population‐level interventions, defined as those that focus on social or policy‐level determinants and that aim to change the socio‐cultural and environmental conditions of risk for large populations (i.e., “upstream” interventions) [22].
  ○ interventions where the population of interest is at scale (i.e., national populations, representative populations, whole populations, large populations or population segments) [23];
- Interventions delivered to a greater number of individuals than that delivered to pre‐scale [10];
- Considerations of scale must be quantitative (i.e., incorporated into quantitative analyses through scale considerations of target population, costs, effectiveness and/or cost‐effectiveness in either base case or sensitivity analyses).

Primary economic evaluation studies of treatment interventions, nutrition interventions targeting other outcomes (for instance, salt reduction), gray literature, or partial economic evaluations were excluded. Papers that reported the results of priority‐setting studies were only included if they incorporated results that had not also been reported in single‐study papers. For example, the study by Gortmaker et al. [24] was excluded as all reported economic evaluations had comprehensive stand‐alone papers already included in our review [25, 26, 27, 28].

### 2.2 Information Sources and Search

The following electronic databases were searched from inception to 19 July 2023:

- Academic Complete, Medline Complete, CINAHL Complete, EconLit (EBSCOHost)
- The Cochrane Database of Systematic Reviews

Example search strategies are presented in Table 1. Full search strategies for each source are given in Supporting Information S1: File 2.

**TABLE 1:** Concepts for search strategy, with example search terms.

Concept	Search terms
Systematic review	“systematic review” OR “meta analaysis” OR “meta‐analysis” OR “systematic literature review” OR “meta synthesis” or “meta‐synthese” OR “umbrella review”
Economic evaluation	“cost effectiv*” OR “cost‐effect*) OR “cost benefit*” OR “cost–benefit” OR “cost utili*” OR “cost‐utili*” OR “cost minimi*” OR “cost‐minimi*” OR “cost consequence” OR “cost‐consequence” OR “economic* evaluat*” OR “economic* analys*” OR “health economics” OR “economic* model*”
Obesity OR obesity‐related behaviors	Obes* or overweight OR BMI OR “body mass” OR “weight gain” OR “weight loss” OR adipos* OR diet OR dietary OR dieting OR nutrition OR nutritional OR nutrient* OR eat OR eating OR fruit OR vegetable* OR “energy intake” OR “physical* activ*” OR exercis* OR “sedentary behavio?r*” OR “sedentary lifestyle*” OR “screen time” OR screentime OR “energy balance” OR “screen based” OR sleep

### 2.3 Selection of Sources of Evidence

Hits from the academic database search were imported into Endnote by one author (CD), and duplicates were removed. Systematic reviews were uploaded into Covidence software [29], for screening by two independent authors (CD, MS, VB) following PRISMA guidelines [30]. Reasons for exclusion of systematic reviews were documented at the full text screening stage.

The references of all primary studies in each included systematic review were entered into Microsoft Excel. Studies appearing in more than one systematic review were de‐duplicated. The full texts and reference lists of primary studies were retrieved and screened by two independent authors (CD, VB) for final inclusion into our scoping review. Reasons for exclusion of primary studies were documented at the title and abstract, and full‐text screening stages.

### 2.4 Data Charting Process and Data Items

Data from included primary studies were extracted into a Microsoft Excel template using items based on the CHEERS statement [13]. Extracted information included the publication reference details, country, study design, target population, setting, intervention and comparator, measures of effectiveness, study perspective, time horizon, methods for estimating resource use, model specification (if applicable), discount rate, summary of results, uncertainty and sensitivity analyses and funding source. Data was extracted by one reviewer (CD) and cross‐checked by a second reviewer (VB, KM).

### 2.5 Synthesis of Results

Data were narratively synthesized, focusing on the target population, and methods for estimating effectiveness and resource use at scale. The synthesis was undertaken in accordance with the Synthesis Without Meta‐Analysis (SwiM) guidelines (Supporting Information S1: File 3) [31]. Quality assessment was not undertaken due to the exploratory aim of the review [32].

## 3 Results

**FIGURE 1:**
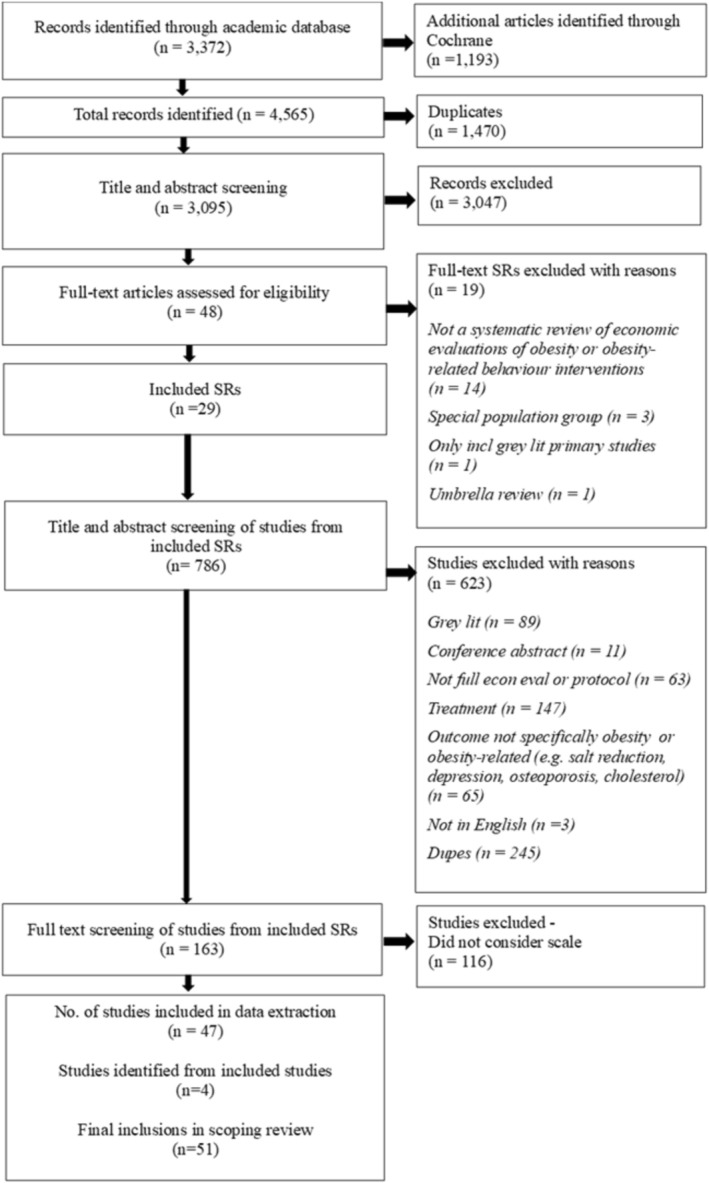
PRISMA flowchart of included studies.

Title and abstract screening of 3095 articles was undertaken, to identify 29 relevant systematic reviews of economic evaluations of obesity or obesity‐related risk factor interventions [16, 17, 18, 19, 20, 33, 34, 35, 36, 37, 38, 39, 40, 41, 42, 43, 44, 45, 46, 47, 48, 49, 50, 51, 52, 53, 54, 55, 56], from which 51 relevant primary studies were included (Figure 1). These comprised five within‐trial economic evaluations [57, 58, 59, 60, 61] and 46 studies that incorporated modeled economic evaluation [25, 26, 27, 28, 62, 63, 64, 65, 66, 67, 68, 69, 70, 71, 72, 73, 74, 75, 76, 77, 78, 79, 80, 81, 82, 83, 84, 85, 86, 87, 88, 89, 90, 91, 92, 93, 94, 95, 96, 97, 98, 99, 100, 101, 102, 103]. Detailed summaries of the included studies in our review, based on the data extraction template, are available in Supporting Information S1: File 4.

### 3.1 Within‐Trial Economic Evaluations Incorporating Scale Into Analyses

Five within‐trial economic evaluations were identified that considered scale in their analyses. Studies were undertaken in Australia (*n* = 3), Sweden (*n* = 1) and Portugal (*n* = 1). Interventions were conducted in maternal child healthcare (*n* = 3) and school (*n* = 2) settings (Appendix 4). Two studies [59, 61] presented cost estimates assuming scale alongside the within‐trial cost‐effectiveness results. Killedar et al. [59] multiplied the within‐trial intervention costs by the number of potential participants should the Communicating Healthy Beginnings Advice by Telephone (CHAT) short‐message‐service or Healthy Beginnings home‐visiting interventions be scaled to delivery to all newborns in New South Wales, Australia. Vieira et al. [61] estimated a lower intervention cost assuming the school‐based intervention was delivered at scale, as compared to costs within the research study, and multiplied costs by the number of children eligible in metropolitan Porto.

Two studies [57, 58] undertook scenario‐based cost‐effectiveness analyses, by reducing the within‐trial estimate of the cost of the intervention for scale‐up. Justification for reduced intervention cost included a halving of meeting time for an early childhood intervention delivered in child health centers to better align with current practices [57], and a reduction in travel time cost for a home‐visiting intervention for infants due to economies of scale (although the authors noted that travel time savings would not necessarily be achievable in rural or remote settings) [58]. The potential for cost‐effectiveness was substantially increased in both cases (Table 2).

**TABLE 2:** Summary of scale considerations in within‐trial economic evaluations.

Study	Summary of study design and how scale was considered
Doring et al. [57]	Within‐trial CEA Scenario analysis, where the cost of the intervention was altered to better reflect implementation in current child healthcare practices. Reduction in time costs for intervention delivery to reflect shorter meetings (50% duration) Effect measure kept constant ICER reduced from EUR3,109 (base case) to EUR2,128 per BMI unit prevented and probability of cost‐effectiveness increased by approximately 20% in this scenario
Hayes et al. [58]	Within‐trial CEA Scenario analysis reflected delivery of program in a “real‐world” setting. Reduction in travel and administration time costs for intervention delivery to reflect shorter travel distances than within‐trial Effect measure kept constant The probability of the intervention being cost‐effective was substantially increased under this scenario (probability of being cost‐effective of 0.3 as delivered in the trial vs. 0.66; acceptability threshold of $500 for a 0.1 BMI z‐score reduction).
Killedar et al. [59]	Within‐trial CEA Intervention cost estimates assuming scale presented alongside the within‐trial cost‐effectiveness results Multiplication of mean intervention cost per child by the number of births in NSW in 2018
Sutherland et al. [60]	Within‐trial CEA Two scenario analyses estimating the cost‐effectiveness of implementing the intervention across secondary schools in NSW (*n* = 254,923 students). Scenario one assumed costs as per the trial and extrapolated to statewide rollout. Scenario two adjusted costs to reflect real world model of delivery (no equipment cost; rather than employment of PA consultant, release time for current staff to train and conduct intervention) Effect size based on the results of the sensitivity analysis conducted within‐trial (imputation of missing data, not adjusted for scale) for both analyses. Cost per additional minute of MVPA $66 (95% CI $35–656) for scenario one; cost per additional minute of MVPA $27 (95% CI $14–267).
Vieira et al. [61]	CCA alongside non‐randomized pre/post study Intervention cost estimate assuming scale, presented alongside CCA. Per capita intervention costs adjusted and multiplied by number of children in elementary schools in metropolitan Porto (*n* = 42,953 eligible children). Per capita intervention cost within study estimated at €36.14; per capita cost assuming scale €18.18.

Abbreviations: CCA, cost‐consequence analysis; CEA, cost‐effectiveness analysis; MVPA, moderate to vigorous physical activity; NSW, New South Wales; PA, physical activity.

Sutherland et al. [60] conducted two sensitivity analyses that estimated the cost‐effectiveness of a multi‐component school‐based physical activity intervention should it be implemented across the state of New South Wales, Australia. The first scenario assumed the implementation model as per the RCT (i.e., employment of a school physical activity consultant). The second scenario acknowledged feasibility challenges with this model at scale given school principal feedback from the trial, and so utilized an existing in‐school teacher for this role.

### 3.2 Modeled Economic Evaluations Incorporating Scale Into Analyses

#### 3.2.1 Overview of Modeled Studies

Forty‐six studies incorporating a modeled economic evaluation of 127 discrete interventions were identified [25, 26, 27, 28, 62, 63, 64, 65, 66, 67, 68, 69, 70, 71, 72, 73, 74, 75, 76, 77, 78, 79, 80, 81, 82, 83, 84, 85, 86, 87, 88, 89, 90, 91, 92, 93, 94, 95, 96, 97, 98, 99, 100, 101, 102, 103]. Studies were conducted in Australia (*n* = 22, 48%), the United States (*n* = 12, 26%), the Netherlands (*n* = 3, 7%), the UK and New Zealand (*n* = 2 respectively, 4%), and France, Mexico, Italy, and Canada (*n* = 1 respectively, 2%). One study was conducted across a number of countries, including Brazil, China, India, and Russia [73]. Ninety‐nine interventions were evaluated using cost‐utility analysis (CUA) [25, 62, 63, 65, 67, 68, 69, 70, 71, 72, 73, 74, 75, 76, 78, 79, 81, 82, 83, 84, 85, 88, 89, 90, 92, 93, 94, 95, 96, 97, 98, 99, 100, 101, 102], 21 interventions were evaluated using cost‐effectiveness analysis (CEA) [27, 28, 66, 77, 86, 87], three interventions were evaluated using both CUA and return‐on‐investment (ROI) [80], three interventions were evaluated using both CEA and CUA [26, 91, 103], and one intervention was evaluated using cost–benefit analysis (CBA) [64].

#### 3.2.2 Studies of Interventions Implemented in the Real World

The majority of economic evaluations were for interventions hypothetically implemented at scale, with only three studies evaluating interventions that had actually been implemented at scale in the real world [67, 81, 92]. Basto‐Abreu et al. [67] undertook a modeled CUA of a sugar‐sweetened beverage (SSB) tax implemented in Mexico. The impact of the tax was estimated based on the annual average decrease in SSB purchases 2 years after implementation, and purchases were assumed to be a close proxy to consumption [67, 104]. Changes in SSB consumption were translated to changes in BMI based on evidence from the literature [105, 106]. Costs were estimated as a fraction of the Ministry of Finance's budget [67]. Frew et al. [81] undertook a modeled CUA of a physical activity program rolled out across the city of Birmingham. A pragmatic approach to estimating the effect of the intervention was taken, as the evaluation started after rollout and so the study used before and after measurements of physical activity levels using self‐reported data. Intervention cost was determined by dividing the total cost of setting up and running the scheme by the number of program members, to obtain average per‐participant annual costs [81]. Mantilla‐Herrera et al. [92] used a difference‐in‐difference analysis of data from pre‐ and post‐implementation of the Health Star Rating (HSR) System in Australia on energy density of products to model potential costs and benefits of implementation on a voluntary (actual) and mandatory (hypothetical) basis. Baseline analysis assumed that changes in energy density of products was 100% attributable to the HSR system, but this was varied in sensitivity analysis. A published government‐commissioned CBA was used in estimating intervention costs.

#### 3.2.3 Estimating Intervention Effectiveness at Scale

In estimating intervention effectiveness at scale, many included studies in our review extrapolated intervention effects from pilot studies (for instance [63, 72, 97, 103] [Supporting Information S1: File 4]). Some studies took a modeled or pathway approach to estimating intervention effect (including those studies using elasticities to estimate potential impacts if an intervention was scaled, for instance [68, 70, 76, 79]). Studies that incorporated estimates of effectiveness derived from meta‐analysis of studies in the literature included the CEA of a hypothetical active physical education policy in schools [27], or the CUA of hypothetical restrictions to television advertising of unhealthy foods [71]. Few studies attempted to adjust effect estimates from experimental studies in explicit recognition of the potential “voltage drop” effect. One study applied a crude adjustment factor, assuming that 50% of effect estimates reported in experimental studies would translate and be maintained in a real‐world setting [71]. Some studies conducted threshold analyses for effect, to estimate the intensity and/or duration of effect required for the intervention to be cost effective. For example, Ananthapavan et al. [62] assessed the duration of effect required for a hypothetical community‐based intervention to be cost‐effective. Brown et al. [71] estimated the minimum effect size required for the intervention to be cost effective using a AUD50,000 per HALY threshold (Supporting Information S1: File 4).

Estimating combined effects of interventions at scale was considered a challenge in studies that attempted to do so, and methods used were heterogeneous. For example, Bemelmans et al. [69] assumed that effects for those who were exposed to both a community and intensive intervention were additive. In the study by Cecchini et al. [73], a combined strategy (mass media campaign, fiscal measures, food advertising regulation, and food labelling) was estimated assuming the effects of the individual interventions would combine multiplicatively.

#### 3.2.4 Estimating Intervention Costs at Scale

Methods to estimate intervention costs at scale were also heterogeneous, and included pathway analysis, estimates from trial records and the literature (Appendix 4). Few studies incorporated threshold analyses of costs, to assess the cost at which an intervention could be delivered at scale and be considered cost‐effective. For instance, Ekwaru et al. [80] expressed intervention cost as that below which the school‐based health promotion program would be cost‐effective at a CA$50,000 threshold level. Several studies engaged with stakeholders to the intervention if delivered at scale, in order to verify cost estimates. For instance, Long et al. [25] engaged in personal correspondence with a contact from the State Department of Revenue in intervention costing of a SSB excise tax hypothetically implemented nationally.

Availability of real‐world evidence on intervention costs was cited as a limitation in several studies (Supporting Information S1: File 4). For example, Robinson et al. [99] estimated the cost‐effectiveness of alcohol price interventions and used parallel evidence for intervention cost components from government reports of similar interventions for costing. The lack of availability of evidence for downstream impacts or indirect costs of intervention was also cited as challenging. In many instances, such costs were omitted from cost estimates of interventions delivered at scale, although these may have had material impacts on overall cost‐effectiveness results. For example, Huse et al. [85] only included costs to government of mandatory restrictions of price promotions on SSBs in Australia. Due to the lack of available data, it was not possible to accurately estimate the financial impact of the modeled policy on retailers or SSB manufacturers. Potential profit changes to industries impacted by policy‐level interventions were often not able to be incorporated into analyses, although these are relevant from a societal perspective (for instance [70, 71, 99]).

#### 3.2.5 Populations Exposed at Scale

Methods for estimating the populations exposed to interventions at scale were also heterogeneous. Some studies were able to make plausible assumptions based on likely reach, for example for school‐based interventions [27, 64, 86]. One study consulted an advisory group for advice on the plausible reach of five interventions to limit television viewing, and reviewed the gray literature on program participation [87]. Several studies included in our review cited the estimation of potential intervention reach as a limitation. For example, Over et al. (98) assumed that the percentage of general practitioners participating in a pedometer intervention would be equal to that offering a similar intervention currently in place, but noted the need for assumptions re reach and uptake of the intervention due to the lack of available evidence.

#### 3.2.6 Equity Considerations at Scale

Relatively few studies included in our review included equity‐informed analyses, to estimate differential costs and/or effects on different population groups of interventions implemented at scale [71, 88, 90]. For example, the study by Lal et al. [88] undertook an evaluation of a SSB tax hypothetically implemented in Australia, and incorporated analyses by socioeconomic (SEP) quintile. Price elasticities for low, middle and high income households (quintile 1, quintile 3, quintile 5) were sourced from the literature, with estimates for quintiles 2 and 4 interpolated. Data on SSB intake by SEP quintile were also used to estimate differential impacts of the hypothetical intervention [88].

#### 3.2.7 Acknowledging Limitations at Scale

Many of the studies included in our review acknowledged that assumptions had been made to reflect scale, which may or may not be realistic or accurate reflections. For example, Kenney et al. [86] included a limitation section on the fact that true implementation costs, cost savings and impact of a water consumption intervention are unknown given that analyses were hypothetical and that effect estimates from studies may not be generalizable, particularly if sociodemographic characteristics exist that may have an influence on cost or effect. Other studies explored some of these issues within sensitivity analyses (e.g., Magnus et al. [91]), but the variation of uncertain parameters related to scale to estimate impacts on overall cost‐effectiveness was not consistent across all included studies in our review.

## 4 Discussion

### 4.1 Summary of Main Results and Interpretation

Our review identified 51 studies, reporting the economic evaluation of 132 discrete interventions, that incorporated scale considerations quantitatively into analyses. A limited number of within‐trial economic evaluations of obesity prevention interventions have quantitatively incorporated scale considerations into their analyses, with relatively simplistic methods. Economic and resource‐related factors are well‐recognized as significant barriers to successful implementation and scale‐up [107]. More rigorous economic evidence on the potential impacts of scale‐up for interventions tested within research settings is clearly required. This could help to better inform effective and cost‐effective resource allocation decision‐making as research progresses through the research translation phases from testing to dissemination, as well as better support implementation [11, 108]. Our findings also demonstrate the need for more evaluations of programs and policies that have actually been implemented at scale, with only three modeled economic evaluations of interventions actually implemented at scale identified. Clearly there is a significant need for better incorporation of evaluation (including economic evaluation) of the policies and programs that are actually invested in, and implemented, in the real world and at scale. This evidence would be useful to ensure the most efficient use of resources when taking into account contextual and other factors, and to support decisions on where further investment (or disinvestment) may be warranted. A recent review found that scale‐up of obesity prevention interventions often occurs in absence of prior evidence of effectiveness, and can be in response to a serendipitous alignment of health priorities, political opportunity and supportive policy and contextual factors [5]. The review noted however that scale‐up of untested interventions could have unintended consequences, be ineffective and a poor use of scarce health resources that could have best been applied elsewhere [5].

**FIGURE 2:**
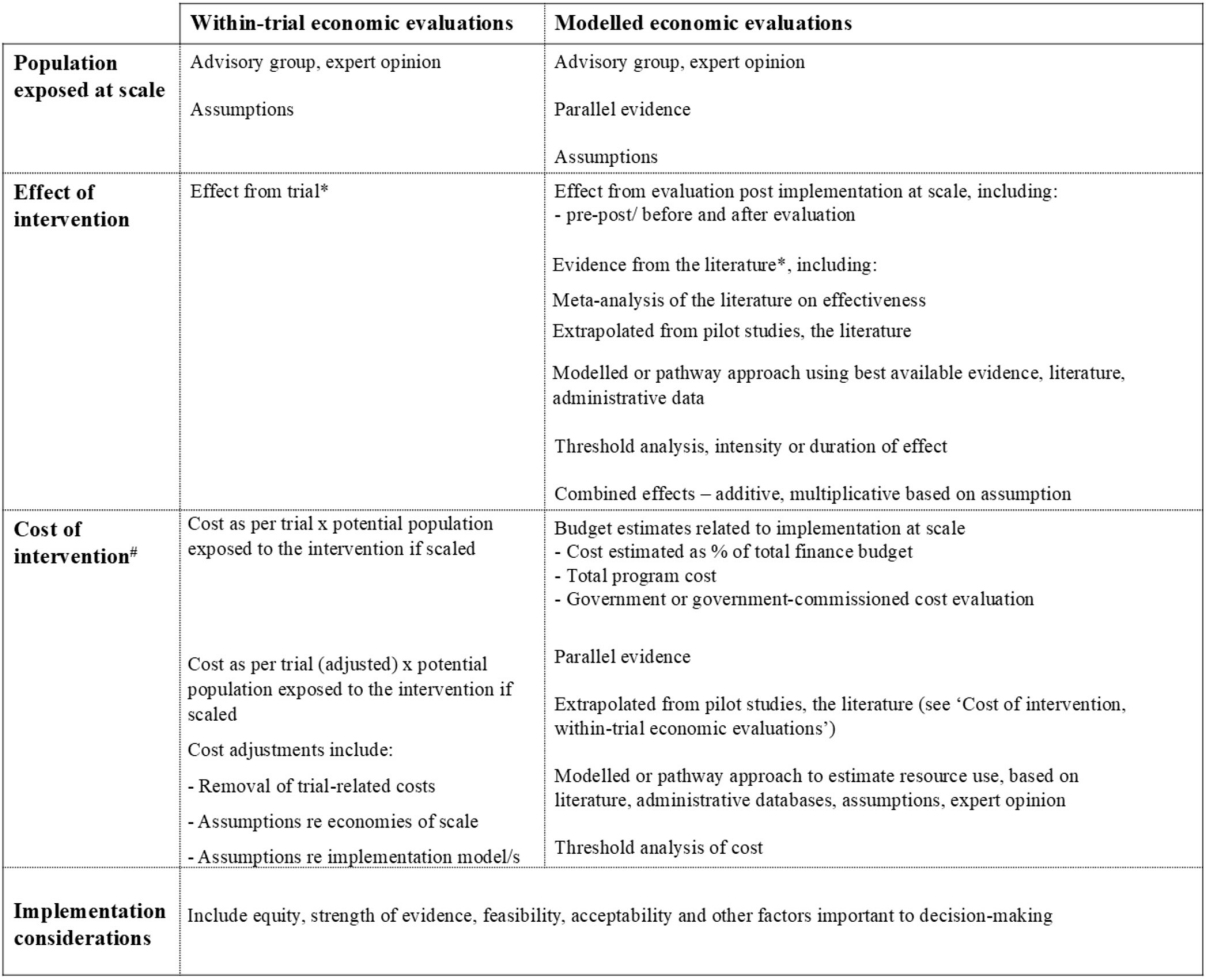
Preliminary typology of approaches to incorporate scale in within‐trial and modeled economic evaluations identified from studies included in the review.

Our review identified heterogeneous methods for estimating the effects of interventions delivered at scale (Figure 2), and that this may be impacting on overall estimates of cost‐effectiveness. Studies included in our review differed in terms of their sources for evidence of effectiveness, from single estimates from pilot or research trials, to estimates derived from logic pathways and available data, to estimates of effect from meta‐analysis. A recent priority‐setting study of obesity prevention interventions conducted in Australia developed an evidence framework to support explicit and transparent evidence utilization in decision‐making [109]. Criteria for determining the certainty of effect used in the economic evaluations provided decision‐makers with assessments of the strength of evidence based on the rigor of study design and the outcome measured [109]; however, more explicit incorporation of potential scale impacts such as “voltage drop” into this and similar frameworks warrants consideration. Very few studies included in our review incorporated the concept of “voltage drop” into their estimates of effect and this should be further explored in future given evidence of its existence [9, 10]. Presenting implementation considerations, factors that may influence decision‐making but that may not be easily quantified and incorporated into economic evaluation, also presents a positive step in terms of conveying important information related to scale. Such summaries have been incorporated into several studies conducted in Australia and the United States (for instance [27, 65]) but could be tailored to more specifically incorporate potential scale uncertainties and impacts on overall cost‐effectiveness results and routinely presented alongside results from economic evaluations.

Obesity prevention interventions are complex and a diverse set of options for intervention exist across all levels of the socio‐ecological model [110]. Studies included in our review that had attempted to evaluate a package of interventions cited the inherent challenge in doing so (e.g., [69, 73]). Methodological guidance on estimating the combined effects of interventions delivered simultaneously, or how to best attribute costs and effects to specific interventions, is lacking and this is also an important area for future work [111].

Estimating the cost of interventions delivered at scale is challenging given the influence of context on resources and resource use. Several studies included in our review incorporated threshold analyses for CUA results, reporting the costs at which an intervention could be delivered up to for it to be considered cost‐effective given commonly accepted CUA thresholds (e.g., [80]). The incorporation of such analyses allows for not only the provision of cost‐effectiveness information based on best available evidence, but also a cost range for decision‐makers to consider within their own contexts. This practice should be more regularly included into economic evaluations of interventions implemented at scale. The incorporation of qualitative methods and stakeholder engagement to help inform economic evaluations of scaled interventions is also a positive step in the generation of economic evidence that is policy and practice relevant, and should be incorporated wherever feasible. This is particularly important for the accuracy and relevancy of cost‐effectiveness of interventions that may have commercial or other impacts, and where limited publicly‐available data on these impacts may be available. Regardless, real‐world data on costs may continue to remain difficult to capture, especially when considering downstream impacts such as changes in revenue of commercial entities impacted by an intervention.

### 4.2 Implications for Practice

Comprehensive methodological guidance on incorporating scale considerations into economic evaluation does not currently exist. The development of such guidance could better inform health economists on how to factor impacts of scale into their analyses, and increase transparency of the methods commonly used to do so. Transparency in the methods used to account for many of the uncertainties inherent in the economic evaluation of scaled interventions is critical, and much broader scope for the inclusion of more detail on the data and assumptions used to reflect scale in economic evaluations of obesity prevention interventions exists. This work forms part of a broader research agenda, focused on the development of methodological guidance for health economists to consider when undertaking economic evaluations of public health interventions implemented at scale. The findings from this review have informed a preliminary typology of approaches to incorporating scale in economic evaluations of obesity prevention interventions (Figure 2). A key component of this future work will be to further develop and refine this typology of methods that could be used for considering scale in economic evaluations of public health interventions more broadly, and the development of a consensus approach to rigorous, practical, and pragmatic guidance for health economists working in this area. A comprehensive understanding of the ways in which scale is currently incorporated into the economic evaluation of obesity prevention interventions forms a first and important step towards this goal. Evidence of cost‐effectiveness of interventions delivered to diverse population groups at scale is also a significant and well‐recognized area for future work, using equity‐informed methods such as distributional cost‐effectiveness analyses [112].

### 4.3 Strengths and Limitations

Study strengths of our scoping review included leveraging the systematic reviews of the economic evidence for obesity prevention interventions that have already been published to streamline the search for relevant papers, and the systematic screening, data extraction and synthesis by multiple reviewers. A limitation is that in summarizing a large and disparate body of literature using diverse methods to incorporate scale into analyses, and at times where details of such methods were limited in academic publications, we may have omitted scale‐related considerations. In addition, our review focused on scale considerations in the academic literature, and economic evaluations published in the gray literature were not considered. It is possible that additional economic evaluations of interventions actually implemented at scale will have been published in the gray literature but are not included in our synthesis given the academic literature focus.

## 5 Conclusions

To address the population‐level burden of obesity, large‐scale implementation of prevention interventions is required. Findings from our review demonstrate that significant evidence for the cost‐effectiveness of obesity prevention interventions at scale exists. However, methods used to incorporate the impacts of scale on costs and effects, and the populations reached by interventions, is heterogenous. More guidance to health economists on how scale should be appropriately incorporated into economic evaluations is required and is a significant area for future work. This could lead to the more rigorous provision of economic evidence that is relevant to real‐world resource allocation decisions, and could reduce the economic‐related barriers to successful and sustainable delivery of public health interventions to populations.

## Author Contributions

V.B. and C.D. conceptualized the study. Search strategies and data extraction were designed and conducted by C.D., K.M., and M.S. Analysis was undertaken by C.D. and V.B., with input from all authors. The first draft of the manuscript was written by C.D. and V.B., and all authors comments on versions of the manuscript. All authors read and approved the final manuscript.

## Ethics Statement

All data utilized in this manuscript were secondary data (review article), and so ethics approval was not required.

## Conflicts of Interest

The authors declare no conflicts of interest.

## Supporting information


**Data S1** Preferred Reporting Items for Systematic Review and Meta‐Analysis extension for scoping reviews (PRISMA‐ScR) (1).
**Data S2** Search strategies, by source.
**Data S3** Synthesis Without Meta‐Analysis guidelines (2).
**Data S4** Characteristics of studies included in the review.

## Data Availability

Data and materials are available on request to the corresponding author.

## References

[1] N. H. Phelps , R. K. Singleton , B. Zhou , et al., “Worldwide Trends in Underweight and Obesity from 1990 to 2022: A Pooled Analysis of 3663 Population‐Representative Studies with 222 Million Children, Adolescents, and Adults,” Lancet 403, no. 10431 (2024): 1027–1050, 10.1016/s0140-6736(23)02750-2. 38432237 PMC7615769

[2] B. A. Swinburn , G. Sacks , K. D. Hall , et al., “The Global Obesity Pandemic: Shaped by Global Drivers and Local Environments,” Lancet 378, no. 9793 (2011): 804–814. 21872749 10.1016/S0140-6736(11)60813-1

[3] World Health Organization , Nine Steps for Developing a Scaling‐Up Strategy (WHO, 2010).

[4] A. Milat , K. Lee , K. Conte , et al., “Intervention Scalability Assessment Tool: A Decision Support Tool for Health Policy Makers and Implementers,” Health Research Policy and Systems 18, no. 1 (2020): 1. 31900230 10.1186/s12961-019-0494-2 PMC6942323

[5] K. Lee , A. Bauman , L. Wolfenden , P. Phongsavan , and M. Crane , “How Long Does It Take to Scale‐Up Obesity Prevention Interventions?” Preventive Medicine 185 (2024): 108012. 38821419 10.1016/j.ypmed.2024.108012

[6] M. Crane , E. Bohn‐Goldbaum , A. Grunseit , and A. Bauman , “Using Natural Experiments to Improve Public Health Evidence: A Review of Context and Utility for Obesity Prevention,” Health Research Policy and Systems 18, no. 1 (2020): 48. 32423438 10.1186/s12961-020-00564-2 PMC7236508

[7] M. Drummond , M. Sculpher , C. Claxton , G. Stoddart , and G. Torrance , Methods for the Economic Evaluation of Health Care Programmes (Oxford University Press, 2015).

[8] H. Weatherly , M. Drummond , K. Claxton , et al., “Methods for Assessing the Cost‐Effectiveness of Public Health Interventions: Key Challenges and Recommendations,” Health Policy 93, no. 2 (2009): 85–92, 10.1016/j.healthpol.2009.07.012. 19709773

[9] S. McCrabb , C. Lane , A. Hall , et al., “Scaling‐Up Evidence‐Based Obesity Interventions: A Systematic Review Assessing Intervention Adaptations and Effectiveness and Quantifying the Scale‐Up Penalty,” Obesity Reviews 20, no. 7 (2019): 964–982. 30868745 10.1111/obr.12845

[10] C. Lane , S. McCrabb , N. Nathan , et al., “How Effective Are Physical Activity Interventions When They Are Scaled‐Up: A Systematic Review,” International Journal of Behavioral Nutrition and Physical Activity 18, no. 1 (2021): 16. 33482837 10.1186/s12966-021-01080-4 PMC7821550

[11] V. Brown , H. Tran , J. Williams , R. Laws , and M. Moodie , “Exploring the Economics of Public Health Intervention Scale‐Up: A Case Study of the Supporting Healthy Image, Nutrition and Exercise (SHINE) Cluster Randomised Controlled Trial,” BMC Public Health 22, no. 1 (2022): 1338. 35836222 10.1186/s12889-022-13754-0 PMC9281014

[12] G. D. Sanders , P. J. Neumann , A. Basu , et al., “Recommendations for Conduct, Methodological Practices, and Reporting of Cost‐Effectiveness Analyses: Second Panel on Cost‐Effectiveness in Health and Medicine,” Journal of the American Medical Association 316, no. 10 (2016): 1093–1103, 10.1001/jama.2016.12195. 27623463

[13] D. Husereau , M. Drummond , F. Augustovski , et al., “Consolidated Health Economic Evaluation Reporting Standards 2022 (CHEERS 2022) Statement: Updated Reporting Guidance for Health Economic Evaluations,” BMC Medicine 20, no. 1 (2022): 23. 35022047 10.1186/s12916-021-02204-0 PMC8753858

[14] R. E. Glasgow , S. M. Harden , B. Gaglio , et al., “RE‐AIM Planning and Evaluation Framework: Adapting to New Science and Practice With a 20‐Year Review,” Frontiers in Public Health 7 (2019): 64. 30984733 10.3389/fpubh.2019.00064 PMC6450067

[15] V. Brown , H. Tran , M. Blake , R. Laws , and M. Moodie , “A Narrative Review of Economic Constructs in Commonly Used Implementation and Scale‐Up Theories, Frameworks and Models,” Health Research Policy and Systems 18, no. 1 (2020): 124. 33115502 10.1186/s12961-020-00649-y PMC7594311

[16] O. Onyimadu , M. Violato , N. M. Astbury , et al., “A Systematic Review of Economic Evaluations of Interventions Targeting Childhood Overweight and Obesity,” Obesity Reviews 24 (2023): e13597. 37463862 10.1111/obr.13597

[17] V. Brown , H. Tran , K. L. Downing , K. D. Hesketh , and M. Moodie , “A Systematic Review of Economic Evaluations of Web‐Based or Telephone‐Delivered Interventions for Preventing Overweight and Obesity and/or Improving Obesity‐Related Behaviors,” Obesity Reviews 22, no. 7 (2021): e13227. 33763956 10.1111/obr.13227

[18] S. Mahdi , C. Marr , N. J. Buckland , and J. Chilcott , “Methods for the Economic Evaluation of Obesity Prevention Dietary Interventions in Children: A Systematic Review and Critical Appraisal of the Evidence,” Obesity Reviews 23, no. 9 (2022): e13457. 35478373 10.1111/obr.13457 PMC9542346

[19] M. Sultana , M. Nichols , M. Moodie , S. Allender , and V. Brown , “A Systematic Review of Economic Evidence for Community‐Based Obesity Prevention Interventions in Children,” Obesity Reviews 24, no. 9 (2023): e13592. 37308321 10.1111/obr.13592 PMC10909472

[20] M. Zanganeh , P. Adab , B. Li , and E. Frew , “A Systematic Review of Methods, Study Quality, and Results of Economic Evaluation for Childhood and Adolescent Obesity Intervention,” International Journal of Environmental Research and Public Health 16, no. 3 (2019): 485. 30743995 10.3390/ijerph16030485 PMC6388206

[21] A. C. Tricco , E. Lillie , W. Zarin , et al., “PRISMA Extension for Scoping Reviews (PRISMA‐ScR): Checklist and Explanation,” Annals of Internal Medicine 169, no. 7 (2018): 467–473. 30178033 10.7326/M18-0850

[22] K. L. Frohlich , “Commentary: What Is a Population‐Based Intervention? Returning to Geoffrey Rose,” International Journal of Epidemiology 43, no. 4 (2014): 1292–1293. 24844844 10.1093/ije/dyu111 PMC4258785

[23] A. J. Milat , L. King , R. Newson , et al., “Increasing the Scale and Adoption of Population Health Interventions: Experiences and Perspectives of Policy Makers, Practitioners, and Researchers,” Health Research Policy and Systems 12 (2014): 18. 24735455 10.1186/1478-4505-12-18 PMC3996855

[24] S. L. Gortmaker , M. W. Long , S. C. Resch , et al., “Cost Effectiveness of Childhood Obesity Interventions: Evidence and Methods for CHOICES,” American Journal of Preventive Medicine 49, no. 1 (2015): 102–111. 26094231 10.1016/j.amepre.2015.03.032 PMC9508900

[25] M. W. Long , S. L. Gortmaker , Z. J. Ward , et al., “Cost Effectiveness of a Sugar‐Sweetened Beverage Excise Tax in the U.S,” American Journal of Preventive Medicine 49, no. 1 (2015): 112–123. 26094232 10.1016/j.amepre.2015.03.004 PMC8969866

[26] K. R. Sonneville , M. W. Long , Z. J. Ward , et al., “BMI and Healthcare Cost Impact of Eliminating Tax Subsidy for Advertising Unhealthy Food to Youth,” American Journal of Preventive Medicine 49, no. 1 (2015): 124–134. 26094233 10.1016/j.amepre.2015.02.026

[27] J. L. Barrett , S. L. Gortmaker , M. W. Long , et al., “Cost Effectiveness of an Elementary School Active Physical Education Policy,” American Journal of Preventive Medicine 49, no. 1 (2015): 148–159. 26094235 10.1016/j.amepre.2015.02.005

[28] D. R. Wright , E. L. Kenney , C. M. Giles , et al., “Modeling the Cost Effectiveness of Child Care Policy Changes in the U.S,” American Journal of Preventive Medicine 49, no. 1 (2015): 135–147. 26094234 10.1016/j.amepre.2015.03.016

[29] Veritas Health Innovation . Covidence Systematic Review Software Melbourne, Australia 2024, www.covidence.org.

[30] M. J. Page , J. E. McKenzie , P. M. Bossuyt , et al., “The PRISMA 2020 Statement: An Updated Guideline for Reporting Systematic Reviews,” BMJ 372 (2021): n71, 10.1136/bmj.n71. 33782057 PMC8005924

[31] C. Mhairi , E. M. Joanne , S. Amanda , et al., “Synthesis Without meta‐Analysis (SWiM) in Systematic Reviews: Reporting Guideline,” BMJ 368 (2020): l6890. 31948937 10.1136/bmj.l6890 PMC7190266

[32] M. D. J. Peters , C. Marnie , A. C. Tricco , et al., “Updated Methodological Guidance for the Conduct of Scoping Reviews,” JBI Evidence Synthesis 18, no. 10 (2020): 2119–2126. 33038124 10.11124/JBIES-20-00167

[33] V. Brown , B. Z. Diomedi , M. Moodie , J. L. Veerman , and R. Carter , “A Systematic Review of Economic Analyses of Active Transport Interventions That Include Physical Activity Benefits,” Transport Policy 45 (2016): 190–208.

[34] G. Fattore , F. Ferrè , M. Meregaglia , E. Fattore , and C. Agostoni , “Critical Review of Economic Evaluation Studies of Interventions Promoting low‐Fat Diets,” Nutrition Reviews 72, no. 11 (2014): 691–706. 25323698 10.1111/nure.12142

[35] F. Müller‐Riemenschneider , T. Reinhold , and S. N. Willich , “Cost‐Effectiveness of Interventions Promoting Physical Activity,” British Journal of Sports Medicine 43, no. 1 (2009): 70–76. 18971249 10.1136/bjsm.2008.053728

[36] M. M. J. Galekop , C. A. Uyl‐de Groot , and R. W. Ken , “A Systematic Review of Cost‐Effectiveness Studies of Interventions With a Personalized Nutrition Component in Adults,” Value in Health 24, no. 3 (2021): 325–335. 33641765 10.1016/j.jval.2020.12.006

[37] S. Garrett , C. R. Elley , S. B. Rose , D. O'Dea , B. A. Lawton , and A. C. Dowell , “Are Physical Activity Interventions in Primary Care and the Community Cost‐Effective? A Systematic Review of the Evidence,” British Journal of General Practice 61, no. 584 (2011): e125–e133. 10.3399/bjgp11X561249 PMC3047345 21375895

[38] V. Gc , E. C. Wilson , M. Suhrcke , W. Hardeman , and S. Sutton , “Are Brief Interventions to Increase Physical Activity Cost‐Effective? A Systematic Review,” British Journal of Sports Medicine 50, no. 7 (2016): 408–417, 10.1136/bjsports-2015-094655. 26438429 PMC4819643

[39] M. Gebreslassie , F. Sampaio , C. Nystrand , R. Ssegonja , and I. Feldman , “Economic Evaluations of Public Health Interventions for Physical Activity and Diet: Systematic Review,” European Journal of Public Health 30, no. Supplement_5 (2020): ckaa165.1197. 10.1016/j.ypmed.2020.106100 32353572

[40] M. Guarino , L. Matonti , F. Chiarelli , and A. Blasetti , “Primary Prevention Programs for Childhood Obesity: Are They Cost‐Effective?” Italian Journal of Pediatrics 49, no. 1 (2023): 28. 36864472 10.1186/s13052-023-01424-9 PMC9983264

[41] R. K. Hodder , K. M. O'Brien , S. Lorien , et al., “Interventions to Prevent Obesity in School‐Aged Children 6‐18 Years: An Update of a Cochrane Systematic Review and meta‐Analysis Including Studies From 2015‐2021,” EClinicalMedicine. 54 (2022): 101635. 36281235 10.1016/j.eclinm.2022.101635 PMC9581512

[42] V. Jacob , S. K. Chattopadhyay , J. A. Reynolds , et al., “Economics of Interventions to Increase Active Travel to School: A Community Guide Systematic Review,” American Journal of Preventive Medicine 60, no. 1 (2021): e27–e40. 33341185 10.1016/j.amepre.2020.08.002 PMC7770808

[43] K. Korber , “Quality Assessment of Economic Evaluations of Health Promotion Programs for Children and Adolescents—A Systematic Review Using the Example of Physical Activity,” Health Economics Review 5, no. 1 (2015): 35. 26603159 10.1186/s13561-015-0071-5 PMC4658341

[44] J. Laine , V. Kuvaja‐Köllner , E. Pietilä , M. Koivuneva , H. Valtonen , and E. Kankaanpää , “Cost‐Effectiveness of Population‐Level Physical Activity Interventions: A Systematic Review,” American Journal of Health Promotion 29, no. 2 (2014): 71–80. 25361461 10.4278/ajhp.131210-LIT-622

[45] T. Lehnert , D. Sonntag , A. Konnopka , S. Riedel‐Heller , and H. H. König , “The Long‐Term Cost‐Effectiveness of Obesity Prevention Interventions: Systematic Literature Review,” Obesity Reviews 13, no. 6 (2012): 537–553. 22251231 10.1111/j.1467-789X.2011.00980.x

[46] N. Lutz , P. Clarys , I. Koenig , T. Deliens , J. Taeymans , and N. Verhaeghe , “Health Economic Evaluations of Interventions to Increase Physical Activity and Decrease Sedentary Behavior at the Workplace: A Systematic Review,” Scandinavian Journal of Work, Environment & Health 46, no. 2 (2020): 127–142. 10.5271/sjweh.3871 31820003

[47] R. A. McKinnon , S. M. Siddiqi , F. J. Chaloupka , L. Mancino , and K. Prasad , “Obesity‐Related Policy/Environmental Interventions: A Systematic Review of Economic Analyses,” American Journal of Preventive Medicine 50, no. 4 (2016): 543–549, 10.1016/j.amepre.2015.10.021. 26707464

[48] P. Nguyen , L. K. Le , J. Ananthapavan , L. Gao , D. W. Dunstan , and M. Moodie , “Economics of Sedentary Behaviour: A Systematic Review of Cost of Illness, Cost‐Effectiveness, and Return on Investment Studies,” Preventive Medicine 156 (2022): 106964. 35085596 10.1016/j.ypmed.2022.106964

[49] M. Olm , R. G. Stark , N. Beck , C. Röger , and R. Leidl , “Impact of Interventions to Reduce Overnutrition on Healthcare Costs Related to Obesity and Type 2 Diabetes: A Systematic Review,” Nutrition Reviews 78, no. 5 (2020): 412–435. 31769843 10.1093/nutrit/nuz070

[50] M. Oosterhoff , H. Bosma , O. C. P. van Schayck , S. Evers , C. D. Dirksen , and M. A. Joore , “A Systematic Review on Economic Evaluations of School‐Based Lifestyle Interventions Targeting Weight‐Related Behaviours Among 4‐12 Year Olds: Issues and Ways Forward,” Preventive Medicine 114 (2018): 115–122. 29959951 10.1016/j.ypmed.2018.06.015

[51] M. B. Pinheiro , K. Howard , C. Sherrington , et al., “Economic Evaluation of Physical Activity Mass Media Campaigns Across the Globe: A Systematic Review,” International Journal of Behavioral Nutrition and Physical Activity 19, no. 1 (2022): 107. 36028860 10.1186/s12966-022-01340-x PMC9419405

[52] B. Schwander , M. Hiligsmann , M. Nuijten , and S. Evers , “Systematic Review and Overview of Health Economic Evaluation Models in Obesity Prevention and Therapy,” Expert Review of Pharmacoeconomics & Outcomes Research 16, no. 5 (2016): 561–570. 27570095 10.1080/14737167.2016.1230497

[53] H. N. Q. Tran , E. McMahon , M. Moodie , and J. Ananthapavan , “A Systematic Review of Economic Evaluations of Health‐Promoting Food Retail‐Based Interventions,” International Journal of Environmental Research and Public Health 18, no. 3 (2021): 1356. 33540905 10.3390/ijerph18031356 PMC7908088

[54] A. M. Vargas‐Martínez , M. Romero‐Saldaña , and R. De Diego‐Cordero , “Economic Evaluation of Workplace Health Promotion Interventions Focused on Lifestyle: Systematic Review and meta‐Analysis,” Journal of Advanced Nursing 77, no. 9 (2021): 3657–3691. 33876454 10.1111/jan.14857

[55] P. White , H. Skirrow , A. George , and A. Memon , “A Systematic Review of Economic Evaluations of Local Authority Commissioned Preventative Public Health Interventions in Overweight and Obesity, Physical Inactivity, Alcohol and Illicit Drugs use and Smoking Cessation in the United Kingdom,” Journal of Public Health (Oxford, England) 40, no. 4 (2018): e521–e530. 29462346 10.1093/pubmed/fdy026

[56] S. B. Wolfenstetter and C. M. Wenig , “Economic Evaluation and Transferability of Physical Activity Programmes in Primary Prevention: A Systematic Review,” International Journal of Environmental Research and Public Health 7, no. 4 (2010): 1622–1648. 20617050 10.3390/ijerph7041622 PMC2872359

[57] N. Döring , N. Zethraeus , P. Tynelius , J. de Munter , D. Sonntag , and F. Rasmussen , “Economic Evaluation of PRIMROSE—A Trial‐Based Analysis of an Early Childhood Intervention to Prevent Obesity,” Frontiers in Endocrinology 9 (2018). 10.3389/fendo.2018.00104 PMC5861136 29593658

[58] A. Hayes , T. Lung , L. M. Wen , L. Baur , C. Rissel , and K. Howard , “Economic Evaluation of “Healthy Beginnings” an Early Childhood Intervention to Prevent Obesity,” Obesity 22, no. 7 (2014): 1709–1715. 24639421 10.1002/oby.20747

[59] A. Killedar , L. M. Wen , E. J. Tan , et al., “Economic Evaluation of the Communicating Healthy Beginnings Advice by Telephone Trial for Early Childhood Obesity Prevention,” Obesity 30, no. 11 (2022): 2256–2264. 36168138 10.1002/oby.23547 PMC9828236

[60] R. Sutherland , P. Reeves , E. Campbell , et al., “Cost Effectiveness of a Multi‐Component School‐Based Physical Activity Intervention Targeting Adolescents: The ‘Physical Activity 4 Everyone’ Cluster Randomized Trial,” International Journal of Behavioral Nutrition and Physical Activity 13, no. 1 (2016): 94. 27549382 10.1186/s12966-016-0418-2 PMC4994166

[61] M. Vieira and G. S. Carvalho , “Costs and Benefits of a School‐Based Health Intervention in Portugal,” Health Promotion International 34, no. 6 (2019): 1141–1148. 30339196 10.1093/heapro/day085

[62] J. Ananthapavan , P. K. Nguyen , S. J. Bowe , et al., “Cost‐Effectiveness of Community‐Based Childhood Obesity Prevention Interventions in Australia,” International Journal of Obesity 43, no. 5 (2019): 1102–1112. 30926947 10.1038/s41366-019-0341-0

[63] L. Gao , A. Flego , D. W. Dunstan , et al., “Economic Evaluation of a Randomized Controlled Trial of an Intervention to Reduce Office Workers' Sitting Time: The "Stand Up Victoria" Trial,” Scandinavian Journal of Work, Environment & Health 44, no. 5 (2018): 503–511. 10.5271/sjweh.3740 30078034

[64] R. An , H. Xue , L. Wang , and Y. Wang , “Projecting the Impact of a Nationwide School Plain Water Access Intervention on Childhood Obesity: A Cost‐Benefit Analysis,” Pediatric Obesity 13, no. 11 (2018): 715–723. 28941217 10.1111/ijpo.12236 PMC6062486

[65] J. Ananthapavan , G. Sacks , V. Brown , et al., “Priority‐Setting for Obesity Prevention‐The Assessing Cost‐Effectiveness of Obesity Prevention Policies in Australia (ACE‐Obesity Policy) Study,” PLoS ONE 15, no. 6 (2020): e0234804. 32559212 10.1371/journal.pone.0234804 PMC7304600

[66] S. H. Babey , S. Wu , and D. Cohen , “How can Schools Help Youth Increase Physical Activity? An Economic Analysis Comparing School‐Based Programs,” Preventive Medicine 69 (2014): S55–S60. 25456799 10.1016/j.ypmed.2014.10.013

[67] A. Basto‐Abreu , T. Barrientos‐Gutiérrez , D. Vidaña‐Pérez , et al., “Cost‐Effectiveness of the Sugar‐Sweetened Beverage Excise Tax in Mexico,” Health Affairs 38, no. 11 (2019): 1824–1831, 10.1377/hlthaff.2018.05469. 31682510

[68] S. Basu , H. Seligman , and J. Bhattacharya , “Nutritional Policy Changes in the Supplemental Nutrition Assistance Program: A Microsimulation and Cost‐Effectiveness Analysis,” Medical Decision Making 33, no. 7 (2013): 937–948. 23811757 10.1177/0272989X13493971

[69] W. Bemelmans , P. van Baal , W. Wendel‐Vos , et al., “The Costs, Effects and Cost‐Effectiveness of Counteracting Overweight on a Population Level. A Scientific Base for Policy Targets for the Dutch National Plan for Action,” Preventive Medicine 46, no. 2 (2008): 127–132. 17822752 10.1016/j.ypmed.2007.07.029

[70] V. Brown , M. Moodie , L. Cobiac , A. M. Mantilla Herrera , and R. Carter , “Obesity‐Related Health Impacts of Fuel Excise Taxation‐ An Evidence Review and Cost‐Effectiveness Study,” BMC Public Health 17, no. 1 (2017): 359. 28468618 10.1186/s12889-017-4271-2 PMC5415832

[71] V. Brown , J. Ananthapavan , L. Veerman , et al., “The Potential Cost‐Effectiveness and Equity Impacts of Restricting Television Advertising of Unhealthy Food and Beverages to Australian Children,” Nutrients 10, no. 5 (2018): 622. 29762517 10.3390/nu10050622 PMC5986502

[72] B. Edward , L. M. Alison , D. M. Yvette , G. B. Adrian , S. F. Brianna , and G. Nicholas , “The Cost‐Effectiveness of the *MobileMums* Intervention to Increase Physical Activity Among Mothers with Young Children: a Markov Model Informed by a Randomised Controlled Trial,” BMJ Open 5, no. 4 (2015): e007226. 10.1136/bmjopen-2014-007226 PMC4420940 25926145

[73] M. Cecchini , F. Sassi , J. A. Lauer , Y. Y. Lee , V. Guajardo‐Barron , and D. Chisholm , “Tackling of Unhealthy Diets, Physical Inactivity, and Obesity: Health Effects and Cost‐Effectiveness,” Lancet 376, no. 9754 (2010): 1775–1784. 21074255 10.1016/S0140-6736(10)61514-0

[74] L. J. Cobiac , T. Vos , and J. J. Barendregt , “Cost‐Effectiveness of Interventions to Promote Physical Activity: A Modelling Study,” PLoS Medicine 6, no. 7 (2009): e1000110. 19597537 10.1371/journal.pmed.1000110 PMC2700960

[75] L. J. Cobiac , T. Vos , and J. L. Veerman , “Cost‐Effectiveness of Interventions to Promote Fruit and Vegetable Consumption,” PLoS ONE 5, no. 11 (2010): e14148. 21152389 10.1371/journal.pone.0014148 PMC2994753

[76] L. J. Cobiac , K. Tam , L. Veerman , and T. Blakely , “Taxes and Subsidies for Improving Diet and Population Health in Australia: A Cost‐Effectiveness Modelling Study,” PLoS Medicine 14, no. 2 (2017): e1002232. 28196089 10.1371/journal.pmed.1002232 PMC5308803

[77] A. L. Cradock , J. L. Barrett , E. L. Kenney , et al., “Using Cost‐Effectiveness Analysis to Prioritize Policy and Programmatic Approaches to Physical Activity Promotion and Obesity Prevention in Childhood,” Preventive Medicine 95, no. Suppl (Suppl) (2017): S17–s27, 10.1016/j.ypmed.2016.10.017. 27773710 PMC10280789

[78] M. Crino , A. M. M. Herrera , J. Ananthapavan , et al., “Modelled Cost‐Effectiveness of a Package Size cap and a Kilojoule Reduction Intervention to Reduce Energy Intake From Sugar‐Sweetened Beverages in Australia,” Nutrients 9, no. 9 (2017): 983. 28878175 10.3390/nu9090983 PMC5622743

[79] J. Dallongeville , L. Dauchet , O. de Mouzon , V. Réquillart , and L. G. Soler , “Increasing Fruit and Vegetable Consumption: A Cost‐Effectiveness Analysis of Public Policies,” European Journal of Public Health 21, no. 1 (2011): 69–73. 20185530 10.1093/eurpub/ckq013

[80] J. P. Ekwaru , A. Ohinmaa , J. Dabravolskaj , K. Maximova , and P. J. Veugelers , “Cost‐Effectiveness and Return on Investment of School‐Based Health Promotion Programmes for Chronic Disease Prevention,” European Journal of Public Health 31, no. 6 (2021): 1183–1189. 34355754 10.1093/eurpub/ckab130 PMC8643402

[81] J. F. Emma , B. Mobeen , W. Khine , et al., “Cost‐Effectiveness of a Community‐Based Physical Activity Programme for Adults (Be Active) in the UK: An Economic Analysis Within a Natural Experiment,” British Journal of Sports Medicine 48, no. 3 (2014): 207. 22797421 10.1136/bjsports-2012-091202

[82] Y. Goryakin , A. Aldea , A. Lerouge , et al., “Promoting Sport and Physical Activity in Italy: A Cost‐Effectiveness Analysis of Seven Innovative Public Health Policies,” Annali di Igiene 31, no. 6 (2019): 614–625. 31616905 10.7416/ai.2019.2321

[83] M. M. Graziose , P. A. Koch , Y. C. Wang , H. Lee Gray , and I. R. Contento , “Cost‐Effectiveness of a Nutrition Education Curriculum Intervention in Elementary Schools,” Journal of Nutrition Education and Behavior 49, no. 8 (2017): 684–691. 27843129 10.1016/j.jneb.2016.10.006

[84] M. C. Gulliford , J. Charlton , N. Bhattarai , C. Charlton , and C. Rudisill , “Impact and Cost‐Effectiveness of a Universal Strategy to Promote Physical Activity in Primary Care: Population‐Based Cohort Study and Markov Model,” European Journal of Health Economics 15, no. 4 (2014): 341–351. 10.1007/s10198-013-0477-0 PMC3996351 23572044

[85] O. Huse , J. Ananthapavan , G. Sacks , et al., “The Potential Cost‐Effectiveness of Mandatory Restrictions on Price Promotions for Sugar‐Sweetened Beverages in Australia,” International Journal of Obesity 44, no. 5 (2020): 1011–1020. 31792336 10.1038/s41366-019-0495-9

[86] E. L. Kenney , A. L. Cradock , M. W. Long , et al., “Cost‐Effectiveness of Water Promotion Strategies in Schools for Preventing Childhood Obesity and Increasing Water Intake,” Obesity (Silver Spring) 27, no. 12 (2019): 2037–2045. 31746555 10.1002/oby.22615

[87] E. L. Kenney , R. S. Mozaffarian , M. W. Long , et al., “Limiting Television to Reduce Childhood Obesity: Cost‐Effectiveness of Five Population Strategies,” Childhood Obesity 17, no. 7 (2021): 442–448, 10.1089/chi.2021.0016. 33970695 PMC8568801

[88] A. Lal , A. M. Mantilla‐Herrera , L. Veerman , et al., “Modelled Health Benefits of a Sugar‐Sweetened Beverage tax Across Different Socioeconomic Groups in Australia: A Cost‐Effectiveness and Equity Analysis,” PLoS Medicine 14, no. 6 (2017): e1002326. 28654688 10.1371/journal.pmed.1002326 PMC5486958

[89] M. W. Long , M. Polacsek , P. Bruno , et al., “Cost‐Effectiveness Analysis and Stakeholder Evaluation of Two Obesity Prevention Policies in Maine, US,” Journal of Nutrition Education and Behavior 51, no. 10 (2019): 1177–1187, 10.1016/j.jneb.2019.07.005. 31402290

[90] A. Magnus , M. L. Moodie , M. Ferguson , L. J. Cobiac , S. C. Liberato , and J. Brimblecombe , “The Economic Feasibility of Price Discounts to Improve Diet in Australian Aboriginal Remote Communities,” Australian and New Zealand Journal of Public Health 40, no. S1 (2016): S36–S41. 26122947 10.1111/1753-6405.12391

[91] A. Magnus , M. M. Haby , R. Carter , and B. Swinburn , “The Cost‐Effectiveness of Removing Television Advertising of High‐Fat and/or High‐Sugar Food and Beverages to Australian Children,” International Journal of Obesity 33, no. 10 (2009): 1094–1102. 19652656 10.1038/ijo.2009.156

[92] A. M. Mantilla Herrera , M. Crino , H. E. Erskine , et al., “Cost‐Effectiveness of Product Reformulation in Response to the Health Star Rating Food Labelling System in Australia,” Nutrients 10, no. 5 (2018): 614. 29757979 10.3390/nu10050614 PMC5986494

[93] A. Mizdrak , K. Telfer , A. Direito , et al., “Health Gain, Cost Impacts, and Cost‐Effectiveness of a Mass Media Campaign to Promote Smartphone Apps for Physical Activity: Modeling Study,” JMIR mHealth and uHealth 8, no. 6 (2020): e18014. 32525493 10.2196/18014 PMC7317635

[94] M. Moodie , M. Haby , L. Galvin , B. Swinburn , and R. Carter , “Cost‐Effectiveness of Active Transport for Primary School Children ‐ Walking School bus Program,” International Journal of Behavioral Nutrition and Physical Activity 6, no. 1 (2009): 63. 19747402 10.1186/1479-5868-6-63 PMC2758827

[95] M. L. Moodie , R. C. Carter , B. A. Swinburn , and M. M. Haby , “The Cost‐Effectiveness of Australia's Active After‐School Communities Program,” Obesity 18, no. 8 (2010): 1585–1592. 19893504 10.1038/oby.2009.401

[96] M. Moodie , M. M. Haby , B. Swinburn , and R. Carter , “Assessing Cost‐Effectiveness in Obesity: Active Transport Program for Primary School Children‐‐TravelSMART Schools Curriculum Program,” Journal of Physical Activity & Health 8, no. 4 (2011): 503–515. 21597123 10.1123/jpah.8.4.503

[97] M. L. Moodie , J. K. Herbert , A. M. de Silva‐Sanigorski , et al., “The Cost‐Effectiveness of a Successful Community‐Based Obesity Prevention Program: The Be Active eat Well Program,” Obesity (Silver Spring) 21, no. 10 (2013): 2072–2080. 23554382 10.1002/oby.20472

[98] E. A. Over , G. W. Wendel‐Vos , M. van den Berg , et al., “Cost‐Effectiveness of Counseling and Pedometer Use to Increase Physical Activity in the Netherlands: A Modeling Study,” Cost Effectiveness and Resource Allocation 10, no. 1 (2012): 13, 10.1186/1478-7547-10-13. 23006466 PMC3495195

[99] E. Robinson , P. Nguyen , H. Jiang , et al., “Increasing the Price of Alcohol as an Obesity Prevention Measure: The Potential Cost‐Effectiveness of Introducing a Uniform Volumetric Tax and a Minimum Floor Price on Alcohol in Australia,” Nutrients 12, no. 3 (2020): 603, 10.3390/nu12030603. 32110864 PMC7146351

[100] L. Roux , M. Pratt , T. O. Tengs , et al., “Cost Effectiveness of Community‐Based Physical Activity Interventions,” American Journal of Preventive Medicine 35, no. 6 (2008): 578–588, 10.1016/j.amepre.2008.06.040. 19000846

[101] E. Rush , V. Obolonkin , S. McLennan , et al., “Lifetime Cost Effectiveness of a Through‐School Nutrition and Physical Programme: Project Energize,” Obesity Research & Clinical Practice 8, no. 2 (2014): e115–e122. 10.1016/j.orcp.2013.03.005 24743006

[102] S. J. te Velde , J. Lennert Veerman , N. I. Tak , J. E. Bosmans , K. I. Klepp , and J. Brug , “Modeling the Long Term Health Outcomes and Cost‐Effectiveness of two Interventions Promoting Fruit and Vegetable Intake Among Schoolchildren,” Economics and Human Biology 9, no. 1 (2011): 14–22. 20951103 10.1016/j.ehb.2010.09.001

[103] H. N. Q. Tran , A. Killedar , E. J. Tan , et al., “Cost‐Effectiveness of Scaling up a Whole‐Of‐Community Intervention: The Romp & Chomp Early Childhood Obesity Prevention Intervention,” Pediatric Obesity 17, no. 9 (2022): e12915. 35301814 10.1111/ijpo.12915 PMC9540361

[104] M. A. Colchero , J. Rivera‐Dommarco , B. M. Popkin , and S. W. Ng , “In Mexico, Evidence of Sustained Consumer Response Two Years After Implementing a Sugar‐Sweetened Beverage tax,” Health Affairs 36, no. 3 (2017): 564–571. 28228484 10.1377/hlthaff.2016.1231 PMC5442881

[105] D. Stern , N. Middaugh , M. S. Rice , et al., “Changes in Sugar‐Sweetened Soda Consumption, Weight, and Waist Circumference: 2‐Year Cohort of Mexican Women,” American Journal of Public Health 107, no. 11 (2017): 1801–1808. 28933937 10.2105/AJPH.2017.304008 PMC5637666

[106] J. C. de Ruyter , M. R. Olthof , J. C. Seidell , and M. B. Katan , “A Trial of Sugar‐Free or Sugar‐Sweetened Beverages and Body Weight in Children,” New England Journal of Medicine 367, no. 15 (2012): 1397–1406, 10.1056/NEJMoa1203034. 22998340

[107] A. J. Milat , A. Bauman , and S. Redman , “Narrative Review of Models and Success Factors for Scaling up Public Health Interventions,” Implementation Science 10, no. 1 (2015): 113. 26264351 10.1186/s13012-015-0301-6 PMC4533941

[108] F. Brundisini , H. T. V. Zomahoun , F. Légaré , et al., “Economic Evaluations of Scaling up Strategies of Evidence‐Based Health Interventions: A Systematic Review Protocol,” BMJ Open 11, no. 9 (2021): e050838. 10.1136/bmjopen-2021-050838 PMC8487175 34593499

[109] G. Sacks , J. Kwon , and J. Ananthapavan , “The Application of an Evidence Framework for Obesity Prevention at the Population‐Level,” Current Obesity Reports 9, no. 2 (2020): 150–158. 32266649 10.1007/s13679-020-00376-z

[110] D. Stokols , “Translating Social Ecological Theory Into Guidelines for Community Health Promotion,” American Journal of Health Promotion 10, no. 4 (1996): 282–298. 10159709 10.4278/0890-1171-10.4.282

[111] J. Ananthapavan , M. Moodie , and V. Brown , “Cost‐Effectiveness of Obesity Prevention and Treatment,” in Handbook of Obesity ‐ Volume 2.2, eds. P. T. Katzmarzyk , G. A. Bray , J. P. Kirwan , L. M. Redman , P. R. Schauer , and C. Bouchard (CRC Press, 2023).

[112] R. Cookson , A. J. Mirelman , S. Griffin , et al., “Using Cost‐Effectiveness Analysis to Address Health Equity Concerns,” Value in Health 20, no. 2 (2017): 206–212, 10.1016/j.jval.2016.11.027. 28237196 PMC5340318

