# Supplementary material for: How Is Scale Incorporated Into the Economic Evaluation of Interventions to Prevent Obesity or to Improve Obesity‐Related Risk Factors: A Systematic Scoping Review
Source: Obes Rev. 2025 May 21;26(9):e13942. doi: 10.1111/obr.13942 (PMC12318913; doi:10.1111/obr.13942)
Supplement: Supplementary file 1 — Data S1 Preferred Reporting Items for Systematic Review and Meta‐Analysis extension for scoping reviews (PRISMA‐ScR) (1). Data S2 Search strategies, by source. Data S3 Synthesis Without Meta‐Analysis guidelines (2). Data S4 Characteristics of studies included in the review. [file OBR-26-e13942-s001.pdf]

How is scale incorporated into the economic evaluation of interventions to prevent obesity or to improve obesity-related risk factors: a systematic scoping review

Supplementary materials

Carina Dalton MPH, Deakin University, Geelong, Australia, Deakin Health Economics, Institute for Health Transformation

Dr Marufa Sultana PhD, Deakin University, Geelong, Australia, Deakin Health Economics, Institute for Health Transformation

Kaitlyn McKenna MPH, Deakin University, Geelong, Australia, Deakin Health Economics, Institute for Health Transformation

Dr Vicki Brown PhD, Deakin University, Geelong, Australia, Deakin Health Economics, Institute for Health Transformation

Corresponding author:

Dr Vicki Brown* PhD Department of Health Economics, Deakin University, Burwood Victoria, Australia. Victoria.brown@deakin.edu.au. Phone (+61) 421 691 527; ORCID: 0000-0003-2891-9476

Keywords

Health economics, scale up, economic evaluation, obesity

SUPPLEMENTARY FILE 1- Preferred Reporting Items for Systematic Review and Meta-Analysis extension for scoping reviews (PRISMA-ScR)(1)

SECTION	ITEM	PRISMA-ScR CHECKLIST ITEM	REPORTED ON PAGE #
**TITLE**			
Title	1	Identify the report as a scoping review.	Title
**ABSTRACT**			
Structured summary	2	Provide a structured summary that includes (as applicable): background, objectives, eligibility criteria, sources of evidence, charting methods, results, and conclusions that relate to the review questions and objectives.	2-3
**INTRODUCTION**			
Rationale	3	Describe the rationale for the review in the context of what is already known. Explain why the review questions/objectives lend themselves to a scoping review approach.	4-5
Objectives	4	Provide an explicit statement of the questions and objectives being addressed with reference to their key elements (e.g., population or participants, concepts, and context) or other relevant key elements used to conceptualize the review questions and/or objectives.	5
**METHODS**			
Protocol and registration	5	Indicate whether a review protocol exists; state if and where it can be accessed (e.g., a Web address); and if available, provide registration information, including the registration number.	6
Eligibility criteria	6	Specify characteristics of the sources of evidence used as eligibility criteria (e.g., years considered, language, and publication status), and provide a rationale.	7-8
Information sources*	7	Describe all information sources in the search (e.g., databases with dates of coverage and contact with authors to identify additional sources), as well as the date the most recent search was executed.	6
Search	8	Present the full electronic search strategy for at least 1 database, including any limits used, such that it could be repeated.	6, Appendix 2
Selection of sources of evidence†	9	State the process for selecting sources of evidence (i.e., screening and eligibility) included in the scoping review.	7-8
Data charting process‡	10	Describe the methods of charting data from the included sources of evidence (e.g., calibrated forms or forms that have been tested by the team before their use, and whether data charting was done independently or in duplicate) and any processes for obtaining and confirming data from investigators.	9
Data items	11	List and define all variables for which data were sought and any assumptions and simplifications made.	9

SECTION	ITEM	PRISMA-ScR CHECKLIST ITEM	REPORTED ON PAGE #
Critical appraisal of individual sources of evidence§	12	If done, provide a rationale for conducting a critical appraisal of included sources of evidence; describe the methods used and how this information was used in any data synthesis (if appropriate).	NA
Synthesis of results	13	Describe the methods of handling and summarizing the data that were charted.	9
**RESULTS**			
Selection of sources of evidence	14	Give numbers of sources of evidence screened, assessed for eligibility, and included in the review, with reasons for exclusions at each stage, ideally using a flow diagram.	10-11
Characteristics of sources of evidence	15	For each source of evidence, present characteristics for which data were charted and provide the citations.	10-11
Critical appraisal within sources of evidence	16	If done, present data on critical appraisal of included sources of evidence (see item 12).	NA
Results of individual sources of evidence	17	For each included source of evidence, present the relevant data that were charted that relate to the review questions and objectives.	12-18
Synthesis of results	18	Summarize and/or present the charting results as they relate to the review questions and objectives.	10-18
**DISCUSSION**			
Summary of evidence	19	Summarize the main results (including an overview of concepts, themes, and types of evidence available), link to the review questions and objectives, and consider the relevance to key groups.	18-21
Limitations	20	Discuss the limitations of the scoping review process.	21
Conclusions	21	Provide a general interpretation of the results with respect to the review questions and objectives, as well as potential implications and/or next steps.	21-22
**FUNDING**			
Funding	22	Describe sources of funding for the included sources of evidence, as well as sources of funding for the scoping review. Describe the role of the funders of the scoping review.	1

SUPPLEMENTARY FILE 2 – Search strategies, by source

The following databases were searched on 17th July 2023

EBSCOhost Research Databases and the Cochrane Library.

In EBSCO Research Databases

- Academic Search Complete;
- CINAHL Complete;
- EconLit;
- MEDLINE Complete
-

Search modes - Boolean/Phrase

S61 S56 AND S58 AND S60

S60 S57 OR S59

S59 S39 OR S40 OR S41 OR S42 OR S43 OR S44 OR S45 OR S46 OR S47 OR S48 OR S49 OR S50 OR S51 OR S52 OR S53 OR S54 OR S55

S58 S24 OR S25 OR S26 OR S27 OR S28 OR S29 OR S30 OR S31 OR S32 OR S33 OR S34 OR S35 OR S36 OR S37 OR S38

S57 S10 OR S11 OR S12 OR S13 OR S14 OR S15 OR S16 OR S17 OR S18 OR S19 OR S20 OR S21 OR S22 OR S23

S56 S1 OR S2 OR S3 OR S4 OR S5 OR S6 OR S7 OR S8 OR S9

S55 TI sleep* OR AB sleep*

S54 TI "screen based" OR AB "screen based"

S53 TI “energy balance” OR AB “energy balance”

S52 TI screentime OR AB screentime

S51 TI “screen time” OR AB “screen time”

S50 TI "sedentary lifestyle*" OR AB "sedentary lifestyle*

S49 TI “sedentary behavi*” OR AB “sedentary behavi*”

S48 TI exercis* OR AB exercis*

S47 TI “physical* activ*” OR AB “physical* activ*”

S46 TI ‘weight” OR AB “weight”

S45 TI “energy- intake” OR AB “energy-intake”

S44 TI “energy intake” OR AB “energy intake”

S43 TI vegetable* OR AB vegetable*

S42 TI fruit* OR AB fruit*

S41 TI eat* OR AB eat*

S40 TI nutri* OR AB nutri*

S39 TI diet* OR AB diet*

S38 TI “economic* model*" OR AB “economic* model*"

S37 TI "health economic*" OR AB "health economic*"

S36 TI "economic* analys*" OR AB "economic* analys*"

S35 TI "economic* evaluat*" OR AB "economic* evaluat*"

S34 TI "cost-consequence" OR AB "cost-consequence"

S33 TI "cost-minimi*" OR AB "cost-minimi*"

S32 TI "cost minimi*" OR AB "cost minimi*"

S31 TI "cost-utili*" OR AB "cost-utili*"

S30 TI "cost benefit*" OR AB "cost benefit*"

S29 TI "cost-benefit*" OR AB "cost-benefit*"

S28 TI "cost effect*" OR AB "cost effect*”

S27 TI "cost-effect*" OR AB "cost-effect*”

S26 (MH "Cost-Benefit Analysis")

S25 TI Economic* OR AB Economic*

S24 TI “Economic Evaluation” OR AB “Economic Evaluation”

S23 (MH "Weight Loss+")

S22 (MH "Overweight")

S21 (MH "Obesity+")

S20 (MH "Obesity+") OR

S19 TI adipos* OR AB adipos*

S18 TI “weight loss” OR AB “weight loss”

S17 TI “weight gain” OR AB “weight gain”

S16 TI “body mass” OR AB “body mass”

S15 TI BMI OR AB BMI

S14 TI “over-weight” OR AB “over-weight”

S13 TI “over weight” OR AB “over weight”

S12 TI weight OR AB weight

S11 TI overweight OR AB overweight

S10 TI overweight OR AB overweight

S9 TI “Meta analysis” OR AB “Meta analysis”

S8 (MH "Review Literature as Topic+")

S7 (MH "Meta-Analysis as Topic+")

S6 TI “umbrella review” OR AB “umbrella review”

S5 TI “meta-synthesis” OR AB “meta-synthesis”

S4 TI “umbrella review” OR AB “umbrella review”

S3	PT “meta-analysis"OR TI “systematic literature review” OR AB “systematic literature review”
S2	TI “Meta-analysis” OR AB “Meta-analysis”
SI	TI “Systematic review” OR AB “Systematic review”

Cochrane Library

ID	Search
#1	MeSH descriptor Obesity explode all trees
#2	MeSH descriptor Body mass index explode all trees
#3	MeSH descriptor Overweight explode all trees
#4	Mesh descriptor weight gain explode all trees
#5	(adipos):ti,ab,kw
#6	(body mass):ti,ab,kw
#7	(obes*):ti,ab,kw
#8	#1 OR #2 OR #3 OR #4 OR #5 OR #6 OR #7
#9	Mesh descriptor Cost-Benefit Analysis explode all trees
#10	(economic evaluation):ti,ab,kw
#11	(“cost effectiv*”):ti,ab,kw
#12	(cost-effect*):ti,ab,kw
#13	(“cost benefit”):ti,ab,kw
#14	(“cost utili*”):ti,ab,kw
#15	(cost-utili*):ti,ab,kw
#16	(“cost minimi*”):ti,ab,kw
#17	(cost-minimi*):ti,ab,kw
#18	(“cost consequence”):ti,ab,kw
#19	(cost-consequence):ti,ab,kw

#20 (“economic* evaluat*”):ti,ab,kw

#21 (“economic* analys*”):ti,ab,kw

#22 (“health economics”):ti,ab,kw

#23 (“economic* model*”):ti,ab,kw

#24 (economic*):ti,ab,kw

#25 (cost*):ti,ab,kw

#26 (modell*):ti,ab,kw

#27 #9 OR #10 OR #11 OR #12 OR #13 OR #14 OR #15 OR #16 OR #17 OR #18 OR #19 OR #20 OR #21 OR #22 OR #23 OR #24 OR #25 OR #26

#28 (diet*):ti,ab,kw

#29 (nutri*):ti,ab,kw

#30 (eat*):ti,ab,kw

#31 (fruit*):ti,ab,kw

#32 (vegetable*):ti,ab,kw

#33 (energy NEXT intake):ti,ab,kw

#34 (physical NEXT activ*):ti,ab,kw

#35 (exercis*):ti,ab,kw

#36 (sedentary NEXT behavio*):ti,ab,kw

#37 (sedentary NEXT lifestyle*):ti,ab,kw

#38 (screen NEXT time):ti,ab,kw

#39 (screentime):ti,ab,kw

#40 (“energy balance”):ti,ab,kw

#41 (screen NEXT based):ti,ab,kw

#42 (sleep):ti,ab,kw

#43 (sedentary):ti,ab,kw

#44 (screen):ti,ab,kw

#45 (physical):ti,ab,kw

#46 (“weight control”):ti,ab,kw

#47 (“weight loss”):ti,ab,kw

#48	#28 OR #29 OR #29 OR #30 OR #31 OR #32 OR #33 OR #34 OR #35 OR #36 OR #37 OR #38 OR #39 OR #40 OR #41 OR #42 OR #43 OR #44 OR #45 OR #46 OR #47
#49	#48 OR #8
#50	#49 AND #27

SUPPLEMENTARY FILE 3 – Synthesis Without Meta-Analysis guidelines(2)

**SWiM reporting item**	**Item description**	**Page reported**	**Other***
Methods			
1 Grouping studies for synthesis	1a) Provide a description of, and rationale for, the groups used in the synthesis (e.g., groupings of populations, interventions, outcomes, study design)	6-9	
	1b) Detail and provide rationale for any changes made subsequent to the protocol in the groups used in the synthesis	NA	
2 Describe the standardised metric and transformation used	Describe the standardised metric for each outcome. Explain why the metric(s) was chosen, and describe any methods used to transform the intervention effects, as reported in the study, to the standardised metric, citing any methodological guidance consulted	NA	
3 Describe the synthesis methods	Describe and justify the methods used to synthesise the effects for each outcome when it was not possible to undertake a meta-analysis of effect estimates	6-9	
4 Criteria used to prioritise results for summary and synthesis	Where applicable, provide the criteria used, with supporting justification, to select the	NA	

	particular studies, or a particular study, for the main synthesis or to draw conclusions from the synthesis (e.g., based on study design, risk of bias assessments, directness in relation to the review question)		
5 Investigation of heterogeneity in reported effects	State the method(s) used to examine heterogeneity in reported effects when it was not possible to undertake a meta-analysis of effect estimates and its extensions to investigate heterogeneity	NA	
6 Certainty of evidence	Describe the methods used to assess certainty of the synthesis findings	NA	
7 Data presentation methods	Describe the graphical and tabular methods used to present the effects (e.g., tables, forest plots, harvest plots). Specify key study characteristics (e.g., study design, risk of bias) used to order the studies, in the text and any tables or graphs, clearly referencing the studies included	Table 2, Appendix 4	
Results			
8 Reporting results	For each comparison and outcome, provide a description of the synthesised findings, and the certainty of the findings. Describe the result in language	10-18	

	that is consistent with the question the synthesis addresses, and indicate which studies contribute to the synthesis		
Discussion			
9 Limitations of the synthesis	Report the limitations of the synthesis methods used and/or the groupings used in the synthesis, and how these affect the conclusions that can be drawn in relation to the original review question	21	

SUPPLEMENTARY FILE 4 – Characteristics of studies included in the review

#	An et al.(3)	
1	Study aim	To estimate the societal cost and benefit of an expansion of a water access intervention that promotes lunchtime plain water consumption by placing water dispensers in New York school cafeterias to all schools nationwide
	Country	United States
	Currency unit and year	USD 2016
	Study design	Modelled CBA
	Setting	Schools (primary, secondary)
	Intervention	Potential nationwide expansion of a program placing water dispensers in school cafeterias (water dispenser purchase and annual maintenance expenses).
	Comparator	No intervention
	Target population	75,227,000 school-aged children, from 129,189 public and private schools in the US.
	Perspective	Societal
	Time horizon	Lifetime
	Model specification (if applicable)	A Markov chain decision model built and simulated using TreeAge Pro 2017.<br>The decision model simulated two possible events using Markov chains: the "experiment" event of placing water dispensers at lunch times in school cafeterias nationwide, or the "control" event of no action. Both events were simulated in Markov chains where subjects were exposed to an annual, age- and sex-specific risk of death, with survivors gaining one year of life and corresponding costs, if any (costs only pertained to overweight/obese subjects). This process was repeated until all subjects died.
	Measurement and valuation of outcomes	Pre-post studies of pilot. The risk reduction of overweight was based on one study from the literature.

	Measurement and valuation of resources and cost	One time cost of purchasing water dispenser and annual maintenance expenses (cleaning, setting up the water jet, refilling the water and cups as necessary 10 minutes per day by staff), taken from study in the literature. Annual medical cost associated with adult overweight and obesity using evidence from the literature. The percentage of medical cost of adult overweight or obesity over total economic cost using evidence from the literature.
	Discount rate	3%
	Methods for uncertainty	Monte Carlo simulation (10,000 runs), with inputs sampled at random from probability distribution functions (normal distributions).
	Methods for sensitivity	One-way sensitivity analyses included discount rate (0%, 6%), risk reduction from intervention, medical costs of overweight and obesity in adulthood, probabilities of overweight children becoming overweight or obese adults, prevalence of overweight and obesity, cost of water dispensers (using low and high estimates).
	Brief summary of results (incl. sensitivity results)	The estimated incremental cost of the school-based water access intervention was $18 per student, and the corresponding incremental benefit was $192, resulting in a net benefit of $174 per student.<br><br>A 6% discount rate was associated with a $104 benefit per student, and a 0% rate increased the benefit to $342. Reductions in childhood overweight, particularly in boys, also impacted the net benefit, ranging from $102 per student for a 0.3% reduction to $247 for a 1.5% reduction, while other factors like medical costs and probabilities of overweight children becoming obese had smaller effects.
	Limitations	Underestimation of other related benefits of the interventions, and the intervention cost.<br>Sub-group analyses not undertaken. Simplistic analysis of costs and risk over the lifetime, assuming fixed incremental healthcare cost attributed to overweight and obesity across ages among adults and fixed overweight reduction across ages among children.

	Funding source	Funded by the National Institute of Health (NIH, 1R01HD064685-01A1 and U54HD070725), and the National Aeronautics and Space Administration
	Conflicts of interest	None declared
**Ananthapavan et al.(4)**		
2	Study aim	To examine, from a limited societal perspective, the cost-effectiveness of CBIs defined as a program of community-level strategies to promote healthy eating and physical activity for Australian children (aged 5–18 years).
	Country	Australia
	Currency unit and year	AUD 2010
	Study design	Modelled CUA
	Setting	Community
	Intervention	Hypothetical CBI based on a literature review
	Comparator	No intervention
	Target population	2.3 million students across 5913 government primary and secondary schools in Australia
	Perspective	Limited societal
	Time horizon	Lifetime
	Model specification (if applicable)	Multiple cohort Markov model used to estimate the short and long-term health outcomes resulting from changes in BMI. The model estimated the incidence, prevalence, and mortality of nine diseases causally related to elevated BMI over the life course (type 2 diabetes, stroke, hypertensive heart disease, ischaemic heart disease, osteoarthritis of the hip and knee, and several cancers (kidney, endometrial, breast, and colorectal). Each of these diseases were modelled with four health states (healthy, diseased, dead due to disease, dead from other causes). Transitions between states were determined by incidence (calculated using the potential impact fraction using relative risk of disease related to BMI), prevalence, case fatality rates, and all-cause mortality. The health states were weighted by disability weights from the Global Burden of

		Disease 2010 Study. The time spent in each health state was aggregated to estimate health-adjusted life years (HALYs).
	Measurement and valuation of outcomes	Effectiveness of intervention as change in BMIz from a meta-analysis of the literature – modelled to HALYs
	Measurement and valuation of resources and cost	The unit costs and resource use for implementing the generic CBI program were taken from multiple sources including process data from trials and other published CBIs. Included average cost per nutrition and physical activity strategy implemented within schools, the average cost of physical changes to the school (e.g., a school garden or water fountain) and changes in the community (e.g., improving food quality at local restaurants). The cost of central co-ordination and promotion was also costed. Healthcare cost-savings from cases of disease averted using published data.
	Discount rate	3%
	Methods for uncertainty	Monte-Carlo simulation using the Excel add-in software, Ersatz
	Methods for sensitivity	One-way sensitivity analyses tested variables including effect size, target population, and intervention intensity.<br><br>Five scenario analyses included:1)"intensive intervention" – including more strategies costed for the intervention, in children at both primary and secondary schools, 2)"best case"- including less strategies in the costing but a higher effect size of -0.08BMIz, 3)including evaluation costs, 4) in children in primary schools only, 5)incorporating a 5% effect decay annually.<br><br>Threshold analysis was undertaken to assess the duration of effect required for the intervention to be considered cost-effective.
	Brief summary of results (incl. sensitivity results)	The estimated net cost of implementing CBIs across all local government areas (LGAs) in Australia was AUD426M (95% UI: AUD3M to AUD823M) over 3 years. This resulted in 51,792 HALYs gained (95% UI: 6816 to 96,972) over the lifetime of the cohort. The mean ICER was

		AUD8155 per HALY gained (95% UI: AUD237 to AUD81,021), with a 95% probability of being cost-effective at a willingness to pay threshold of AUD50,000 per HALY.<br><br>The intervention remained cost-effective when the intervention intensity and the effect size were varied. If only primary schools implemented the CBI, the intervention was dominant (95% UI: dominant to AUD30,448). When the intervention effect was decayed by 5% each year, the CBIs were no longer cost-effective.
	Limitations	Included studies in meta- analysis were highly heterogeneous; the use of a limited societal perspective meant down- stream costs (and benefits) not fully captured; assumptions made in modelling.
	Funding source	National Health and Medical Research Council, Centre of Research Excellence on Obesity Policy and Food Systems (Grant number 1041020)
	Conflicts of interest	None declared
**Ananthapavan et al.(5)[#]**		
3	Study aim	To assess the economic credentials of 16 obesity prevention policies across multiple sectors and areas of governance for the Australian setting.
	Country	Australia
	Currency unit and year	AUD 2010
	Study design	Modelled CUA
	Setting	Interventions across multiple settings; intervention dependent
	Intervention	The interventions assessed were: financial incentives for weight loss provided by private health insurers, menu kilojoule labelling on fast food, national mass media campaign related to sugar-sweetened beverages, school-based intervention to reduce sedentary behaviour/increase physical activity, supermarket shelf tags on healthier products, alcohol price increase: uniform volumetric tax (also reported in Robinson et al.(6) and so not duplicated here), community–based

		interventions (also reported in Ananthapavan et al.(4) and so not duplicated here), fuel excise: 10c per litre increase (also reported in Brown et al.(7) and so not duplicated here), workplace intervention to reduce sedentary behaviour (also reported in Gao et al.(8) and so not duplicated here), restrictions on price promotions of sugar- sweetened beverages (also reported in Huse et al.(9) and so not duplicated here), restricting television advertising of unhealthy foods (also reported in Brown et al.(10) and so not duplicated here), package size cap on sugar-sweetened beverages (also reported in Crino et al.(11) and so not duplicated here), reformulation to reduce sugar in sugar-sweetened beverages (also reported in Crino et al.(11) and so not duplicated here), sugar-sweetened beverages tax– 20%, (also reported in Lal et al.(12) and so not duplicated here), reformulation in response to the HSR system (also reported in Mantilla Herrera et al.(13) and so not duplicated here).
	Comparator	No intervention
	Target population	Intervention specific populations exposed to intervention and listed, specific details on population figures not provided.<br><br>Financial incentives for weight loss provided by private health insurers- Australian 18–100 year olds<br><br>Menu kilojoule labelling on fast food- Australian 2–100 year olds<br><br>National mass media campaign related to sugar-sweetened beverages- Australian 18–100 year olds-<br><br>School-based intervention to reduce sedentary behaviour/increase physical activity- Australian 8–9 year olds<br>Supermarket shelf tags on healthier products – Australian 2–100 year olds
	Perspective	Limited societal
	Time horizon	Lifetime
	Model specification (if applicable)	A multi- state life table Markov cohort model (ACE-Obesity project) estimated the effect of intervention-related changes in one or more risk

		factors on the incidence of diseases related to the relevant risk factors over the lifetime of the population. The model included nine diseases (kidney cancer, colorectal cancer, endometrial cancer, breast cancer, type 2 diabetes, hypertensive heart disease, ischaemic heart disease, stroke, and osteoarthritis of the hip and knee). All diseases were causally related to BMI and five were related to physical activity (colorectal cancer, breast cancer, type 2 diabetes, ischaemic heart disease, and stroke).
	Measurement and valuation of outcomes	Modelled intervention effect, with limited information on sources of data for modelling.<br><br>Financial incentives for weight loss- BMI-HALYs<br><br>Menu kilojoule labelling on fast food- BMI-HALYS<br><br>National mass media campaign related to sugar-sweetened beverages- BMI-HALYs<br><br>School-based intervention to reduce sedentary behaviour/increase physical activity- BMI/PA-HALYs<br>Supermarket shelf tags on healthier products – BMI-HALYs
	Measurement and valuation of resources and cost	Cost components varied by intervention, unit costs sourced from administrative databases.<br>Healthcare cost-savings from cases of disease averted using published data.
	Discount rate	3%
	Methods for uncertainty	Ersatz software was used to incorporate parameter uncertainty. Two thousand model iterations with varying parameter values presented uncertainty intervals around the outputs.
	Methods for sensitivity	Various univariate sensitivity analyses were undertaken to test the key assumptions related to specific interventions.
	Brief summary of results (incl. sensitivity results)	All interventions were found to be cost-effective. Eleven interventions were dominant.

	Limitations	The diversity of interventions resulted in variations in the evidence base for modelling assumptions. Interventions with higher-quality evidence were modeled using more conservative assumptions. Each intervention's modeling involved extensive analysis of uncertainty and scenarios, to provide additional context for result interpretation and to address uncertainty in the evidence base.<br><br>Only the base case ICER was presented and the model used 2010 as the base year, which did not reflect new data that emerged while the study was undertaken over six years.<br>The study also did not take into account coordinated efforts but modelled only the impact of single interventions.<br>There was also a lack of evidence for interventions in specific sectors such as agriculture, built environment, environment and trade that limits the sectors represented in the evaluations.
	Funding source	This research activity was supported by the National Health and Medical Research Council funded Centre of Research Excellence in Obesity Policy and Food Systems (APP1041020)
	Conflicts of interest	None declared
**Babey et al.(14)**		
4	Study aim	Assessing school-based opportunities (after-school programs, before-school programs, PE classes, extended-day PE, and short physical activity breaks during the school day) to improve physical activity for youth.
	Country	United States
	Currency unit and year	USD 2012
	Study design	Modelled CEA
	Setting	Schools
	Intervention	1. After-school program (programs that are either fee-based or subsidised and either on-site or off-site, typically from 3pm to 6pm)

		2. PE classes and extended-day PE by 40 mins to 60 mins (mandatory)<br>3. In-class activity with two 10 mins structured physical activities, implemented by playing exercise videos (such as Instant Recess® or Take10!®) with or without extended school day by 20 mins<br>4. Before-school activity program that offers students to participate in physical activities, informal sports or interscholastic sports
	Comparator	Not clearly stated
	Target population	School age children.<br>Extended school day, in-class activity, before-school program – assumed mandatory and so reached 100% of students; extended school day middle and high school only.<br>After school programs - Based on published research on reach, approx. 17% of elementary, 12% of middle school and 7% of high school students
	Perspective	Not clearly stated
	Time horizon	1 year
	Model specification (if applicable)	Not clearly stated - crude mathematical modelling
	Measurement and valuation of outcomes	The cost efficiency of different interventions were assessed by estimating the time present in physical activities and intensity levels (moderate physical activity or vigorous physical activity) per student based on published literature. The effectiveness was standardised by calculating higher MET intensities, expressed as MET-hours gained, per person per day resulting from the intervention. The study assumed all physical activities were moderate physical activity or vigorous physical activity

		and used a MET value of 4.5 to provide standardised metric for comparison.
	Measurement and valuation of resources and cost	The total annual cost per participating child to operate the program on an on-going basis was considered for the program cost. School year length was assumed to be 180 days with an average of 30 students per class.<br><br>Where applicable, the cost figures were estimated from published cost analyses or imputed based upon resource utilization. Focus of operating costs for the programs included personnel costs, supplies and materials, equipment, program overhead costs such as facilities costs (30% of direct costs), and transportation costs.
	Discount rate	Not reported
	Methods for uncertainty	Not reported
	Methods for sensitivity	Not reported
	Brief summary of results (incl. sensitivity results)	In terms of reach and cost per student, two programs were superior: extending the school day with mandatory PE participation and Short (10-minute) physical activity breaks during regular classroom hours. After-school programs had higher costs and a smaller reach. Before-school programs did not seem feasible.
	Limitations	The study did not include development and implementation costs or societal-level costs. It only focused on operating costs. Potential societal benefits were also not included, such as reduced health care costs due to increase level of physical activity. The barriers to implementing each strategy were also not incorporated into the cost-effectiveness estimates as it could not be quantified.
	Funding source	Commissioned by the Robert Wood Johnson Foundation through its Active Living Research program and funded in part by grant R01HL104213 from the National Heart Lung and Blood Institute.

	Conflicts of interest	None declared
**Barrett et al.(15)**		
5	Study aim	To estimate the cost effectiveness of an “active PE” policy implemented nationally.
	Country	United States
	Currency unit and year	USD 2014
	Study design	Modelled CEA
	Setting	Policy, schools
	Intervention	“Active PE” policy to be included in the state’s elementary schools existing PE classes with 50% of PE time be devoted to MVPA
	Comparator	Current practice
	Target population	Children aged 6–11 years. Estimated to reach 75% of the 2015 U.S. population aged 6–11 years, 18.5 million children aged 6–11 years attending more than 47,000 public elementary schools
	Perspective	A modified societal perspective
	Time horizon	10 years
	Model specification (if applicable)	CHOICES model - a Microsoft Excel–based Markov cohort model based on ACE-Obesity, but modified and replicated in a compiled programming language (Java) for CHOICES.
	Measurement and valuation of outcomes	Expected effectiveness of the active PE policy on per capita MVPA was modeled using results from a recent meta-analysis of active PE trials.<br><br>MVPA minutes were converted to MET-hours gained<br><br>Two studies identified from >600 reviewed according to the CHOICES evidence review protocol were used to estimate the change in BMI expected from a change in MVPA.

		One study provided evidence of change in BMI resulting from a change in objectively measured MVPA in an RCT of a school-based physical activity intervention with no co-interventions.<br><br>Another study provided evidence of the change in BMI resulting from change in MVPA using a 6-year longitudinal observational design. Based on results from the two studies, each 1-minute increase in regular daily MVPA was estimated to result in an average per capita BMI reduction of 0.023.
	Measurement and valuation of resources and cost	1-year and 10-year costs of implementing the policy were estimated by considering the cost of the resources for sustaining implementation. Cost categories included training teachers and principals, equipment, co-ordination costs. Resources required to sustain regular implementation of the active PE policy were identified, based mostly on assumptions, previous program estimates and informed choices (consult with stakeholders for some costs).
	Discount rate	3%
	Methods for uncertainty	Probabilistic sensitivity analyses were conducted by simultaneously sampling values from within specified distributions using Monte Carlo simulations in @RISK, version 6.1.2, to estimate physical activity and BMI changes over 10,000 iterations and in Java to estimate 10-year outcomes over 1,000,000 iterations.
	Methods for sensitivity	Included a secondary scenario, in which more PE time was added as a result of the intervention (based on a positive side effect seen within a published study). Effect size incorporated additional time in MVPA, and extra costs of training PE specialists to cover instruction minutes was added.<br><br>Univariate sensitivity analyses included 100% implementation amongst trained teachers; modelling MVPA to BMI using different published method; assuming all children attended PE for 150 minutes per week;

		costs of full training to additional PE specialists needed to provide PE at the current teacher-student ratio included; exclusion of newly hired PE specialists as start-up costs.
	Brief summary of results (incl. sensitivity results)	Implementing an elementary school active PE policy nationally would increase MVPA by 1.87 minutes per 30-minute PE class in the first year, costing $70.7 million. The cost per BMI unit reduction after 2 years would be $401. Over the period from 2015 to 2025, the policy would cost $235 million and reduce healthcare costs by $60.5 million.<br><br>Results from sensitivity analyses showed that if active PE were implemented by 100% of trained teachers, the resulting increase in children benefiting would reduce the cost per BMI unit reduced to $287 (95% UI1⁄4$108, $2,170), and healthcare cost savings over 10 years would rise to $84.4 million.<br><br>If additional minutes of PE at 50% MVPA were provided by trained PE specialists (the SPARK effect, secondary scenario), mean per capita MVPA would increase by 629 minutes per year (95% UI=389, 925) among the population reached at an estimated annual intervention cost of $78.5 million (95% UI=$57.5 million, $105 million).
	Limitations	Optimistic assumption that the intervention-related reduction in BMI after 2 years persists for 10 years. The 2-year BMI effect could also underestimate the actual impact, given continuous exposure during elementary school and not fully capture the potential long-term positive outcomes of childhood physical activity increase, such as lifelong habit changes.
	Funding source	In part by grants from the Robert Wood Johnson Foundation (number 66284); the Donald and Sue Pritzker Nutrition and Fitness Initiative; and the JPB Foundation and is a product of a Centre for Research Excellence in Obesity Policy and Food Systems supported by the Australian National Health and Medical Research Centre (grant number 1041020) and a Prevention Research Center supported by Cooperative Agreement

		U48/DP00064-00S1 from CDC, including the Nutrition and Obesity Policy, Research and Evaluation Network.
	Conflicts of interest	None declared
**Basto-Abreu et al.(16)**		
6	Study aim	The study aimed to assess the impact of a sugar-sweetened beverage tax in Mexico over ten years using a calibrated cohort simulation model
	Country	Mexico
	Currency unit and year	Pesos then translated into US dollars using the average 2014 conversion rate (1 dollar = 13.3718 pesos)
	Study design	Modelled CUA
	Setting	Policy
	Intervention	Implementation of an SSB excise tax to reduce consumption and prevent obesity-related diseases
	Comparator	Do nothing
	Target population	2014 Mexican population aged 2-100 years
	Perspective	Not stated
	Time horizon	10 years
	Model specification (if applicable)	The CHOICES model, and adapted to the Mexican context. It is a cohort simulation model that simulates changes in the BMI distribution of the Mexican population aged 2–100 over a ten-year period. The cohort is followed until death or reaching 100 years of age.
	Measurement and valuation of outcomes	Modelled intervention effect from average annual decrease of SSB purchases two years after implementation of the tax from published study-assuming purchase close proxy to consumption- change in BMI using evidence from published studies
	Measurement and valuation of resources and cost	Cost of implementation of the tax, divided into planning and development costs and operating costs (steady state). Costs were estimated as a fraction of the finance ministry's budget based on the months dedicated by the personnel involved to each activity; indirect costing approach to estimate based on the proportion of revenue collected and the potential number of payers of the taxes; planning and

		development costs using information from interview with staff from Ministry of Finance.
	Discount rate	3%
	Methods for uncertainty	Probabilistic sensitivity analyses, sampling from pre-determined distributions.
	Methods for sensitivity	Application of a 5% discount rate
	Brief summary of results (incl. sensitivity results)	At the population level, the current sugar-sweetened beverage tax would add 918 life-years and 55,300 QALYs, avert 5,840 DALYs, and save an estimated $91.6 million over ten years. With a 3% discount rate, the tax was expected to save $3.98 in health care costs per dollar spent on implementation, and a gain of $3.65 per dollar spent with a 5% discount rate.
	Limitations	Use of 24-hour dietary recall may not fully capture actual caloric intake, and may potentially result in conservative estimates of the tax's impact.<br><br>The study was also unable to assess changes in consumption after the tax due to limitations in survey data.
	Funding source	In part by grants from the Robert Wood Johnson Foundation (Grant No. 77202) and Bloomberg Philanthropies (grant to the National Institute of Public Health, Mexico). Tonatiuh Barrientos-Gutiérrez also received support from Harvard University through the Lown Scholars Program
	Conflicts of interest	None declared
**Basu et al.(17)**		
7	Study aim	To estimate the health effects and cost-effectiveness of banning or taxing SSBs or subsidising fruits and vegetables purchased with SNAP.
	Country	United States
	Currency unit and year	USD 2012
	Study design	Modelled CUA
	Setting	Policy

	Intervention	1) A restriction or monetary penalty for purchasing SSBs with SNAP benefits, 2) a subsidy or reward for purchasing fruits and vegetables using SNAP dollars, and 3) overall increases in SNAP benefit levels.
	Comparator	No intervention
	Target population	US adults aged 25 to 64 years old
	Perspective	Governmental perspective
	Time horizon	10 years
	Model specification (if applicable)	A stochastic, discrete-time microsimulation model that simulates food intake among US adults. Five outcomes modelled: Alternative Healthy Eating Index, food security score, BMI, diabetes risk, CVD mortality risk.
	Measurement and valuation of outcomes	Effects of hypothetical interventions estimated by decreasing or increasing the probability of food intake, using intake elasticities calculated from administrative data (own and cross-intake elasticities). Changes in calorie intake modelled to changes in BMI using validated model. Diabetes risk estimated from change in take (increased relative risk from more servings of SSBs per day using estimate from the literature; decreased relative risk from more servings of vegetables using estimate from the literature (meta-analysis of cohort studies)). Changes in CVD mortality using changes in Alternative Healthy Eating Index and diabetes status (Change in risk of mortality from myocardial infarction or stroke given a change in Alternative Healthy Eating Index score: Whitehall II study. Changes in risk of mortality from myocardial infarction or stroke based on change in diabetes status: Framingham Study).<br><br>QALYs lost for each year of diabetes and CVD-related disability from the literature.
	Measurement and valuation of resources and cost	The cost of subsidies or taxes were estimated by multiplying the total demand (including the change in demand due to the subsidy or tax) by

		the cost of the subsidy or tax per unit demanded. The deadweight loss from taxes needed to finance a subsidy was also included.<br><br>Average medical costs per year for diabetes: 2010 Medical Expenditure Panel Survey<br><br>Average medical costs per year during the period of disability and associated with death from CVD: 2010 Medical Expenditure Panel Survey
	Discount rate	3%
	Methods for uncertainty	10,000 repeated replications conducted by Monte Carlo sampling to generate mean and 95% confidence interval estimates from the simulation from the distributions of usual food intake, intake elasticity costs, and QALYs.
	Methods for sensitivity	Sensitivity analyses varied parameter inputs, including SNAP participation rates, food consumption, and elasticities (using partial rank correlation coefficients).
	Brief summary of results (incl. sensitivity results)	Interventions cost-effective: Banning SSB purchases using SNAP benefits $2900 per QALY saved. A tax on SSBs would produce higher cost-savings but avert fewer cases of chronic disease. Results are sensitive to the intake elasticities of SSBs and produce.
	Limitations	Limitations of the study include a lack of information on heterogeneity in the SNAP-using population's response to price changes. There may also be potential recall biases in the data for the estimates of US food intake. Physical activity levels were assumed to remain stable. The study also did not consider location-specific data. The model also lacks direct purchasing data for SNAP participants. Predicting future price effects and understanding heterogeneities within the SNAP population are challenges. The focus on adult's limits insights into children, a substantial part of the SNAP recipient population.
	Funding source	None declared
	Conflicts of interest	None declared

Bemelmans et al.(18)		
8	Study aim	To estimate the cost-effectiveness of large-scale implementation of a community intervention and an intensive lifestyle program.
	Country	Netherlands
	Currency unit and year	EUR 2004
	Study design	Modelled CUA
	Setting	Community based intervention: General community<br>Intensive lifestyle program: Health care setting
	Intervention	Community based intervention for the general community (provided to 90% of population)<br>Intensive lifestyle program for overweight adults (a multicomponent weight loss program, offered to 10% of overweight adults)<br>Combined implementation of both interventions
	Comparator	No intervention
	Target population	Dutch adult population: community based intervention provided to 90% of population (n=11,940,000 people); intensive lifestyle program offered to 10% of overweight adults (n=650,000).
	Perspective	Health care system
	Time horizon	20 years and 80 years
	Model specification (if applicable)	The CDM, a dynamic Markov-type multi state transition model, was used to project future gains in life-years and QALYs in the population.
	Measurement and valuation of outcomes	Effectiveness of community intervention sourced from one study from the literature (BMI decrease).<br>Effectiveness of intensive lifestyle program sourced from one study from the literature (BMI decrease).<br><br>Effects for those who would be exposed to both interventions were assumed to be additive for the combined intervention scenario.<br><br>Changes in prevalence of overweight and obesity and physical inactivity.

		Cost-effectiveness was assessed in terms of costs per life year and (QALY) saved, arising from cases of disease prevented.
	Measurement and valuation of resources and cost	The costs of the interventions were based on the two Dutch projects from which effectiveness estimates were taken. The costs from the studies were multiplied to approximate for the population at scale. Impacts of assumed scale were not taken into account explicitly in this costing.
	Discount rate	4%
	Methods for uncertainty	Not stated
	Methods for sensitivity	Sensitivity analyses were performed for variations in costs (+20%/-20%), effects, time horizon (20 and 80 years), and discount rates (none, 4% costs, 1.5% effects).<br><br>Variations in effects from a permanent decrease in overweight of one percentage point and no effect on physical inactivity ('worst case') to a permanent decrease in prevalence rate of both overweight and inactivity of four percentage points ('best case').
	Brief summary of results (incl. sensitivity results)	Community intervention €5100per life-year, €5000 per QALY gained. Intensive lifestyle intervention €8400per life-year, €7400 per QALY gained. Combined implementation of the interventions reduced prevalence of overweight by approximately 3 percentage points and of physical inactivity by 2 percentage points after 5 years, at a cost of 7 euros per adult capita per year. The cost-effectiveness ratio of combined implementation amounts to €6000 per life-year gained and €5700 per QALY gained. Results are robust to sensitivity analyses.
	Limitations	Limitations include that no consideration that the rates of overweight may increase over time and this could offset the effects of the interventions. Estimates are based on assumptions, do not incorporate

		potential spillover effects and only consider the implementation costs of interventions.
	Funding source	This research was performed on behalf of the Dutch Ministry of Health.
	Conflicts of interest	None declared
**Brown et al.(7)**		
9	Study aim	To conduct a review of the literature and estimate the obesity, injury and physical activity related health impacts of a fuel excise taxation intervention for the 2010 Australian population.
	Country	Australia
	Currency unit and year	AUD 2010
	Study design	Modelled CUA
	Setting	Policy
	Intervention	Hypothetical intervention of AUD0.10 per litre increase to the national fuel excise tax in Australia.
	Comparator	No intervention
	Target population	2010 Australian working age population (aged 18 to 64 years), moderately or highly physically active persons
	Perspective	Limited societal
	Time horizon	Rest of life or 100 years
	Model specification (if applicable)	Proportional multi-state, multiple cohort life table model estimated obesity and PA-related health outcomes for the 2010 Australian population. Injury effects were also incorporated.
	Measurement and valuation of outcomes	Scoping review of the literature to inform parameters for cost-effectiveness modelling undertaken. Scoping review of published associations between obesity, physical activity, walking or cycling and fuel price or taxation; and cross-price elasticities of public transport use. Increase in taxation – Modal shift to public transport from car use estimated using conservative cross-price elasticity values - Increase in time spent in PA (walking to/from public transport) in moderately or highly physically active people- MET minutes per week - HALYs

	Measurement and valuation of resources and cost	Assumed minimal intervention costs as taxation of fuel already exists. Costs included legislative change costs. Vehicle operating cost savings were presented separately.<br><br>Healthcare costs of diseases averted using published data.
	Discount rate	3%
	Methods for uncertainty	Uncertainty analysis around the relative risk of incident disease and key input parameters estimated by Monte Carlo simulation using the Excel add-in Ersatz (version 1.34).
	Methods for sensitivity	One-way sensitivity analyses (two higher cross price elasticities from the literature; assuming longer distance walked to public transport), and "plausible case" scenario (using higher but still credible values for cross price elasticity, fuel price, prevalence of commuting using public transport, MET value, distance to public transport) as compared to base case analysis). Also modelled obesity-related effects only.
	Brief summary of results (incl. sensitivity results)	The intervention would be cost-effective over the lifetime, with a median ICER of 7702 (95% UI 1366–22,125). Cost-effectiveness results are very sensitive to the choice of price elasticity estimate. Under "plausible case" scenario, the intervention would be dominant.
	Limitations	Assumptions needed to model to scenarios. Assumed maintenance of intervention effect over the lifetime. Transport data limited in Australia, and so population limited to working age persons who are already physically active.
	Funding source	National Health and Medical Research Council funded Centre of Research Excellence on Obesity Policy and Food Systems (Grant no. 1041020).
	Conflicts of interest	None declared
**Brown et al.(10)**		
10	Study aim	To estimate the cost-effectiveness of legislation to restrict food and beverage HFSS TV advertising until 9:30 pm, and to examine how health benefits and healthcare cost-savings differ by SEP.

	Country	Australia
	Currency unit and year	AUD 2010
	Study design	Modelled CUA
	Setting	Policy
	Intervention	Defined as legislation to implement time-based restrictions of unhealthy food and beverage marketing to children under 16 years of age on free to air TV until 9:30 pm.
	Comparator	No intervention
	Target population	Australian children aged 5-15 years
	Perspective	Limited societal perspective
	Time horizon	Rest of life or 100 years
	Model specification (if applicable)	A proportional multi-state, multiple cohort life table model was used to estimate obesity-related health outcomes and healthcare cost-savings from intervention. Modelling was undertaken for a whole population analysis, and an analysis by area-level SEP, using the SEIFA IRSD quintiles 1 (most disadvantaged) and 5 (least disadvantaged).
	Measurement and valuation of outcomes	Logic pathway modelling, based on a scoping search of the literature for the effects of TV advertising on consumption in children and adolescents. Authors conducted a meta-analysis of relevant experimental studies, and used estimate to model to kcal effect per minute exposed to TV ads.<br>Applied a crude adjustment factor of 50% of effect estimates reported in experimental studies would translate and be maintained in a real-world setting.<br>Kcal effect per minute exposed to TV ads – time spent watching ads- change in mean energy intake- change in mean BMI.
	Measurement and valuation of resources and cost	Fixed costs: cost of passing legislation, sourced from the literature.<br>Ongoing costs: salary of two administration and compliance officers, based on assumptions and market rates.<br><br>Healthcare cost-savings from diseases averted from published data.

	Discount rate	3%
	Methods for uncertainty	Uncertainty analysis around key input parameters was estimated using Monte Carlo simulation (2000 iterations) using the Excel add-in Ersatz (version 1.35) to estimate 95% uncertainty intervals (95% UI).
	Methods for sensitivity	Sensitivity analyses included potential loss of revenue to TV networks resulting from reduced advertising (1 year loss of revenue). Multivariate "worst case" analysis incorporated smaller effect estimate derived from the meta-analysis, revenue loss and varied the assumption that all TV viewing was on free to air TV. A threshold analysis was also undertaken to estimate the minimum effect size required for the intervention to be considered cost-effective using a AUD50,000 per HALY threshold.
	Brief summary of results (incl. sensitivity results)	The cost-effectiveness modelling showed that the intervention is highly effective with a total cost-savings of AUD777.9M (95% UI AUD369.8M–AUD1.2B) at the population level over the lifetime.<br><br>The intervention resulted in 1.5 more HALYs and 1.4 times higher cost-savings for children residing in the most disadvantaged areas compared to the least disadvantaged areas.<br><br>Worst-case sensitivity analysis with conservative input parameters suggested that the intervention would remain dominant.<br><br>Threshold analysis demonstrated that the effect size, expressed as the relationship between 'minutes of exposure to TV ads for HFSS food' and 'energy intake', could be reduced by more than two orders of magnitude.
	Limitations	The intervention population was limited to children aged five to 15 years. Other limitations include assumptions about the maintenance of BMI effects into adulthood, reliance on a meta-analysis of studies in highly controlled settings and assumptions re share of free to air TV viewership. It also did not consider the possibility of advertising shifting from TV to other media in response to the intervention.

	Funding source	National Health and Medical Research Council funded Centre for Research Excellence in Obesity Policy and Food Systems (grant no. 1041020).
	Conflicts of interest	None declared
**Burn et al.(19)**		
11	Study aim	To determine the cost-effectiveness of the MobileMums intervention
	Country	Australia
	Currency unit and year	AUD 2014
	Study design	Modelled CUA
	Setting	eHealth
	Intervention	MobileMums is a 12-week programme which assists mothers with young children to be more physically active, primarily through the use of personalised SMS text-messages.
	Comparator	No intervention
	Target population	Hypothetically offering the intervention to all women in Queensland, Australia with children under 1 year old. Assuming an uptake of 60% (based on the RCT), this equates to 36 364 women participating.
	Perspective	Health system perspective
	Time horizon	2 years
	Model specification (if applicable)	State-based Markov model, with two states: physically inactive, physically active and monthly cycles. Spending a month as active or inactive has a cost and health outcome associated.
	Measurement and valuation of outcomes	Efficacy findings from the RCT. Health effects are expressed in QALYs, using utility values from participants in the RCT to estimate impacts on HRQoL.
	Measurement and valuation of resources and cost	Extrapolation of costs from the RCT, where assumptions regarding the number of behavioural counsellors and program coordinators required for widespread dissemination were made. Assumptions regarding the number of participants that could be assigned to counsellors, and number of counsellors overseen by coordinators. Assumed salary rate (health

		practitioners, 2 years experience). Costs of computer programming and text messages as per the trial.<br><br>Healthcare cost-savings using data on health service utilisation from the RCT.
	Discount rate	5 %
	Methods for uncertainty	Parameter uncertainty was quantified using Monte Carlo simulations, with the model evaluated 10 000 times, with each simulation involving random draws from each parameter distribution.
	Methods for sensitivity	Sensitivity analyses included: 1) all program activity effects are mitigated entirely after T3, the estimated treatment effect observed at T3 is maintained for a further 15 months, at which point the treatment effect is entirely mitigated 2) number of counsellors and coordinators required is increased by 50% and reduced by 50%, and 3) increasing the cohort size by 50% and reducing it by 50%.
	Brief summary of results (incl. sensitivity results)	MobileMums has a 98% probability of being cost-effective at a cost-effectiveness threshold of 64 000 AUD. Varying modelling assumptions has little effect on this result.
	Limitations	The model has a short time horizon, potentially causing underestimation of long term benefits and improvements in level of physical activity. It also only accounted for changes significant enough to transit a participant between the two states of the model and overlooked other changes that may occur within a state.
	Funding source	National Health and Medical Research Council project grant number 614244
	Conflicts of interest	None declared
**Cecchini et al.(20)**		
12	Study aim	To assess public health strategies for chronic diseases that are closely linked with obesity, including aspects of diet and physical inactivity, in Brazil, China, India, Mexico, Russia, and South Africa. England was included for comparative purposes.

	Country	Brazil, China, India, Mexico, Russia, and South Africa, England
	Currency unit and year	USD 2005
	Study design	Modelled CUA
	Setting	Cross- and multi-sectoral, including workplaces, policy, schools
	Intervention	The interventions assessed were school-based health promotion interventions, worksite health promotion interventions, mass media health promotion campaigns, counselling of individuals at risk in primary care, fiscal measures affecting the prices of fruit and vegetables and foods high in fat, regulation of food advertising to children, and compulsory food labelling. A combined strategy (a mass media campaign, fiscal measures, food advertising regulation, and food labelling) was also estimated, assuming the effects of the individual interventions, measured in terms of relative risks of risk factors or chronic diseases, would combine multiplicatively.
	Comparator	No intervention
	Target population	Country-specific information was used to establish potential population coverage for each intervention.<br>Food labelling (100% of population)<br>Food advertising regulation: Impact of regulation on children’s exposure to food advertising based on 1 evaluation from the literature (19.3-36.5% of population)<br>Physician counselling (1.1-14.7% of population)<br>Fiscal measures (100% of population)<br>Mass media campaigns (61.1-80.4% of population)<br>Worksite interventions (3.4-15.7% of population)<br>School-based intervention (1.7-4.2% of population)
	Perspective	Health/funder perspective
	Time horizon	Lifetime (100 years)

	Model specification (if applicable)	OECD/WHO microsimulation chronic disease prevention (CDP) model, applies the effects of an intervention on risk factors to the relevant target age groups, taking into account the likely coverage of the same age groups. Effects then progressively affect more proximal risk factors, older age groups, and new birth cohorts as the simulation develops.
	Measurement and valuation of outcomes	Intervention specific<br>Individual-level effectiveness is based mostly on studies from high-income settings. Country-specific information was used to establish potential population coverage and to adapt effectiveness to the local population distribution of risk factors.<br><br>Fibre consumption (g per day), fat (% total energy), % of people who are physically active, BMI, cholesterol and SBP – DALYs<br>Individual-level effectiveness is based mostly on studies from high-income settings.<br>Food labelling: Based on evidence in two studies from the literature<br>Food advertising regulation: Effect of advertising on BMI based on 1 study from the literature<br>Physician counselling: Effect based on three studies of controlled experiments<br>Fiscal measures: Changes in consumption of fat and F&V based on conservative estimates of price elasticity of demand, from the literature<br>Mass media campaigns: Based on evidence in three studies from the literature<br>Worksite interventions: Based on evidence in three studies from the literature<br>School-based intervention: Based on evidence in four studies from the literature

	Measurement and valuation of resources and cost	Standardised approach to intervention costing, using information about the quantities of physical inputs needed and unit costs.<br><br>Healthcare cost-savings from cases of disease averted using published data.<br><br>Food labelling- Costs related to administration, planning, enforcement, preparation and distribution of posters, food inspection<br><br>Food advertising regulation: Costs related to administration, planning, monitoring and enforcement, minor training for staff overseeing implementation.<br><br>Physician counselling: Extra labour time, assuming that target individuals spend on average 25 minutes over 2.6 sessions with their physician; laboratory costs, training of health professionals and basic organisation costs<br><br>Fiscal measures: Costs related to administatrion, planning, monitoring, and enforcement.<br><br>Mass media campaigns: Advertisements on radio and TV, flyers and leaflets; personnel costs to design, run and supervise the program; planning and administration.<br><br>Worksite interventions: Organisation and training of staff; seminar organisation; nutritionist fees; information materials, and guest speaker fee.<br><br>School-based intervention: Program organisation, training of teachers and staff, extra teaching time and additional curricular activities including books, and equipment.
	Discount rate	3%
	Methods for uncertainty	Probabilistic uncertainty analysis presented as cost-effectiveness planes in web appendix.

	Methods for sensitivity	Limited details given
	Brief summary of results (incl. sensitivity results)	Several interventions cost-effective – including health information and communication strategies that improve population awareness about the benefits of healthy eating and physical activity; fiscal measures that increase the price of unhealthy food content or reduce the cost of healthy foods rich in fibre; and regulatory measures that improve nutritional information or restrict the marketing of unhealthy foods to children. Combined intervention would deliver substantial health gains, with a very favourable cost-effectiveness profile.
	Limitations	Modelled representation, constrained by data availability to inform analyses.
	Funding source	Not stated
	Conflicts of interest	None declared
**Cobiac et al.(21)**		
13	Study aim	To evaluate the cost-effectiveness of six interventions to promote physical activity.
	Country	Australia
	Currency unit and year	AUD 2003
	Study design	Modelled CUA
	Setting	Dependent on intervention, included general practice, ehealth, and media.

	Intervention	GP prescription: Inactive patients identified by the GP receive a physical activity prescription from the GP and follow-up phone call(s) from an exercise physiologist.<br>GP referral to exercise physiologist: Screening questionnaires mailed to all patients and inactive patients invited to attend a series of counselling sessions with an exercise physiologist<br>Mass media-based six week campaign: Combination of physical activity promotion via mass media (television, radio, newspaper, etc.), distribution of promotional materials, and community events and activities.<br>TravelSmart: Targets households with tailored information (maps of walking paths and bus timetables) and provides merchandise as an incentive to reduce using cars for transport<br>Pedometers: Use of pedometers as a motivational tool to increase physical activity.<br>Internet: Physical activity information and advice across the internet via a web site and/or email.
	Comparator	No intervention, current practice
	Target population	GP prescription: 25% of sedentary and 10% of insufficiently active population aged 40–79 years. Target group derived from RCT recruitment rates and Australian GP statistics.<br>GP referral: 8% of sedentary and 3% of insufficiently active population aged 60+ years. Target group derived from RCT recruitment rates and Australian GP statistics.<br>Mass media: 100% of population aged 25–60 years. Target population based on population in quasi-experimental study of Australian campaign.<br>Internet: 2% of population (internet users) aged 15+ years. Target group derived from participation and attrition rates in 3 RCTs and Australian internet access statistics.

		Pedometers: 13% of population aged 15+ years. Target group derived from participation rates observed in similar program.<br>TravelSmart: 57% of population (urban) aged 15+ years. Target population derived from household contact rates in 21 TravelSmart studies.
	Perspective	Australian health sector perspective
	Time horizon	Lifetime
	Model specification (if applicable)	A multi-state, multiple cohort life- table approach to determine changes in mortality and morbidity for five physical activity-related diseases: ischaemic heart disease, ischaemic stroke, type 2 diabetes, breast cancer, and colon cancer.
	Measurement and valuation of outcomes	GP prescription: Effect derived from change in kcal/kg/week observed in RCT.<br>GP referral: Effect derived from change in sessions/week and change in min/session observed in RCT.<br>Mass media: Effect derived from change in h/week observed in the Australian campaign.<br>Internet: Effect derived from meta-analysis of change in MET-min/week in 3 RCTs.<br>Pedometers: Effect derived from change in steps/day from meta-analysis of 8 RCTs.<br>TravelSmart: Effect derived as weighted average of change in trips/week (walking/cycling) observed in the TravelSmart studies.<br><br>Intervention effect: DALYs<br>Assumed that the intervention effects on physical activity are sustained for the first year, but decay exponentially at a rate of 50% per annum thereafter.
	Measurement and valuation of resources and cost	GP prescription: Costs adapted from New Zealand study.

		GP referral: Total costs estimated from resource use (e.g., screening questionnaire printing/delivery, exercise physiologist salary, etc.) and assumptions (e.g. number of project officers).<br><br>Mass media: Costs estimated from similar Australian campaign. Costs were scaled up, where relevant, to a magnitude associated with a six-week campaign targeting 9.74 million Australia-wide (rather than a four-week campaign targeting 1.4 million in New South the similar campaign), then summed to determine the total cost of a mass media campaign intervention in Australia.<br><br>Internet: Costs estimated from costs for operating similar health Web site in Victoria.<br><br>Pedometers: Costs derived from weighted average of resource use in the 8 RCTs and costs of similar program.<br>TravelSmart: Costs derived from costs of TravelSmart intervention delivery in Western Australia.<br><br>Healthcare cost-savings from cases of disease averted using published data.
	Discount rate	3%
	Methods for uncertainty	Ninety-five percent uncertainty intervals are determined for all outcome measures by Monte Carlo simulation (2,000 iterations), using the Excel add-in tool @RISK (Palisade, Version 4.5). Uncertainty distributions around input parameters.
	Methods for sensitivity	Sensitivity is evaluated by varying decay rates between 0% (lifelong behaviour change) and 100% (behaviour change reversed after the first year).
	Brief summary of results (incl. sensitivity results)	Pedometers and mass media campaigns are the most cost-effective strategies, and likely to be cost-saving. Internet program, GP PA prescription and TravelSmart have a high probability of cost-effectiveness. GP referral is the least cost-effective intervention.

		Interventions become less cost-effective the faster the effects decay.
	Limitations	Generalisability of results may be challenging as outcome measures were inconsistent. Limited evidence for some interventions, including some issues with quality and level of evidence of effectiveness. Challenges in modelling PA, due to limited evidence base and reliance on observational studies. Possible time lag between change in risk and health benefits not accounted for.
	Funding source	Australian National Health and Medical Research Council Health Services Research grant (no. 351558)
	Conflicts of interest	None declared
**Cobiac et al.(22)**		
14	Study aim	To evaluate the cost-effectiveness of 23 interventions to promote fruit and vegetable consumption.
	Country	Australia
	Currency unit and year	AUD 2003
	Study design	Modelled CUA
	Setting	Varied per intervention, included retail, telehealth, community, and health care
	Intervention	General population interventions (n=8): telephone counselling and information mailout, individual dietary counselling, individual and group dietary counselling, information mailout (tailored), information mailout (multiple tailored), information mailout (multiple re-tailored), phone counselling and information mailout, community-based events, sponsorship and promotion.<br><br>Supermarket (n=1): supermarket displays, flyers, and promotional materials<br><br>Worksite interventions (n=7): information seminars and promotional materials and cafeteria changes.

		Health care setting interventions(n=3): telephone counselling and information mailout, dietary counselling and telephone follow-up, dietary counselling and information mailout.<br><br>Low income (n=4): telephone counselling and information mailout, farmers market vouchers, supermarket vouchers, peer counselling, telephone counselling and promotional materials.
	Comparator	No intervention
	Target population	Adult populations, dependent on intervention, varied from adults aged 18+ years to specific adult age groups (e.g. 18 to 65 years).
	Perspective	Australian health sector perspective
	Time horizon	Lifetime
	Model specification (if applicable)	Proportional lifetable model. Diseases included ischaemic heart disease and stroke, and colon, lung, stomach and oesophageal cancers.
	Measurement and valuation of outcomes	Effect estimates taken from primary studies (serves/day) – DALYs<br><br>Assumption of an exponential decay in effect at the rate of 50% per year after the end of the intervention.
	Measurement and valuation of resources and cost	Components of each intervention were identified from published studies, and used to estimate cost per participant if applied in the Australian population.<br><br>Healthcare cost-savings from cases of disease averted using published data.
	Discount rate	3%
	Methods for uncertainty	Monte Carlo analysis (@Risk; Palisade, Version 4.5) to derive 95% uncertainty intervals for all outcome measures and to determine probabilities of intervention cost-effectiveness
	Methods for sensitivity	Varying effect decay rates between 0% and 100%. Sensitivity of the results to the exclusion of future trends in disease incidence and case

		fatality. The impact of adjustments to the serving size estimate (halving or doubling the 80g estimate) in the intervention trials.
	Brief summary of results (incl. sensitivity results)	Interventions that used dietary counselling, telephone contact, worksite promotion or other methods to encourage change in dietary behaviour were not highly effective or cost-effective.<br>Out of 23 interventions, only 5 were considered cost-effective at less than $50,000 per DALY. No interventions assuming 100% decay and 17 interventions were cost effective if effect maintained.
	Limitations	Lack of data on the sustainability of behavioural changes. No substitution effects incorporated. Changes in fat intake not incorporated.
	Funding source	National Health and Medical Research Council (351558).
	Conflicts of interest	None declared
**Cobiac et al.(23)**		
15	Study aim	To evaluate a range of food and drink taxes and subsidies, implemented individually and in all combinations, to determine an optimally cost-effective package of tax and subsidy options. Relevant interventions included: 1) taxing saturated fat, 2) taxing excess salt in processed foods, 3) taxing sugar-sweetened beverages, 4) subsidising fruits and vegetables, and 5) taxing processed foods high in sugar.
	Country	Australia
	Currency unit and year	AUD 2010
	Study design	Modelled CUA
	Setting	Policy
	Intervention	Relevant here: Saturated fat tax, sugar-sweetened beverage tax, fruit and vegetable subsidy, sugar tax
	Comparator	No intervention
	Target population	Australian population 2010
	Perspective	Health sector perspective
	Time horizon	Lifetime
	Model specification (if applicable)	Proportional multi-state lifetable modelling

	Measurement and valuation of outcomes	Change in price of relevant Australian food and drink products – change in consumption using price elasticity data - DALYs
	Measurement and valuation of resources and cost	Cost of implementing new tax or subsidy based on previous published estimated of cost.<br><br>Healthcare cost-savings from cases of disease averted using published data.
	Discount rate	3%
	Methods for uncertainty	Monte Carlo simulation (2,000 iterations) to determine 95% uncertainty intervals.
	Methods for sensitivity	Possible demand- and supply-side responses to the imposition of food taxes and subsidies in scenario analyses, including feasibility constraints on changes in total energy intake and total weight of foods consumed, food industry reformulation of foods to avoid taxes, and under- or over-shifting of price changes on taxed products.
	Brief summary of results (incl. sensitivity results)	A combination of taxes and subsidy might avert as many as 470,000 DALYs (95% uncertainty interval [UI]: 420,000 to 510,000) in the Australian population of 22 million, with a net cost- saving of AU$3.4 billion (95% UI: AU$2.4 billion to AU$4.6 billion; US$2.3 billion) to the health sector.<br>Sugar tax produced the biggest estimates of health gain (270,000 [95% UI: 250,000 to 290,000] DALYs averted), followed by the salt tax (130,000 [95% UI: 120,000 to 140,000] DALYs), the saturated fat tax (97,000 [95% UI: 77,000 to 120,000] DALYs), and the sugar-sweetened beverage tax (12,000 [95% UI: 2,100 to 21,000] DALYs).
	Limitations	Results have used price elasticities derived from New Zealand. The magnitude of health benefits is sensitive to measures of price elasticity. Potential residual confounding from missing or poorly measured explanatory variables in the observational studies the analyses was based on. Random error or misclassification in exposure measurements would also lead to an underestimation of the strength of an association.

	Funding source	National Health and Medical Research Council Fellowship (Grant number 1036771)
	Conflicts of interest	LJC is a member of the Editorial Board of PLOS Medicine.
**Cradock et al.(24)**		
16	Study aim	To estimate the cost-effectiveness of six strategies to increase physical activity in US school, afterschool and childcare settings.
	Country	United States
	Currency unit and year	USD 2014
	Study design	Modelled CEA
	Setting	US school, afterschool and childcare settings
	Intervention	Six interventions: active physical education, active recess, active school day, healthy afterschool, new afterschool programs, and hip hop to health
	Comparator	No intervention
	Target population	Active PE: Children in grades kindergarten through 8 (ages 5-14) in public elementary and middle schools in states without an active PE policy, assuming 96% of students attend PE classes regularly and 70% of trained teachers implement the policy.<br><br>Active recess: Children in grades kindergarten through 5 (ages 5-12) who attend public elementary schools not already implementing active recess strategies (95% of schools) that choose to newly adopt the active recess program (assumed 50% of eligible schools would adopt the program).<br><br>Active school day: Children in grades kindergarten through 8 (ages 5-15) who attend public elementary and middle schools in school districts without an active school day policy. Estimate that 88% of elementary and 91% of middle school students attend in districts without a policy, and assume 100% of eligible districts will adopt the policy.<br><br>Healthy afterschool: Children and adolescents (ages 5-11, grades K-5) in 21st Century Learning or state-funded afterschool programs in states that

		do not have existing guidelines for nutrition or physical activity. Assumed that 20% of programs voluntarily agree to participate in the recognition program.<br><br>New afterschool: Low-income elementary school-age children (ages 5-12) not now participating in afterschool programs, but who would if programs were available using estimates from published study.<br><br>Hip hop to health, Jr. Ages 3–5: All 3-5 year old children attending licensed ECE programs (both centers and family daycare homes) in the U.S. Assumed all states adopt the policy, and 73% of eligible ECE programs complete the intervention.
	Perspective	Modified societal perspective
	Time horizon	Ten years from 2015-2025
	Model specification (if applicable)	Individual level microsimulation model of the population in the United States.
	Measurement and valuation of outcomes	Active PE: logic modelling and evidence from one meta-analysis. Increase in MVPA.<br><br>Active recess: logic modelling and evidence from published studies. Increase in MVPA.<br><br>Active school day: logic modelling and evidence from one study. Increase in MVPA.<br><br>Healthy afterschool: logic modelling and evidence from two studies. Reduction in kcal intake and increase in VPA.<br><br>New afterschool: logic modelling and evidence from four studies. Reduction in kcal intake and increase in MVPA.

		Hip hop to health, Jr: logic modelling and evidence from two studies. Reduction in kcal intake and increase in MVPA.<br><br>Changes in VPA/MVPA and/or kcal intake – BMI
	Measurement and valuation of resources and cost	Active PE: Based on models of active PE programs and reasonable assumptions re scale to a state level (additional state level co-ordination)<br><br>Active recess: Assumed intervention resources and unit costed, based on published studies.<br><br>Active school day: Assumed intervention resources and unit costed.<br><br>Healthy afterschool: Assumed intervention resources and unit costed.<br><br>New afterschool: Estimated based on resources described by the authors of the four studies, plus a published CEA.<br><br>Hip hop to health, Jr: Estimated based on resources and unit costed, with some estimates provided by program staff.<br><br>Health care cost reductions due to changes in obesity cases
	Discount rate	3%
	Methods for uncertainty	Probabilistic sensitivity analysis to account for uncertainty in underlying model inputs to calculate 95% UI using 1000 Monte Carlo iterations for a simulated population of one million individuals scaled to the national population.
	Methods for sensitivity	Not stated
	Brief summary of results (incl. sensitivity results)	All of the six interventions were estimated to increase physical activity levels among children and adolescents in the US population and could prevent approximately 2500 to 110,000 cases of childhood obesity depending on the intervention implemented.

	Limitations	Broader health benefits of PA not captured by the model. The conservative approach of the study does not also fully account for potential cost offsets related to the overall health impact of physical activity beyond obesity and focused solely on the health care costs attributed to obesity within a ten-year modelling framework.
	Funding source	The JPB Foundation, the Robert Wood Johnson Foundation (Grant No. 66284), the Donald and Sue Pritzker Nutrition and Fitness Initiative, and the Centers for Disease Control and Prevention (Grant No. U48/DP001946).
	Conflicts of interest	None declared
**Crino et al.(11)**		
17	Study aim	To estimate the potential cost-effectiveness of: 1) a package size cap on single-serve SSBs >375 mL (package size cap), and 2) product reformulation to reduce energy content of packaged SSBs (energy reduction).
	Country	Australia
	Currency unit and year	AUD 2010
	Study design	Modelled CUA
	Setting	Policy
	Intervention	A package size cap of 375 mL on packaged single-serve SSBs sold in Australia and, product reformulation to reduce energy content of packaged SSBs (energy reduction; 5% kJ reduction and 30% kJ reduction).
	Comparator	Compared to base case of government imposed legislation of 1) banning sales of single-serve, packages SSBs greater than 375ml, and 2) reduced kj/serve by 5% for all SSBs.
	Target population	2010 Australian population
	Perspective	Limited societal perspective
	Time horizon	Lifetime
	Model specification (if applicable)	ACE model (ACE-Obesity project), a multi-state, multiple cohort life table model

	Measurement and valuation of outcomes	Logic modelling. Effect sizes estimated using published data and assumptions.<br><br>Decreased consumption/reduced energy density-reduction in energy intake-body weight-HALYs
	Measurement and valuation of resources and cost	Cost to government for passing legislation from the literature; education and monitoring costs; implementation and upkeep costs. Costs to NGOs for advocacy, marketing and promotion of interventions based on real world example of labelling change. Cost to industry for packing changes and implementation costs based on reports prepared for a real world example of labelling change.<br><br>Healthcare cost-savings from cases of disease averted using published data.
	Discount rate	3%
	Methods for uncertainty	Monte Carlo simulation (2000 iterations) was used to estimate parameter uncertainty using Ersatz (version 1.35) software.
	Methods for sensitivity	Scenarios were modelled (e.g including both mandatory and voluntary implementation; incorporating compensatory intake; incorporating substitution).
	Brief summary of results (incl. sensitivity results)	Total estimated intervention costs were AUD 210 million. Both interventions resulted in reduced mean body weight (package size cap: 0.12 kg; energy reduction: 0.23 kg); and HALYs gained (package size cap: 73,883; energy reduction: 144,621). Cost offsets were estimated at AUD 750.8 million (package size cap) and AUD 1.4 billion (energy reduction). Cost-effectiveness analyses showed that both interventions (in all scenarios) were dominant.
	Limitations	Direct evidence of intervention effect is weak. Data limitations re availability of consumption by SSB package size, loss of revenue to the food industry was not included.

	Funding source	National Health and Medical Research Council, Centre for Research Excellence in Obesity Policy and Food Systems (APP1041020).
	Conflicts of interest	M.C. interacts regularly on a non-financial basis with multiple large corporations in the Food Processing Industry and the Quick Service Restaurant industry in Australia and overseas as a part of her work to improve the quality of the food supply. All other authors declare no conflict of interest.
**Dallongeville et al.(25)**		
18	Study aim	To quantify the cost-effectiveness of three policies aimed at increasing F&V consumption: (i) reduction of the consumer price through a decrease in VAT on all F&V, (ii) consumption subsidies through F&V stamps and (iii) generic information campaigns.
	Country	France
	Currency unit and year	EUR 2006
	Study design	Modelled CUA
	Setting	Policy/education
	Intervention	(i) Reduction of the consumer price through a decrease in VAT on all F&V, (ii) consumption subsidies through F&V stamps and (iii) generic information campaigns.
	Comparator	Not clearly stated
	Target population	French population
	Perspective	Not clearly stated
	Time horizon	Not clearly stated
	Model specification (if applicable)	Economic model, using Monte–Carlo simulations
	Measurement and valuation of outcomes	Changes in F&V consumption using elasticities (price, income, supply; using evidence of effect of advertising for information campaigns) from the literature- change in diseases related to F&V consumption/mortality-LYS. Consumption from published sources, elasticities from the literature.
	Measurement and valuation of resources and cost	Limited detail given. Cost of a decrease in VAT: €465 M and corresponds to the loss of tax revenues due to the VAT reduction. Cost

		of F&V subsidy: assumed that €465 M were used to subsidize F&V consumption. Cost of education campaign: assumed a €10 M information campaign budget, which corresponds to the annual amount spent by public authorities and producers' associations to promote F&V consumption in France.
	Discount rate	Not stated
	Methods for uncertainty	Monte Carlo simulation
	Methods for sensitivity	Pessimistic and optimistic scenarios presented for education campaign analysis.
	Brief summary of results (incl. sensitivity results)	LYS are larger with VAT reduction than F&V stamps policies. Information campaigns are the most cost-effective.
	Limitations	Lack of randomised intervention studies to inform input parameters.
	Funding source	Not stated
	Conflicts of interest	None declared
**Doring et al.(26)**		
19	Study aim	To assess the effects and costs of a population-based primary prevention intervention targeting pre-school children attending child health centres in Sweden.
	Country	Sweden
	Currency unit and year	EUR 2015
	Study design	Within-trial CEA
	Setting	Child health services
	Intervention	Nine sessions delivered by specially trained nurses, in a timeframe of approximately 39 months. The intervention aimed to assist first time parents in promoting healthy food and physical activity habits in their children and in changing their own health behaviors if needed through the application of motivational interviewing. The intervention was targeting eating pattern (i.e., regular meals together with the family, no force feeding/eating), food choices (i.e., consumption of fruit and vegetables, reduced consumption of soft drinks and snacks), and physical activity (i.e., incorporating physical

		activity in the everyday routine, reducing sedentary time).
	Comparator	Regular age-related health check-ups of Swedish child health services
	Target population	Pre-school children (9–48 months of age)
	Perspective	Societal
	Time horizon	Intervention period, up to age 4 years
	Model specification (if applicable)	NA
	Measurement and valuation of outcomes	BMI at age 4 years (measured by study nurses at each visit)<br><br>BMI at age 4 years not statistically significantly different between groups (-0.11, 95% CI -0.31 to 0.08)
	Measurement and valuation of resources and cost	Prospective data collection alongside the trial.<br><br>Intervention delivery (5-day workshop, nurse training, supervision of nurses, staff time), catering and materials, travel, and productivity costs (parents time).
	Analytics and assumptions	Costs and effects derived from participant level data. Total costs of intervention compared to costs of usual care. ICER estimated as cost per 1 BMI unit prevented. Analyses undertaken in Microsoft Excel.
	Discount rate	Not reported
	Methods for uncertainty	Non-parametric bootstrapping; cost-effectiveness acceptability curve.
	Methods for sensitivity	Intervention costs calculated as per-protocol. Instead of individual uptake and duration of meetings, assumed full uptake (seven face-to-face meetings and two telephone meetings) and the duration of meetings according to the manual specification. Missing information on parents' attendance was imputed based on the observed distribution of parents' attendances during respective meetings.<br><br>In a second scenario analysis, the observed duration of meetings was halved. This was to partly account for potential overlap with usual health care during the intervention meetings, but also to allow for a shorter

		duration of intervention meetings if implemented in the current CHC practices. The effect measure was kept constant.
	Brief summary of results (incl. sensitivity results)	The estimated additional mean total costs of the PRIMROSE intervention were 342 Euro (95% CI: 334; 348) per child. The incremental cost-effectiveness ratio in the base case analysis was 3,109 Euro per 1 BMI unit prevented.
	Limitations	Doesn't account for costs and consequences in the long run
	Funding source	Salary support from the Excellence Fellowship of the Olympia-Morata Habilitation Programme. The Swedish Research Council for Health, Working Life and Welfare (2006-0226 and 2011- 0413), the Swedish Research Council (K2006-27X-20069-01-3 and K2012-69X-22058-01-3), the Research and Development Committee, Stockholm County Council (2006-0324), the Regional Research Council of the Uppsala and Örebro Health Care Region (RFR-12404), Uppsala County Council, Sörmland County Council, the Public Health Committee of Stockholm County Council (0803-377), the Vårdal Foundation (B2007-006), AFA Insurance (H-06:05/070001), and the Foundation of the Swedish Diabetes Society (TMA2006-004)
	Conflicts of interest	None declared
**Ekwaru et al.(27)**		
20	Study aim	To conduct an economic evaluation of school-based obesity prevention interventions previously identified as feasible, acceptable, and sustainable in the Canadian context.
	Country	Canada
	Currency unit and year	CA2016
	Study design	Modelled threshold analyses – CUA – and ROI analyses
	Setting	Schools
	Intervention	Modelled interventions included a comprehensive school health approach, multicomponent interventions and modifications of the existing PE curriculum.
	Comparator	No intervention

	Target population	Not clearly stated
	Perspective	Not clearly stated
	Time horizon	84 years
	Model specification (if applicable)	Micro-simulation Markov model incorporating effects on risk factors, including inadequate vegetables consumption, inadequate fruit consumption, inadequate physical activity, and excess body weight.
	Measurement and valuation of outcomes	Effect sizes estimated by systematic review and meta-analysis.<br><br>The incremental effects were measured in terms of years with chronic disease prevented and QALY gained.
	Measurement and valuation of resources and cost	Healthcare costs of diseases averted using data from the Canadian Institute for Health Information.<br>Due to intervention cost information rarely presented in the literature, an estimated threshold for cost-effectiveness used was CA$50,000 per QALY gained.
	Discount rate	1.5%
	Methods for uncertainty	Probabilistic sensitivity analysis to simultaneously account for uncertainties in all model parameters, 20 million simulations in Python.
	Methods for sensitivity	Different discounting rates were used as part of the sensitivity analysis (1.0%, 2.0%, and 3.0%).
	Brief summary of results (incl. sensitivity results)	The estimated costs of interventions that were cost-effective per quality-adjusted life year gained were $682, $444, and $416 per student for comprehensive school health, multicomponent, and physical education curriculum modification programs, respectively. The comprehensive school health intervention had the highest return-on-investment where $100 per student would avoid $824 in future direct healthcare costs associated with chronic disease.
	Limitations	Did not have cost data for interventions. Results may be underestimated given not all risk factors included in the model. Spillover effects also not considered.

	Funding source	Alberta Innovates Collaborative Research and Innovative Opportunities Team grant (201300671).
	Conflicts of interest	None declared
**Frew et al.(28)**		
21	Study aim	To determine the cost-effectiveness of a physical activity program (Be Active) aimed at city- dwelling adults living in Birmingham, UK.
	Country	United Kingdom
	Currency unit and year	GBP 2009/10
	Study design	Modelled CUA
	Setting	Community
	Intervention	"'Be Active' – allows Birmingham city residents to access their local council-run leisure centres without charge at certain times of the day. Free access to fitness gyms (including induction sessions), swimming pools and group fitness classes during off-peak hours (until 17:00) on weekdays and limited hours (after 13:00) on weekends.
	Comparator	No intervention
	Target population	Birmingham adult population aged 16–70 years
	Perspective	Healthcare and a wider perspective
	Time horizon	5 years
	Model specification (if applicable)	Markov state-transition model adapted from previous literature.
	Measurement and valuation of outcomes	Pragmatic approach to effect estimate measurement – before and after approach measurement of physical activity levels using self-report primary data collection.<br><br>Physical activity converted to MET, modelled to QALYs using data collected via EQ5D.
	Measurement and valuation of resources and cost	The cost of the intervention was determined by dividing the total cost of setting up and running the scheme by the number of Be Active members, to obtain average per-participant annual costs. Changing annual usage rate of 50-100%. The annual health service cost associated with each of the disease states were obtained from the literature.

	Discount rate	3.5%
	Methods for uncertainty	Triangular distribution for costs.
	Methods for sensitivity	(1) varied the ‘sustainability’ of the intervention by imposing a reduction in physical activity after the first year following the intervention (assumed that half of the sample who had initially improved their physical activity levels in the first year (eg, moved from ‘active’ to ‘recommended activity’) had dropped back down into the active physical activity health state by the end of year 1). (2) Altered the time-horizon to 10 years and 2 years. (3) Removed the ‘start-up’ costs from the intervention.
	Brief summary of results (incl. sensitivity results)	Under base-case assumptions— the Be Active program increased quality-adjusted life expectancy by 0.06 years, at an expected discounted cost of £3552, and thus the cost-effectiveness of Be Active was £400 per QALY. When the start-up costs of the program are omitted, the cost-effectiveness is further improved to £16 per QALY.
	Limitations	Collection of physical data was based on self-report. Underlying model assumption that improved physical activity levels will be sustained to realise the long-term health effects.
	Funding source	Consultancy grant from NHS South Birmingham PCT paid to the University of Birmingham.
	Conflicts of interest	None declared
**Gao et al.(8)**		
22	Study aim	To assess the economic credentials of a workplace-delivered intervention to reduce sitting time among desk-based workers.
	Country	Australia
	Currency unit and year	AUD 2014
	Study design	Within-trial, extrapolated to modelled CUA
	Setting	Workplace
	Intervention	Multicomponent workplace-delivered intervention to reduce sitting time
	Comparator	No intervention

	Target population	231 desk-based workers, aged 24–65 years, across 14 worksites of one organisation.<br>Extrapolated to eligible Australian population (conservatively assumed that one fifth of the 45% of Australians who work in a sedentary occupation would take up the intervention).
	Perspective	Societal perspective
	Time horizon	Within-trial; Lifetime
	Model specification (if applicable)	Markov cohort simulation model
	Measurement and valuation of outcomes	Between-group differences in changes in sick leave, total workplace sitting time, BMI, HRQoL.<br>Changes in sedentary time – METs – BMI - HALYs
	Measurement and valuation of resources and cost	Pathway analysis to identify resource use.<br>Some labour costs assuming national delivery of the intervention (8 implementation officers).<br><br>Healthcare cost-savings from cases of disease averted.
	Discount rate	3%
	Methods for uncertainty	Ersatz software used to estimate uncertainty.
	Methods for sensitivity	Univariate sensitivity analyses included discount rate of 0% and 6%, and annual decay rate of intervention effect 0% (assuming that the intervention effect would be maintained for 20 years), 10%, 50% and 100%.
	Brief summary of results (incl. sensitivity results)	The incremental cost-efficacy ratios (ICER) ranged from AU$9.94 cost/minute reduction in workplace sitting time to AU$13.37/minute reduction in overall sitting time. CUA results of ICER of AU$34 443/LY and AU$28 703/HALY if the intervention effects were sustained for five-years. CEA results were sensitive to assumptions surrounding intervention-effect decay rate and discount rate.
	Limitations	Trial participants were recruited from a single organization. The long-term model only captured changes in BMI and physical activity.<br>Assumptions regarding decay rate.

	Funding source	National Health and Medical Research Council of Australia project grant (#1002706), project funding from the Victorian Health Promotion Foundation's Creating Healthy Workplaces program and, by the Victorian Government's Operational Infrastructure Support Program.
	Conflicts of interest	None declared
**Goryakin et al.(29)**		
23	Study aim	To assess the impact on health and healthcare expenditure of seven public health policies to promote exercise and physical activity against a business as usual scenario.
	Country	Italy
	Currency unit and year	EUR 2015
	Study design	Modelled CUA
	Setting	Communities, workplaces, media, primary care, schools
	Intervention	Promotion of active transport, workplace sedentary interventions, investments in sports and recreation, mass media campaigns, prescription of physical activity in primary care, school-based interventions and mobile apps
	Comparator	No intervention, business as usual
	Target population	Promotion of active transport: expanding access to public transportation to an additional 1% of the Italian population aged >5 years.<br><br>Workplace sedentary interventions: 5.9% of employed aged 18-65 years.<br><br>Investments in sports and recreation: 100% of population aged >18 years.<br><br>Mass media campaigns: 100% of population aged >18 years.<br><br>Prescription of physical activity in primary care: 2 6 . 4% o f those aged 50-75 years with at least 1 risk factor for NCDs.

		School-based interventions: 90% of population aged 8-18 years.<br><br>Mobile apps: 2.21% of Italian population aged 15-64 years.<br><br>Change in METS: DALYs
	Perspective	Health system perspective
	Time horizon	To 2050 (30 years)
	Model specification (if applicable)	OECD SPHeP-NCD microsimulation model
	Measurement and valuation of outcomes	Promotion of active transport: evidence of effectiveness from systematic review and meta-analysis.<br><br>Workplace sedentary interventions: evidence of effectiveness from meta-analysis.<br><br>Investments in sports and recreation: Based on one study from the literature.<br><br>Mass media campaigns: evidence of effectiveness from grey literature report.<br><br>Prescription of physical activity in primary care: evidence of effectiveness from systematic review and meta-analysis.<br><br>School-based interventions: evidence of effectiveness from meta-analysis.<br><br>Mobile apps: evidence of effectiveness from meta-analysis.
	Measurement and valuation of resources and cost	Intervention costs are estimated broadly based on the WHO-Choice methodology, but little information is given in this publication.
	Discount rate	3%
	Methods for uncertainty	Not included

	Methods for sensitivity	Not included
	Brief summary of results (incl. sensitivity results)	Public policies to promote exercise have the potential to improve population health and be cost-effective through reducing number of chronic disease incidence, and resulting in cumulative gains in thousands of DALYs per year
	Limitations	Does not include other potential benefits, e.g. productivity.
	Funding source	Ministry of Health
	Conflicts of interest	None declared
**Graziose et al.(30)**		
24	Study aim	To estimate the long-term cost-effectiveness of an obesity prevention nutrition education curriculum (Food, Health, & Choices) as delivered to all New York City fifth-grade public school students over 1 year.
	Country	United States
	Currency unit and year	USD 2012
	Study design	Modelled CUA
	Setting	Schools
	Intervention	Food, Health, & Choices- 24-lesson nutrition education curriculum delivered over the course of 1 school year.
	Comparator	No-nutrition education
	Target population	Hypothetical citywide implementation scenario, modelled cohort of grade five students
	Perspective	Societal perspective
	Time horizon	Lifetime
	Model specification (if applicable)	Progression model, diagram given
	Measurement and valuation of outcomes	Effectiveness was based on a cluster-randomized, controlled trial in 20 public schools. BMI-QALYs
	Measurement and valuation of resources and cost	Retrospective intervention costing, using trial records. Adjusted using information from interviews with 2 current New York City elementary school teachers and staff of a similar intervention.

		Healthcare cost savings from cases of obesity prevented
	Discount rate	3%
	Methods for uncertainty	Monte Carlo simulation 10,000 runs in @RISK
	Methods for sensitivity	Probabiliistic sensitivity analysis varied several parameters, including intervention effectiveness, QALY estimates and lifetime direct medical cost estimates.
	Brief summary of results (incl. sensitivity results)	The Food, Health, & Choices intervention was estimated to cost \$8,537,900 and save 1,599 QALYs and \$8,098,600 in direct medical costs. The intervention is cost-effective at \$275 per QALY, with sensitivity estimates up to \$6,029 per QALY.<br>Choices is predicted to be cost-effective at \$275/QALY (95% confidence interval, –\$2,576/QALY to \$2,084/QALY) with estimates up to \$6,029/QALY in sensitivity analyses.
	Limitations	Results were contingent on the effectiveness estimate obtained in the original trial. Assumed intervention had the same effect across all contexts and that subgroups received the same benefit. Did not account for comorbidities averted.
	Funding source	US Department of Agriculture, National Institutes for Food and Agriculture (Award No. 2010-85215-20661)
	Conflicts of interest	Not stated
**Gulliford et al.(31)**		
25	Study aim	To estimate the cost effectiveness of a universal strategy to promote physical activity in primary care.
	Country	UK
	Currency unit and year	GBP 2010
	Study design	Modelled CUA
	Setting	Primary care
	Intervention	Universal strategy to promote physical activity in primary care.
	Comparator	Standard care

	Target population	Data to populate the model taken from large cohort of participants from the general practice research database. 262,704 adults aged 30-100 years.
	Perspective	Health care service
	Time horizon	Lifetime
	Model specification (if applicable)	Markov model that included five long-term conditions (diabetes, coronary heart disease, stroke, colorectal cancer and depression). The intervention was assumed to modify only the incidence of disease in healthy participants At Risk.
	Measurement and valuation of outcomes	The intervention effect on physical activity was from a meta-analysis of randomised trials.<br>PA-risk of disease-QALYs
	Measurement and valuation of resources and cost	The cost of the intervention was modelled as a fixed cost per person year depending on their physical activity level. In those not sufficiently physically active the intervention cost was one family practice consultation per year. In those sufficiently active, the cost was 20% of one family practice consultation (estimated time to complete screening questions).<br>Healthcare cost savings
	Discount rate	3.5%
	Methods for uncertainty	Probabilistic, Mean costs, and the 95 % range, were obtained from the data for 2,000 simulations.
	Methods for sensitivity	Sensitivity analyses included varying the discount rate of 1.5% for QALYs, unit costs of intervention.
	Brief summary of results (incl. sensitivity results)	Net health benefits at a threshold of £30,000 per QALY were 3.2 (−11.1 to 16.9) QALYs per 1,000 participants with 5 years intervention (probability cost-effective 64.7 %) and 5.0 (−9.5 to 19.3) with 10 years intervention (probability cost-effective 72.4 %).
	Limitations	Effectiveness data included in meta-analysis included self-report measures of PA. Intervention effects did not vary in population subgroups. Model included only healthcare costs.
	Funding source	UK National Prevention Research Initiative

	Conflicts of interest	None declared
**Hayes et al.(32)**		
26	Study aim	To determine the costs and cost-effectiveness of an early childhood home visiting program delivered to families in socio-economically disadvantaged areas of Sydney, Australia during 2007-2010.
	Country	Australia
	Currency unit and year	AUD 2012
	Study design	Within-trial CEA
	Setting	Maternal child health: home, community
	Intervention	Eight home visits by specially trained community nurses (30-36 weeks gestational age; 1, 3, 5, 9, 12, 15, and 24 months after birth), including advice and education on feeding, nutrition and physical activity. Usual childhood nursing service, consisting of one home visit by a community nurse within a month of birth, plus clinic visits.
	Comparator	Usual childhood nursing service, consisting of one home visit by a community nurse within a month of birth, plus clinic visits. Home safety information mailouts.
	Target population	667 first time mothers and their infants, recruited from antenatal clinics in a socially and economically disadvantaged area of Sydney.
	Perspective	Healthcare funder
	Time horizon	Within-trial; up to age 2 years
	Model specification (if applicable)	NA
	Measurement and valuation of outcomes	Alongside trial. Anthropometric measures (BMI, BMIz)<br><br>Mean difference between intervention and control BMIz at 2 years not statistically significant (0.23, 95% CI -0.026 to 0.475).
	Measurement and valuation of resources and cost	Retrospective costing alongside the trial<br>Intervention delivery (staff time, vehicle purchase, vehicle running costs for home visits, costs of training community nurses, educational materials, and equipment costs of scales and portable stadiometers).<br>Healthcare utilisation using linked data (doctor and specialist visits,

		medicines, in- and out-patient hospital stays and emergency department admissions).
	Analytics and assumptions	CEA using Stata version 12.0. Costs and effects derived from patient-level data, and bootstrapped to estimate a distribution around costs and health outcomes. ICERS calculated as cost per BMI unit avoided and cost per 0.1 BMIz score reduction. Cost-effectiveness acceptability curve.
	Discount rate	5%
	Methods for uncertainty	Bootstrapping; cost-effectiveness acceptability curve
	Methods for sensitivity	More realistic scenario, reflective of delivering the program in a ‘real world’ setting and incorporating potential economies of scale. Nurse time for each home visit was the major contributor to the cost of the intervention, and in the trial included a 70-min consultation time, and 90 min for return travel and administration time. A more realistic travel time for home visits was assumed, based on actual travel distances/times of usual care community health nurses in an urban setting in NSW. In the scenario analysis, consultation time and the number of visits per child were unchanged, but round trip distance was reduced from 52 to 4 km and travel plus administration time reduced from 90 min to a more realistic 20 min per visit.
	Brief summary of results (incl. sensitivity results)	The cost of the HB intervention in the clinical trial over 2 years was $1309 per child (2012 $AUD). The incremental cost-effectiveness ratio was $4230 per unit BMI avoided and $631 per 0.1 reduction in BMIz. It was estimated that the program could be delivered in practice for $709 per child; with incremental cost-effectiveness ratios of $2697 per unit BMI avoided and $376 per 0.1 reduction in BMIz
	Limitations	Retrospective evaluation; no preference based quality of life data so CUA not undertaken. Health benefits accruing to others not directly targeted by the intervention (e.g. parents) not included. ICERs likely to be conservative.
	Funding source	National Health and Medical Research Council project grant 1003780; Capacity Building Grant in Health Economics 571372

	Conflicts of interest	None declared
**Huse et al.(9)**		
27	Study aim	This study assessed the potential cost-effectiveness of mandatory restrictions on price promotions for SSBs in Australia.
	Country	Australia
	Currency unit and year	AUD 2010
	Study design	Modelled CUA
	Setting	Legislation
	Intervention	Mandatory restrictions on price promotions for SSBs
	Comparator	No intervention
	Target population	Australian population 2010
	Perspective	Limited societal perspective
	Time horizon	Lifetime
	Model specification (if applicable)	ACE model (ACE-Obesity project), a multi-state, multiple cohort life table model
	Measurement and valuation of outcomes	Australian dietary consumption data, together with UK data on the SSB sales uplift associated with price promotions from a report in the grey literature, were used to estimate reductions in SSB purchases and consequent changes in body mass index following the intervention. BMI-HALYs
	Measurement and valuation of resources and cost	Intervention costs were limited to those accrued by government. Costs were estimated using evidence from the literature and assumptions.<br>Due to the lack of available data, it was not possible to accurately estimate the financial impact of the modelled policy on retailers or SSB manufacturers.<br>Healthcare cost-savings from diseases averted.
	Discount rate	3%
	Methods for uncertainty	Monte Carlo simulations (2000 repetitions)
	Methods for sensitivity	Broadened definition of SSBs to include fruit juice and milk products; adjust effect assuming retailers respond by reducing the non-discounted

		retail price of SSBs, threshold analysis to determine the decrease in average non-discounted price of relevant SSBs that would result in the intervention remaining cost effective, threshold analysis to determine duration of effect to remain cost-effective, threshold analysis exploring compensatory consumption.
	Brief summary of results (incl. sensitivity results)	Total Health Adjusted Life Years gained were estimated at 34,260 (95%UI: 24,922–45,504). Estimated costs were AUD17.0 million, with estimated healthcare cost savings of AUD376.0 million. The intervention was considered dominant (cost-saving and health promoting). The intervention remained cost-effective if retailers reduced average non discounted SSB prices in response to the intervention by less than 5.36%.
	Limitations	Effect may not be fully transferable from UK source. Use of dietary data from a self-report survey. Unknown response from retailers and manufacturers to the policy.
	Funding source	National Health and Medical Research Council, Australian Research Council
	Conflicts of interest	None declared
**Kenney et al.(33)**		
28	Study aim	To estimate the cost-effectiveness of installation of chilled water dispensers (“water jets”) on school lunch lines and to compare water jets’ cost, reach, and impact on water consumption with three additional strategies.
	Country	US
	Currency unit and year	USD 2015
	Study design	CEA
	Setting	Schools
	Intervention	1) Grab a Cup, Fill it Up, placement of promotional signage<br>2) Portable water dispensers<br>3) Bottle-less water coolers<br>4) Installation of water jet dispensers on school lunch lines

	Comparator	No intervention
	Target population	Students in kindergarten through eighth grade (K-8) attending schools that participate in the NSLP (about 64,956 schools) and thus would be subject to the drinking water requirements. 93% of NSLP participating schools (approximately 60,409 schools) would have viable plumbing allowing safe access to drinking water and would be eligible to implement the interventions.<br><br>1) Grab a Cup, Fill it Up: 45.7% (about 27,607 schools) would have existing tap water sources inside the school cafeteria making them eligible. 100% of eligible schools would implement. 13.5 million children in the first year.<br>2) Portable water dispensers - 100% of eligible schools would implement. 29.6 million children in the first year.<br>3) Bottle-less water coolers – 100% of eligible schools would implement. 29.6 million children in the first year.<br>4) Installation of water jets- 100% of eligible schools would implement. 29.6 million children in the first year.
	Perspective	Modified societal perspective
	Time horizon	10 years
	Model specification (if applicable)	CHOICES microsimulation model
	Measurement and valuation of outcomes	Evidence from the literature to estimate impact on water intake (natural experimental studies and randomized controlled trials)<br><br>One intervention used one relevant study from the literature<br>Change in water consumption-change in SSB intake-BMI<br>Healthcare cost-savings of obesity averted
	Measurement and valuation of resources and cost	Resource use estimated following a costing protocol following standard guidelines for resource identification, measurement, and valuation, and based on evaluations of similar interventions where possible.
	Discount rate	3%

	Methods for uncertainty	1,000 Monte Carlo iterations for a simulated population of 1 million individuals' representative of the national population
	Methods for sensitivity	Sensitivity for installation of water jets varied % of eligible schools who would implement (39.3%). Also varied estimate of effect to more conservative.
	Brief summary of results (incl. sensitivity results)	Installing water jets on school lunch lines was projected to reach 29.6 million children (95% uncertainty interval [UI]: 29.4 million-29.8 million), cost $4.25 (95% UI: $2.74-$5.69) per child, prevent 179,550 cases of childhood obesity in 2025 (95% UI: 101,970-257,870), and save $0.31 in health care costs per dollar invested (95% UI: $0.15-$0.55). In the secondary analysis, installing cup dispensers next to existing water fountains was the least costly but also had the lowest population reach.
	Limitations	True implementation costs, cost savings, and impact are unknown. Assumed that the average effects observed in the original study population were generalisable to the larger US population.
	Funding source	Not stated
	Conflicts of interest	Not stated
**Kenney et al.(34)**		
29	Study aim	To quantify the potential population-wide costs, number of individuals reached, and impact on obesity of five effective interventions to reduce children's television viewing if implemented nationally.
	Country	US
	Currency unit and year	USD 2019
	Study design	Modelled CEA
	Setting	Policy, ECEC, maternal child health
	Intervention	1) Eliminating the tax deductibility of food and beverage advertising, 2) Targeting TV reduction during home visiting programs, 3) Motivational interviewing to reduce home television time during Women, Infants, and Children (WIC) clinic visits, 4) Adoption of a television-reduction

		curriculum in child care; and 5) Limiting noneducational television in licensed child care settings.
	Comparator	No intervention
	Target population	Consulted advisory group for plausible reach and reviewed grey literature<br><br>1) Eliminating the tax deductibility of food and beverage advertising: children aged 2-19 years: 106 million children, 2) Targeting TV reduction during home visiting programs: 4-7 year olds with BMI >75th percentile who are eligible for a home visiting program: 0.380 million children, 3) Motivational interviewing to reduce home television time for WIC clinic visits: 2-4 year olds: 8.81 million children, 4) Adoption of a television-reduction curriculum in child care: 2-5 year olds: 5.65 million children; and 5) Limiting noneducational television in licensed child care settings: 2-5 year olds: 9.39 million children.
	Perspective	Societal
	Time horizon	Ten years (2020-2030)
	Model specification (if applicable)	CHOICES microsimulation model
	Measurement and valuation of outcomes	Published estimates from randomized controlled trials and quasi-experimental evaluations of each intervention's impact on hours of television viewed per day.<br><br>Change in television viewing – BMI using two studies from the literature
	Measurement and valuation of resources and cost	Standard guidelines for identifying resource use and cost.<br>Costs were identified using estimates of resource use from the original intervention studies or by consulting with individuals with direct experience implementing similar programs.<br>Healthcare cost-savings from obesity averted
	Discount rate	3%
	Methods for uncertainty	1000 Monte Carlo iterations for a simulated nationally representative population of 1 million individuals.

	Methods for sensitivity	Assumed that the impact of an hour of TV viewing on BMI would have declined proportionately to the amount that children's exposure to food and beverage advertising is estimated to have declined from 2007 until 2017.
	Brief summary of results (incl. sensitivity results)	Eliminating the tax deductibility of food advertising could reach the most children [106 million, 95% uncertainty interval (UI): 105–107 million], prevent the most cases of obesity (78,700, 95% UI: 30,200–130,000), and save more in health care costs than it costs to implement. Strategies targeting young children in child care and WIC also cost little to implement (between $0.19 and $32.73 per child reached), and, although reaching fewer children because of the restricted age range, were estimated to prevent between 25,500 (95% UI: 4600–59,300) and 35,400 (95% UI: 13,200–62,100) cases of obesity. Home visiting to reduce television viewing had high costs and a low reach
	Limitations	Could not estimate the impact of reducing excessive mobile device use. The modelled effect of the ECE television regulations strategy was based on hypothetical impacts on television viewing, not on effect measures obtained from intervention trials. Unable to explore heterogeneity between population sub-groups.
	Funding source	The JPB Foundation (Grant #1085).
	Conflicts of interest	None declared
**Killedar et al.(35)**		
30	Study aim	To conduct an economic evaluation of the CHAT trial to prevent childhood obesity.
	Country	Australia
	Currency unit and year	AUD 2018
	Study design	Within-trial CEA
	Setting	Telehealth
	Intervention	Nurse-led telephone advice, involving nine telephone support sessions of approximately 30 to 60 minutes each, information booklets.

		SMS advice, involving twice weekly text messages over four weeks at each of the nine developmental stages, information booklets, SMS support from research nurses
	Comparator	Usual care plus mailed resources on child safety, upscaling costs of telephone and SMS interventions were compared to the original Healthy Beginnings home-visiting intervention costs.
	Target population	1155 pregnant women in the third trimester
	Perspective	Health-payer perspective
	Time horizon	Within-trial (birth to age two years)
	Model specification (if applicable)	NA
	Measurement and valuation of outcomes	BMI and BMIz at 24 months. Both the telephone and SMS interventions resulted in non-significantly lower BMI and BMI-z than in the control group. The mean effect sizes for the telephone intervention were 0.048 kg/m^2 for BMI and 0.025 for BMIz. The mean effect sizes for the SMS intervention were 0.034 kg/m^2 for BMI and 0.018 for BMIz.
	Measurement and valuation of resources and cost	The costs counted included costs of all resources needed to reproduce the intervention but excluded any research and development costs. The costs of the telephone intervention included the costs of mobile phones, service provider costs of telephone calls, nurse time, training of nurses, payment of interpreters, administration time, and educational materials. The SMS intervention included the costs of training of nurses, time taken for sending and responding to texts, and service provider costs. Other health care resources used during the trial period included the number of GP and medical specialist visits for the child participant and this was determined through mother self-report at child aged 2 years.
	Analytics and assumptions	ICERs. The difference in overall costs (intervention and health care) among the intervention and control groups was divided by the difference in outcomes.
	Discount rate	5%
	Methods for uncertainty	Bootstrapping; cost-effectiveness acceptability curves

	Methods for sensitivity	Limited societal perspective – including the opportunity cost of missed days of work from mothers' self-reported days of work or usual activities missed.
	Brief summary of results (incl. sensitivity results)	At child age 2 years, the SMS delivery was more cost-effective ($5154 per unit BMI and $979 per 0.1 BMI z score units avoided) than the telephone delivery ($10,665 per unit BMI and $2017 per 0.1 BMI z score units avoided). The costs of upscaling the SMS ($7.64 million) and the telephone delivery modes ($37.65 million) were lower than the home-visiting intervention ($108.45 million). Including the productivity costs increased the ICER, but the ICER for SMS remained more favorable than for telephone.
	Limitations	Loss to follow up and missing data limited analyses. Healthcare costs and productivity losses were subject to potential recall bias.
	Funding source	New South Wales Health Translational Research Grant Scheme 2016 (ID number: TRGS 200), the Australian National Health and Medical Research Council (NHMRC) Partnership Projects (APP1169823), and the NHMRC Centre of Research Excellence in Early Prevention of Obesity in Childhood (EPOCH CRE) (APP1101675).
	Conflicts of interest	None declared
**Lal et al.(12)**		
31	Study aim	To assess the potential cost-effectiveness, health gains, and financial impacts by socioeconomic position (SEP) of a 20% SSB tax for Australia.
	Country	Australia
	Currency unit and year	AUD 2010
	Study design	Modelled CUA
	Setting	Policy
	Intervention	20% SSB tax
	Comparator	No intervention
	Target population	2010 Australian population aged 2-100 years

	Perspective	Societal perspective, sorted by health sector, government, industry, and private
	Time horizon	Lifetime
	Model specification (if applicable)	CRE Obesity model, Markov cohort model
	Measurement and valuation of outcomes	Effect estimated using intake data and price elasticities from the literature.<br>Change in intake-change in energy intake-BMI-HALYs
	Measurement and valuation of resources and cost	Costing methods from US study of excise tax converted to equivalent costs. Assumed cost to the beverage industry to be equal to cost to government, based on sales tax evidence. Cost of legislation estimated.<br>Healthcare cost-savings from cases of disease prevented.<br>OOP healthcare costs from government data.<br>Healthcare cost-savings from cases of disease averted.
	Discount rate	3%
	Methods for uncertainty	2,000 Monte Carlo simulations using Ersatz version 1.3 software
	Methods for sensitivity	One-way sensitivity analyses to explore the effect of including flavoured milk in the SSBs. SSB tax rate of 30% and a 50% pass-through of the 20% tax. Another mechanism for implementing a tax—a 50¢ per litre volumetric tax was also tested.
	Brief summary of results (incl. sensitivity results)	A 20% SSB tax would lead to HALY gains of 175,300 (95% CI: 68,700; 277,800) and healthcare cost savings of AU$1,733 million (m) (95% CI: $650m; $2,744m) over the lifetime of the population, with 49.5% of the total health gains accruing to the 2 lowest quintiles.
	Limitations	Best estimate of a potential effect in the absence of stronger direct evidence. Does not include other benefits (e.g. oral health). Some costing frameworks from international examples, in lieu of available Australian examples.
	Funding source	National Health and Medical Research Council, Centre for Research Excellence in Obesity Policy and Food Systems grant (APP1041020)
	Conflicts of interest	An author associated with supermarket chain collaborative project, provided sales data.

Long et al.(36)		
32	Study aim	To estimate the expected health and economic benefits of a national sugar-sweetened beverage excise tax of $0.01/ounce over 10 years.
	Country	US
	Currency unit and year	USD 2015
	Study design	Modelled CUA
	Setting	Policy
	Intervention	SSB tax of $0.01/ounce
	Comparator	No intervention
	Target population	US 2014 population
	Perspective	Societal
	Time horizon	10 years (2015-2025)
	Model specification (if applicable)	Markov cohort model (based on ACE-Obesity)
	Measurement and valuation of outcomes	Price elasticities of demand from the literature. Change in BMI from reduced consumption from studies from the literature.<br>BMI-DALYs
	Measurement and valuation of resources and cost	Implementation cost based on administrative data from two states operating soft drink excise taxes. Parallel evidence suggesting cost of compliance and cost of implementation equivalent. Intervention cost estimated based on personal communication with West Virginia State Department of Revenue.<br>Healthcare cost-savings from cases of disease averted
	Discount rate	3%
	Methods for uncertainty	Monte Carlo simulation (10,000 iterations), modifying primary scenario with alternative logic pathways
	Methods for sensitivity	Assumed tax "pass-through" rates varied from 50% to 150%, lowest own-price elasticity from review in the literature used, alternative program cost tested.
	Brief summary of results (incl. sensitivity results)	From 2015 to 2025, the policy would avert 101,000 disability-adjusted life-years (95% UI=34,800, 249,000); gain 871,000 quality-adjusted life-

		years (95% UI=342,000, 2,030,000); and result in $23.6 billion (95% UI=$9.33 billion, $54.9 billion) in healthcare cost savings. The tax would generate $12.5 billion in annual revenue (95% UI=$8.92, billion, $14.1 billion).
	Limitations	Potential for lack of generalisability from effect studies in the literature to implemented intervention at scale. Best estimate of a potential effect in the absence of stronger direct evidence.
	Funding source	Robert Wood Johnson Foundation (No. 66284), Donald and Sue Pritzker Nutrition and Fitness Initiative, JPB Foundation, and Prevention Research Center supported by Cooperative Agreement U48/DP001946 from CDC
	Conflicts of interest	Not stated
**Long et al.(37)**		
33	Study aim	To evaluate the potential cost-effectiveness of and stakeholder perspectives on a sugar-sweetened beverage (SSB) excise tax and a Supplemental Nutrition Assistance Program (SNAP) policy that would not allow SSB purchases.
	Country	US
	Currency unit and year	USD 2015
	Study design	Modelled CUA
	Setting	Policy
	Intervention	Sugar-sweetened beverage (SSB) excise tax of $0.01/oz and a Supplemental Nutrition Assistance Program (SNAP) policy that would not allow SSB purchases with SNAP funds.
	Comparator	No intervention
	Target population	Maine 2015 population<br>1.4 million people– SSB tax<br>311,000 people – SNAP SSB restriction
	Perspective	Not clearly stated
	Time horizon	10 years (2017-2027)
	Model specification (if applicable)	CHOICES microsimulation model

	Measurement and valuation of outcomes	Systematic review of SSB price elasticities used to model reduction in purchases.<br>Change in BMI from purchases from published studies.<br><br>SSB restriction model effects estimated using assumptions and intake and purchase data.<br><br>Purchase change – BMI – DALYs
	Measurement and valuation of resources and cost	SSB tax was costed based on local excise tax administration data from other Maine agencies. Assumption that the industry costs to implement the SSB tax would be equivalent to government effort to administer the tax.<br><br>SNAP intervention was costed based on data from the SNAP Healthy Incentives Pilot in Massachusetts, discussions with SNAP program officers and a former supermarket executive as part of the CHOICES project, and discussions with a supermarket executive and official in the Maine Department of Health and Human Services.<br><br>Healthcare cost-savings from cases of obesity averted
	Discount rate	3%
	Methods for uncertainty	Monte Carlo simulation (1,000 iterations)
	Methods for sensitivity	Not clearly stated
	Brief summary of results (incl. sensitivity results)	The SSB and SNAP policies were projected to save 3,560 QALYs (95% UI, 1,447−8,361) and 749 QALYs (95% UI, 415−1,168), respectively. The SSB and SNAP policies were estimated to reduce health care costs by $78.3 million (95% uncertainty interval [UI], $31.7 million−$185 million) and $15.3 million (95% UI, $8.32 million−$23.9 million), respectively
	Limitations	Estimates of effect in the absence of stronger direct evidence.
	Funding source	The JPB Foundation.

	Conflicts of interest	Not stated
**Magnus et al.(38)**		
34	Study aim	To estimate the cost-effectiveness of fiscal measures applied in remote community food stores for Aboriginal Australians.
	Country	Australia
	Currency unit and year	AUD 2011
	Study design	Modelled CUA
	Setting	Retail
	Intervention	Six fiscal strategies involving a 20% discount in the price of: 1) all fruit (fresh, dried, frozen, and tinned); 2) fresh vegetables only; 3) all vegetables (fresh, dried, frozen and tinned); 4) all F&V; 5) diet drinks and water; and 6) all F&V, plus diet drinks and water.
	Comparator	Current practice, no price discounts
	Target population	Remote population of Aboriginal and Torres Strait Islanders in 2011 (2.5% of entire Australian population)
	Perspective	Societal perspective
	Time horizon	Lifetime
	Model specification (if applicable)	Multistate lifetable model
	Measurement and valuation of outcomes	Empirical food purchase data – published price elasticity data – modelled improvement in diet-related outcomes – DALYs.
	Measurement and valuation of resources and cost	Modelled costs of the fiscal strategies included reimbursement of the price discount to store management, and time costs associated with advertising and ticketing the price changes<br>Nutrition education strategy costed based on unpublished case study in one remote community, and personal communication with Department of Health.<br>Healthcare cost-savings from cases of disease averted.
	Discount rate	3%
	Methods for uncertainty	Monte Carlo simulations (2,000 iterations)
	Methods for sensitivity	Addition of a nutrition education strategy in-store program

	Brief summary of results (incl. sensitivity results)	While dietary change was small, five of the six price discount strategies were estimated as cost-effective, below a $50,000/DALY threshold.
	Limitations	No published food price elasticity data for Australian Aboriginal and Torres Strait Islander populations. Gaps in national level Aboriginal and Torres Strait Islander health status data (BMI, blood pressure) and national dietary composition by age and gender. International price elasticity data.
	Funding source	The Cost of Dietary Improvement project was funded by the National Aboriginal and Torres Strait Islander Health Equality Council, and Menzies School of Health Research commissioned Deakin University to conduct economic analysis
	Conflicts of interest	None declared
**Magnus et al.(39)**		
35	Study aim	To model the health benefits and cost-effectiveness of banning television (TV) advertisements in Australia for EDNP food and beverages during children's peak viewing times.
	Country	Australia
	Currency unit and year	AUD 2001
	Study design	Modelled CEA and CUA
	Setting	Policy, media
	Intervention	Extension of existing regulations within the Children's Television Standards to preclude advertising for unhealthy foods, as well as for beverages and fast food outlets, during specified children's TV viewing hours and where a substantial proportion of children aged 5–14 years were in the viewing audience.
	Comparator	Current practice, specified according to the Children's Television Standards
	Target population	All children aged 5-14 years in Australia in 2001 (2.4M children)
	Perspective	Societal
	Time horizon	Lifetime

	Model specification (if applicable)	ACE model (ACE-Obesity project), a multi-state, multiple cohort life table model. The Australian children by sex and five-year age groups were modelled through a deterministic Markov model of one-year cycles, with relevant hazards and marginal costs until the whole cohort had died or reached 100 years of age. The main benefits from the intervention arose from differences in estimated mortality, morbidity and health costs of future disease, based on a changing distribution of BMI. Assumed maintenance of effect into adulthood.
	Measurement and valuation of outcomes	Logic modelled for effect estimate using the literature. Exposure – reduced consumption estimate from literature – BMI- DALYs.
	Measurement and valuation of resources and cost	Assumptions to inform resources used. The incremental costs of stricter monitoring and the enforcement of tightened regulations were estimated to be quite minimal with two extra staff at the Australian Communications and Media Authority (salary plus on-costs), as a regulatory framework already existed. Cost offsets were assessed as future health sector costs saved because of fewer occurrences of obesity-related conditions in the adult life of children exposed to the intervention.
	Discount rate	3%
	Methods for uncertainty	Uncertainty around distributions, modelled using Monte Carlo simulations (2000 iterations).
	Methods for sensitivity	The following scenarios were modelled as univariate sensitivity tests:<br>1) 30 extra staff to allow for the possibility that broadcasters may not fully comply with the regulations, 2) Comparative use of the Swinburn method to evaluate the impact of a less conservative reduction in grams/day of EDNP beverages on weight and BMI.<br><br>Threshold analysis was also undertaken to ascertain how high or low key parameters needed to be before the intervention became cost-ineffective, where the 'cost-effectiveness threshold' was specified as $AUD50,000 per DALY averted. Also tested elimination of the benefit maintenance assumption and the revenue impact on industry of the lost EDNP food sales assumption.

	Brief summary of results (incl. sensitivity results)	The intervention had a gross incremental cost-effectiveness ratio of AUD$ 3.70 (95% uncertainty interval (UI) $2.40, $7.70) per DALY. Total DALYs saved were 37 000 (95% UI 16 000, 59 000). The intervention was 'dominant', because it resulted in both a health gain and a cost offset compared with current practice. The intervention remained dominant in both sensitivity analyses.
	Limitations	Modelled effect estimates and costs. These results assumed full maintenance of BMI benefit through adulthood.
	Funding source	Victorian Government Department of Human Services, Australia
	Conflicts of interest	None declared
**Mantilla-Herrera et al.(13)**		
36	Study aim	To estimate the cost-effectiveness of the Health Star Rating (HSR) system, a voluntary front-of-pack labelling (FoPL) initiative.
	Country	Australia
	Currency unit and year	AUD 2010
	Study design	Modelled CUA
	Setting	Policy
	Intervention	HSR system, a voluntary front-of-pack labelling (FoPL) initiative under two scenarios: (1) the current voluntary scheme; and (2) implementation on a mandatory basis.
	Comparator	No intervention
	Target population	Australian 2010 population
	Perspective	Limited societal
	Time horizon	Lifetime
	Model specification (if applicable)	Multi-state life table Markov model (CRE Obesity model)
	Measurement and valuation of outcomes	Pre- and post-implementation data on energy density of food products – consumption – BMI – HALYs<br>Baseline analysis assumed that 100% of observed changes in energy density before and after the HSR implementation were attributable to the intervention.

	Measurement and valuation of resources and cost	Intervention costs were obtained from a recent Australian government-commissioned report.<br>Healthcare cost-savings from cases of disease averted.
	Discount rate	3%
	Methods for uncertainty	Monte Carlo simulation based on 2000 uncertainty iterations
	Methods for sensitivity	Tested the impact of lower proportions of HSR-attributable energy intake (i.e., 50%, 30% and 10%) in univariate sensitivity analyses under the voluntary scenario.
	Brief summary of results (incl. sensitivity results)	The HSR system evaluated via changes in reformulation could be considered cost-effective relative to a willingness-to-pay threshold of A$50,000 per HALY (voluntary: A$1728 per HALY [95% UI: dominant to 10,445] and mandatory: A$4752 per HALY [95% UI: dominant to 16,236]).
	Limitations	Homogeneity assumptions within age-sex group cohorts, the intervention was evaluated as if implemented over the lifetime, no time lag incorporated. Additional impacts of the policy also not incorporated.
	Funding source	National Health and Medical Research Council, Australian Research Council.
	Conflicts of interest	An author associated with the New Zealand HSR Advisory Group, no influence on publication, and also interacts with large corporations in food processing industry.
**Mizdrak et al.(40)**		
37	Study aim	To estimate the health impacts, costs, and cost-effectiveness of a one-off national mass media campaign to promote the use of physical activity apps.
	Country	New Zealand
	Currency unit and year	NZD 2011
	Study design	Modelled CUA
	Setting	Media
	Intervention	One-off mass media campaign to promote high quality smartphone apps for physical activity.

	Comparator	No intervention. Assuming a low or no PA app promotion environment.
	Target population	New Zealand adults 15-79 years of age eligible. Of the population eligible, the proportion of the population that would experience increased physical activity based on likely awareness of the mass media campaign, app download rates, and app use was estimated.
	Perspective	Health system
	Time horizon	Lifetime
	Model specification (if applicable)	Multistate life table model to estimate the lifetime health gains (in QALYs) that would accrue if New Zealand adults were exposed to a one-off national mass media campaign to promote physical activity app use, with a 1-year impact on physical activity, compared to business-as-usual.
	Measurement and valuation of outcomes	Increase in PA from systematic review and meta-analysis, adjusted for estimate of adherence to app use (1 year effect). Increase in MET mins/week – QALYs.
	Measurement and valuation of resources and cost	Based on costs from a previous NZ study of the costs associated with a modeled mass media campaign to promote a weight loss app.
	Discount rate	3%
	Methods for uncertainty	Monte Carlo simulation (2000 runs)
	Methods for sensitivity	0% and 6% discount rates, "equity adjustment" that set background mortality and morbidity rates for Māori to non-Māori values; narrowing the population targeted by the intervention to those 40-80 years of age; 5-year maintenance of additional physical activity levels followed by a return to preintervention levels
	Brief summary of results (incl. sensitivity results)	The modeled intervention resulted in 28 QALYs (95% uncertainty interval [UI] 8-72) gained at a cost of NZ $81,000/QALY (2018 US $59,500; 95% UI 17,000-345,000), over the remaining life course of the 2011 New Zealand population. The intervention had a low probability (20%) of being cost-effective at a cost-effectiveness threshold of NZ $45,000 (US $32,900) per QALY. The health impact and cost-effectiveness of the intervention were highly sensitive to assumptions

		around the maintenance of physical activity behaviors beyond the duration of the intervention.
	Limitations	The assumption of disease independence; the use of a health-system perspective for costs and benefits; the assumption of no heterogeneity in intervention impact.
	Funding source	Health Research Council of New Zealand (HRC16/443)
	Conflicts of interest	None declared
**Moodie et al.(41)**		
38	Study aim	To assess from a societal perspective the incremental cost-effectiveness of the WSB program for Australian primary school children as an obesity prevention measure.
	Country	Australia
	Currency unit and year	AUD 2001
	Study design	Modelled CUA
	Setting	Schools, community
	Intervention	Walking School Bus program, involving two volunteer adult "conductors" (at a ratio of 1 adult to 8 children) and travel along a set route through a neighbourhood picking up children along the way and delivering them to school.
	Comparator	Current practice, defined as 'do nothing'
	Target population	Children new to active transport (50% × 15,680) from the Australian childhood population (age 5-7 years); 7,840 children
	Perspective	Societal
	Time horizon	Lifetime
	Model specification (if applicable)	ACE model (ACE-Obesity project), a multi-state, multiple cohort life table model. Detailed in separate paper.
	Measurement and valuation of outcomes	Modelled using a range of data, including evidence from the literature and assumptions. Increase in walking for previously inactive children – change in metabolic equivalent task – change in energy expenditure – change in BMI. Mean time spent walking taken from one study informed increased time spent walking.

	Measurement and valuation of resources and cost	Likely costs identified using pathway analysis. Costs included time cost of central coordination and recruitment of local governments and schools; recruitment, registration and training of volunteers; time and equipment costs of local planning phase; time and equipment cost of routine operation of WSB.
	Discount rate	3%
	Methods for uncertainty	Simulation modelling to present an uncertainty interval around the health benefits, costs and ICER (Monte Carlo simulation, 4000 iterations).
	Methods for sensitivity	The following scenarios were modelled as univariate sensitivity tests: attribution of 50% of costs to non-obesity related objectives (such as reducing traffic congestion), annuitisation of fixed costs, improvements in capacity utilisation and recruitment (by increasing the number of children per WSB, WSBs per school and schools per local government, and by increasing the pro- portions of local governments involved and of participants new to active transport). The impact of combining these measures was tested under both an "optimistic" and a "very optimistic" scenario.<br><br>Cost-effectiveness plane presented, alongside other factors likely considered in decision-making (second stage filter analysis).
	Brief summary of results (incl. sensitivity results)	The modelled intervention reached 7,840 children aged 5 to 7 years and cost $AUD22.8M ($16.6M; $30.9M). This resulted in an incremental saving of 30 DALYs (7:104) and a net cost per DALY saved of $AUD0.76M ($0.23M; $3.32M).<br><br>Cost-cutting measures including attribution of a portion of the total intervention costs to non-obesity related objectives and the annuitisation of fixed costs alone were insufficient to make the intervention cost-effective. When the improved capacity utilisation measures were added to the cost cutting measures, the intervention was cost- effective, but only under the 'very optimistic" scenario. The inclusion of an increase in

		the proportion of children receiving the benefit meant the intervention approached cost-effectiveness under the "optimistic" scenario.
	Limitations	Limited evidence of effectiveness. Data limitations on the population exposed. Information gaps to inform modelling.
	Funding source	Victorian Government Department of Human Services, Australia.
	Conflicts of interest	None declared
**Moodie et al.(42)**		
39	Study aim	To assess from a societal perspective the cost-effectiveness of the AASC program, a key plank of the former Australian Government's obesity prevention program.
	Country	Australia
	Currency unit and year	AUD 2001
	Study design	Modelled CUA
	Setting	Schools and after school settings
	Intervention	AASC, involving physical activity coordinators appointed to work with national, state, and regional sporting organisations to develop and deliver a physical activity program specific to the needs of each school/service. Selected sites were required to offer 2–3 sessions per week, depending on student numbers, for 8 weeks for each of four school terms per year.
	Comparator	No intervention
	Target population	The Victorian experience of recruiting schools to the AASC program was extrapolated to the Australian situation. The number of potential sites at a national level (3,300) was based on the AASC's appointment of 165 regional coordinators with an accepted average ratio of one coordinator per 20 sites. An average of 25 children per site was assumed. This translated to a total of 82,500 children involved in the program. Assumed 50% not active after school, 20% received more activity. The 70% who gained the program benefit were assumed to be equally spread across all grades from Prep to Grade 6. The estimated number of children receiving the intervention benefit was 69,300.
	Perspective	Societal

	Time horizon	Rest of life or 100 years (lifetime)
	Model specification (if applicable)	ACE model, multi-state, multiple cohort life table model (ACE-Obesity project)
	Measurement and valuation of outcomes	Logic pathway modelling. Change in physical activity (inactive to active, assuming change in METs from sitting quietly to playing sport) – change in BMI – change in DALYs. Assumed no substitution effects of energy intake or expenditure.<br><br>Modeled as if applied to the Australian population for 1 year in steady-state operation.
	Measurement and valuation of resources and cost	Pathway analysis was used to identify the component activities of the intervention in order to ascertain the associated resource utilization. The intervention was simulated on the basis of the service delivery model operated in Victoria by the Australian Sports Commission, although where information was available on differences between states, this was taken into account in the modelled cost-effectiveness analysis. Time, equipment for central co-ordination and recruitment of schools/OSHCs to the program, program delivery planning, routine operation of the program, routine support, monitoring and evaluation. Healthcare cost-savings from diseases averted.
	Discount rate	3%
	Methods for uncertainty	Simulation-modeling techniques (using the @RISK software and Monte Carlo simulations, 3000 iterations) were used to facilitate the presentation of a 95% uncertainty range around the health benefits, costs, and ICERs.
	Methods for sensitivity	Sensitivity analysis was undertaken to test the impact of changing key design features of the intervention. Univariate sensitivity tests: 1) reduction in the ratio of sites per regional coordinator from 1:20 to 1:30, 2) reduction in the number of state level coordinators from 18 to 11, 3) application of the same wage rate to all site coordinators irrespective of the site being a school or OSHC, 4) a combination of scenarios 1), 2), and 3), 5) all participants receive full intervention benefit

	Brief summary of results (incl. sensitivity results)	For 1 year, the intervention cost is Australian dollars (AUD) 40.3 million (95% uncertainty interval AUD 28.6 million; AUD 56.2 million), and resulted in an incremental saving of 450 (250; 770) DALYs. The resultant cost-offsets were AUD 3.7 million, producing a net cost per DALY saved of AUD 82,000 (95% uncertainty interval AUD 40,000; AUD 165,000).<br>None of the sensitivity analyses were under the cost-effectiveness threshold. If 100% of all participants received the full intervention, it would approach cost-effectiveness (unlikely).
	Limitations	Absence of some data; assumptions regarding modeling and participation needed.
	Funding source	Victorian Government Department of Human Services, Australia
	Conflicts of interest	None declared
**Moodie et al.(43)**		
40	Study aim	To assess the cost-effectiveness of a school program to increase active transport in 10- to 11-year-old Australian children as an obesity prevention measure.
	Country	Australia
	Currency unit and year	AUD 2001
	Study design	Modelled CUA
	Setting	Primary schools
	Intervention	A curriculum-based program specifically targeted at children in years 5 and 6 (age 10 and 11 years), and piloted in six Victorian schools (the TravelSmart Schools program). Program aimed to increase awareness about physical environments impacting active transport, decrease traffic around schools, increase physical activity levels and raise capacity. The program included meetings, professional development for teachers, classroom activities for students, whole school activities and events and promotion of the program in the community.
	Comparator	Current practice, no intervention

	Target population	Australian primary school children in years 5 and 6. The modeled intervention reached 267,700 children.
	Perspective	Societal
	Time horizon	Lifetime (rest of life or 100 years)
	Model specification (if applicable)	ACE-Obesity (ACE-Obesity project), a multi-state, multiple cohort life table model. The intervention was modeled for 1 year in steady-state operation.<br><br>ICERs calculated as the cost ($AUD) per Disability- Adjusted Life Year (DALY) saved, as well as the cost per Body Mass Index (BMI) unit saved.<br>Second stage filter analysis captured less quantifiable aspects important to decision-makers.
	Measurement and valuation of outcomes	Modelled estimates of impact on child BMI. Pathway analysis from change in physical activity – change in BMI – DALYs. Estimates of changes in active transport taken from the literature (one pilot study), and other available data used to model likely change in BMI by mode.<br><br>Estimates of change in physical activity from a pre-post survey of parents, with low response rates, were used to model effectiveness in a sensitivity analysis.
	Measurement and valuation of resources and cost	Pathway analysis was used to identify the component activities of the intervention. Costs included program co-ordination, backfill teachers for training, training costs, travel time and costs to training, venue hire, catering, teacher time delivering lessons, manuals, whole of school events. Some assumptions required to cost intervention.
	Discount rate	3%
	Methods for uncertainty	Simulation-modeling techniques (using the @RISK software and Monte Carlo simulations based on 3000 iterations) allowed for the calculation of a 95% uncertainty range around the median health benefits, costs, and ICERs.

	Methods for sensitivity	Univariate sensitivity tests: 1) joint cost attribution across multiple objectives, whereby 30%, 50%, 70%, and 80% of total costs were apportioned to transport (non obesity-related) objectives, 2) broadening of the benefit to include other children in the school who were assumed to take up active transport at half the rate of children in grades 5 and 6, and conservatively receive only half of the benefit, 3) exclusion of selected cost items (training venue hire, teacher travel time, and vehicle operating costs).<br><br>Estimates of change in physical activity were from a pre-post survey of parents, with low response rates, were used to model effectiveness in a sensitivity analysis.
	Brief summary of results (incl. sensitivity results)	Intervention cost $AUD13.3M (95% uncertainty interval [UI] $6.9M; $22.8M) per year. Incremental saving of 890 (95%UI –540; 2,900) BMI units, which translated to 95 (95% UI –40; 230) DALYs and a net cost per DALY saved of $AUD117,000 (95% UI dominated; $1.06M).<br><br>The intervention became cost-effective (against the $AUD50,000 per DALY saved benchmark) when 55% or more of the costs were apportioned to non-obesity-related objectives. If the benefit was broadened, the ICER was more than halved, and approached cost-effectiveness. The exclusion of some costs such as teacher travel time to training, teacher vehicle operating costs, and the hire of a training venue had a negligible impact on the ICERs.
	Limitations	Assumption of 100% maintenance of benefit over time. Limited evidence of effectiveness. No data on changes in active transport participation in other students or parents/family members.
	Funding source	Victorian Government Department of Human Services, Australia.
	Conflicts of interest	Not stated

Moodie et al.(44)		
41	Study aim	To examine the cost-effectiveness of BAEW, a community-based capacity-building program that promoted healthy eating and physical activity for Australian children aged 4-12 years between 2003 and 2006
	Country	Australia
	Currency unit and year	AUD 2006
	Study design	Modelled CUA
	Setting	Whole of community and primary school setting
	Intervention	The BAEW program was conducted in the rural town of Colac (population 11,000) in the state of Victoria, Australia (27) and evaluated by Deakin University.
	Comparator	Current practice
	Target population	Australian children aged 4-12 years. Modelled for both the Colac population of eligible children, and if the program was delivered across Australia. Given the number of programs competing for time in the school setting, it was conservatively assumed that the intervention would be taken up by 10% of Australian primary schools. In the primary scenario, it was assumed that the benefit would be received by 100% of children in those schools, but the impact if a lower proportion received the benefit was tested under sensitivity analysis. When modeled for 10% of Australian primary school children, the intervention would reach 181,212 children across 656 schools.
	Perspective	Societal perspective
	Time horizon	Lifetime (rest –of life or 100 years)
	Model specification (if applicable)	ACE model, multistate lifetable model
	Measurement and valuation of outcomes	Outcomes measured as BMI and DALYs.
	Measurement and valuation of resources and cost	Intervention resource use was measured retrospectively from process evaluation reports, school newsletters, reports, and stakeholder interviews. Pathway analysis was used to identify the component activities of the intervention to ascertain the associated resource utilisation.

	Discount rate	3%
	Methods for uncertainty	Monte Carlo simulations based on 6000 iterations
	Methods for sensitivity	Various scenarios were tested to determine which components of the effect would need to be lost before intervention was cost-ineffective, including testing the impact of only 50% of children receiving the benefit.
	Brief summary of results (incl. sensitivity results)	Intervention cost was AUD0.34M ($0.31M; $0.38M) per year, and resulted in savings of 547 (-104; 1209) BMI units and 10.2 (-0.19; 21.6) DALYs. Results show modest cost offsets of AUD27 311 (-$1803; $58 242) and a net cost per DALY saved of AUD29 798 (dominated; $0.26M).
	Limitations	Assumption of 100% maintenance of effect likely to overstate the cost-effectiveness results. Mismatch between population and disease data from 2001, costed in 2006 means possible conservative gain in averted DALYs.
	Funding source	Victorian Government Department of Human Services, Australia
	Conflicts of interest	None declared
**Over et al.(45)**		
42	Study aim	To estimate the cost-effectiveness of counselling and pedometer use to increase physical activity.
	Country	Netherlands
	Currency unit and year	EUR 2009
	Study design	Modelled CUA
	Setting	General practice
	Intervention	GP counselling and pedometer use
	Comparator	Current practice
	Target population	Assumed that the percentage of GPs participating in the pedometer intervention would be equal to the percentage that offer a smoking cessation intervention currently in place (i.e. 35%-40%). Patients visiting these GPs would have access to the intervention. Uptake based on proportion of patients who are not sufficiently active from published

		study, and assumptions that 50% of these would accept a pedometer and attend follow up sessions.
	Perspective	Health care perspective
	Time horizon	Lifetime (or 100 years)
	Model specification (if applicable)	RIVM CDM), a Markov-type state-transition model
	Measurement and valuation of outcomes	Assumed to increase the physical-activity levels of those patients completing the pedometer intervention by 2491 steps per day after one year, which is the average increase in steps from a published meta-analysis.<br><br>Estimated a long-term pedometer effect of approximately 25% of the additional 2491 steps, namely 623 steps, which corresponds to 6 minutes of walking.<br><br>Health care cost-savings from disease averted
	Measurement and valuation of resources and cost	Bottom-up method to cost. Cost of equipment and GP time.
	Discount rate	Benefits 1.5%; costs 4%
	Methods for uncertainty	Probabilistic sensitivity analysis (100,000 iterations)
	Methods for sensitivity	Varied long term effects – 12.5%, 50% of 2491 steps
	Brief summary of results (incl. sensitivity results)	The intervention resulted in almost 6000 people shifting to more favourable physical-activity levels, and in 5100 life years and 6100 QALYs gained, at an additional total cost of EUR 67.6 million. The incremental cost-effectiveness ratio (ICER) was EUR 13,200 per life year gained and EUR 11,100 per QALY gained. Intervention probability of being cost-effective was 0.66 per QALY gained if valued at the Dutch informal threshold for cost-effectiveness of EUR 20,000.
	Limitations	Physical activity in the RIVM CDM was modeled via discrete classes: inactive, insufficiently active, sufficiently active rather than continuous, implied a somewhat crude calculation of health outcomes. Did not include broader societal costs which may have led to overestimation of ICER.

	Funding source	Not stated
	Conflicts of interest	None declared
**Robinson et al.(6)**		
43	Study aim	To estimate the cost-effectiveness of two potential policies that increase the price of alcohol in Australia: a volumetric tax applied to all alcohol (Intervention 1) and a minimum unit floor price (Intervention 2).
	Country	Australia
	Currency unit and year	AUD 2010
	Study design	Modelled CUA
	Setting	Policy
	Intervention	A volumetric tax applied to all alcohol of $1.07 per standard drink (Intervention 1) and a minimum unit floor price of $1.30 per standard drink (Intervention 2).
	Comparator	No intervention
	Target population	2010 Australian population aged 15 and older
	Perspective	Limited societal
	Time horizon	Lifetime or 100 years
	Model specification (if applicable)	ACE model (ACE-Obesity project), multi-state, multiple cohort life table model
	Measurement and valuation of outcomes	Estimated changes in alcoholic drink consumption and corresponding changes in energy intake were calculated using the 2011–12 Australian Health Survey data, published price elasticities, and nutrition information.<br>Energy intake – body weight – BMI - HALYs
	Measurement and valuation of resources and cost	Australian estimates of parliamentary legislation costs were based on the literature. Implementation, compliance, and monitoring costs were based on costings from the 2011–12 New South Wales (NSW) Fast Food Labeling review report. The cost of running a nation-wide education campaign to provide information and resources to consumers and industry regarding the intervention was based on the estimated costing for the consumer fast food labeling campaign run by the NSW

		Government. Due to the limited data around the real-world costs of either intervention, compliance costs for industry were based on estimates from the Scottish Government, following the recent introduction of a minimum floor price on alcohol in Scotland. Due to a lack of data availability, some downstream impacts and indirect costs such as reductions in industry revenue for the volumetric tax intervention and increases in industry revenue for the minimum floor price were not captured.
	Discount rate	3%
	Methods for uncertainty	Monte Carlo simulation using Ersatz software
	Methods for sensitivity	Assuming alcoholic beverages were substituted with SSBs
	Brief summary of results (incl. sensitivity results)	Both interventions were estimated to lead to reductions in mean alcohol consumption (Intervention 1: 20.7% (95% Uncertainty Interval (UI): 20.2% to 21.1%); Intervention 2: 9.2% (95% UI: 8.9% to 9.6%)); reductions in mean population body weight (Intervention 1: 0.9 kg (95% UI: 0.84 to 0.96); Intervention 2: 0.45 kg (95% UI: 0.42 to 0.48)); HALYs gained (Intervention 1: 566,648 (95% UI: 497,431 to 647,262); Intervention 2: 317,653 (95% UI: 276,334 to 361,573)); and healthcare cost savings (Intervention 1: \$5.8 billion (B) (95% UI: \$5.1B to \$6.6B); Intervention 2: \$3.3B (95% UI: \$2.9B to \$3.7B)). Intervention costs were estimated as \$24M for Intervention 1 and \$30M for Intervention 2. Both interventions were dominant, resulting in health gains and cost savings.<br><br>After accounting for substitution of alcoholic beverages with full sugar SSBs both interventions were found to result in net health losses. In this scenario, the Australian population was expected to lose 512,422 HALYs (95% UI: 448,487 to 584,831) from the introduction of a uniform volumetric tax on alcohol, and lose 275,526 HALYs (95% UI: 238,941 to 314,562) from a minimum floor price on alcohol.
	Limitations	Dated data on alcohol price, proportion of alcohol sold under \$1.30, and tax rates to calculate the change in price post-intervention. Scottish data to estimate resource use for industry costs in response to the two

		interventions, did not include industry profits (floor price). Self-reported alcohol consumption data. Price elasticity assumptions for different alcoholic beverage due to limited evidence.
	Funding source	National Health and Medical Research Council, Heart Foundation, Australian Research Council
	Conflicts of interest	None declared
**Roux et al.(46)**		
44	Study aim	To assess the cost-effectiveness of population-wide strategies to promote physical activity in adults.
	Country	United States
	Currency unit and year	USD 2003
	Study design	Modelled CUA
	Setting	Community
	Intervention	Community-wide campaigns, individually adapted health behavior change, social-support interventions in community settings, and the creation of or enhanced access to places for physical activity combined with informational outreach activities. A seventh, newer intervention study was added to better represent community-wide campaigns.
	Comparator	No intervention
	Target population	Simulated cohort of healthy US adults stratified by age, gender, and physical activity level. US adult population aged 25–64 years in 2004.
	Perspective	Societal perspective
	Time horizon	Lifetime
	Model specification (if applicable)	The CDC MOVE Model, a comprehensive flexible, state-transition Markov model
	Measurement and valuation of outcomes	Effects from controlled studies of each intervention.<br>The impact of an intervention was assumed to decline after the intervention had ended, and the decline in maintenance of physical activity levels over time was modeled based on the limited data from the research literature on the long-term maintenance of increased physical activity resulting from interventions

		METS-QALYS
	Measurement and valuation of resources and cost	Direct communication with the authors of original investigations in combination with a review of manuscript protocols, each original intervention was itemised to determine all associated costs.<br>Healthcare cost-savings from diseases averted.
	Discount rate	3%
	Methods for uncertainty	Probabilistic sensitivity analysis (Monte Carlo analysis)
	Methods for sensitivity	One-way, two-way, and probabilistic sensitivity analyses, with particular emphasis on intervention effect size and cost estimates. Shortening the analytic time-horizon from 40 to 30, 20, or 10 years. Repeating the intervention once after 20 years.
	Brief summary of results (incl. sensitivity results)	Cost-effectiveness ratios ranged between $14,000 and $69,000 per QALY gained, relative to no intervention. Results were sensitive to intervention-related costs and effect size.
	Limitations	Limited data on race/ethnicity-specific disease outcomes and physical activity and intervention effects, it was not possible to extend the model to assess the cost-effectiveness of interventions in subpopulations by race or ethnicity.<br>People not falling into one of the five disease categories of interest were considered to be members of the well population, which underestimates the potential impact of physical inactivity on other important diseases.
	Funding source	Robert Wood Johnson Foundation and the CDC Foundation.
	Conflicts of interest	None declared
**Rush et al.(47)**		
45	Study aim	To extrapolate the Project Energize program effects, initial costs, lifetime health treatment cost structures, quality-adjusted-life-years gained and increased life expectancy to the general and Maori child population of New Zealand.
	Country	New Zealand
	Currency unit and year	NZD 2011
	Study design	Modelled CUA

	Setting	Primary schools
	Intervention	Project Energize, a multicomponent program delivered in schools
	Comparator	No intervention
	Target population	Primary school children New Zealand, and Maori population, aged 6-8 and 9-11 years old, across 192 schools.
	Perspective	A total health care budget perspective (government-incurred costs and direct cost of intervention implementation)
	Time horizon	Lifetime
	Model specification (if applicable)	Previously used model, adapted to population and modelling risk of 14 obesity-related conditions
	Measurement and valuation of outcomes	Data from RCT on BMI - QALYs
	Measurement and valuation of resources and cost	The ongoing cost of the intervention per participant was estimated from Project Energize's 2010 budget. Costs were allocated evenly to all participants. No additional costs were associated with delivery of Project Energize.<br>Healthcare costs attributable to obesity
	Discount rate	3.5%
	Methods for uncertainty	Not clearly stated
	Methods for sensitivity	Univariate analyses that varied discount rate from 0% to 5%, increased cost of intervention, estimated using effect 95% UIs, varied decay and shortened model time horizon (60 years)
	Brief summary of results (incl. sensitivity results)	Incremental cost/ per QALY gained was $30,438 for the younger and $24,690 for the older children: the cost/QALY was lower for Maori ¯ ($28,241, $22,151). Project Energize, applied to the whole population, may improve quality and length of life, and when compared with other obesity prevention programs previously assessed with this model it would be relatively cost-effective from the health treatment payer's perspective.
	Limitations	Assumption that there were no changes independent of the program between the time periods. Only looks at change in relative weight for height for age

	Funding source	The Ministry of Health HEHA programme
	Conflicts of interest	Not stated
**Sonneville et al.(48)**		
46	Study aim	To model the effect of a national intervention that eliminates the tax subsidy of advertising nutritionally poor foods and beverages on TV to children aged 2–19 years.
	Country	United States
	Currency unit and year	USD 2014
	Study design	Modelled CEA and CUA
	Setting	Policy
	Intervention	The elimination of the tax subsidy of TV advertising costs for nutritionally poor foods and beverages advertised to children and adolescents that applies to TV programming watched on traditional TV and to TV advertising aired during children's programming defined as >35% child-audience share.
	Comparator	Current practice
	Target population	Children aged 2–19 years
	Perspective	Modified societal perspective.
	Time horizon	10 years (2015-2025)
	Model specification (if applicable)	Adapted and modified the ACE model (ACE-Obesity project), a multi-state, multiple cohort life table model, to develop a model for the Childhood Obesity Intervention Cost-Effectiveness Study (CHOICES)
	Measurement and valuation of outcomes	Change in daily TV hours to change in BMI using evidence from one RCT, in order to estimate the impact of change in advertising on change in BMI. Conservatively reduced estimates of reductions in BMI due to reductions in TV time by 25% to account for any potential effects of increased physical activity.<br>BMI-QALYs
	Measurement and valuation of resources and cost	Assumed the implementation of the intervention entailed minimal costs and included those related to processing and auditing, but not enacting, the new tax. Overhead costs of the tax system included administrative

		costs and expenses related to tax audits and litigation and were estimated at the cost of five full-time federal employees (based on assumption). The costs and labor associated with tax compliance by the food and beverage industry were assumed to be equal to the cost of administration reported by the government. A loss in revenue by companies that sell predominantly nutritionally poor foods would be expected, although purchasing of other healthier foods that are not covered by the proposed intervention would likely increase. Assumed that, industry wide, the reduction in sales of nutritionally poor foods would be offset by the increase in sales of other foods and that a loss in revenue by commercial broadcasters would likely be offset by new advertising contracts for other products.<br>Healthcare cost-savings from cases of disease averted
	Discount rate	3%
	Methods for uncertainty	Monte Carlo simulations (10,000-1M iterations) using @RISK software
	Methods for sensitivity	Secondary analysis used estimate of effect from a study of naturally occurring variations in fast food advertising in the US to levels of BMI and obesity,
	Brief summary of results (incl. sensitivity results)	The intervention would reduce an aggregate 2.13 million (95% UI=0.83 million, 3.52 million) BMI units in the population and would cost $1.16 per BMI unit reduced (95% UI=$0.51, $2.63). From 2015 to 2025, the intervention would result in $352 million (95% UI=$138 million, $581 million) in healthcare cost savings and gain 4,538 (95% UI=1,752, 7,489) quality-adjusted life-years.<br><br>The secondary scenario indicated similar but lower levels of cost effectiveness. Eliminating the tax subsidy of TV advertising of nutritionally poor foods and beverages to children and adolescents was estimated to reduce mean BMI by 0.013 (95% UI=0.008, 0.017) units among children aged 2–19 years, and the intervention would cost $2.24 per BMI unit reduced (95% UI=$1.28, $3.65).

	Limitations	Assumption that the intervention effect is maintained. The model relies on BMI-mediated health effects and does not incorporate additional expected reductions in metabolic and other diseases due to a reduction in intake of heavily advertised products high in sodium, saturated fat, and added sugar.
	Funding source	Robert Wood Johnson Foundation (66284), Donald and Sue Pritzker Nutrition and Fitness Initiative, JPB Foundation, National Collaborative on Childhood Obesity Research, CDC, including the Nutrition and Obesity Policy Research and Evaluation Network.
	Conflicts of interest	None declared
**Sutherland et al.(49)**		
47	Study aim	To estimate the cost and cost effectiveness of the Physical Activity 4 Everyone (PA4E1) intervention implemented in secondary schools located in low-income communities.
	Country	Australia
	Currency unit and year	AUD 2014
	Study design	Within-trial CEA
	Setting	Secondary schools
	Intervention	The intervention consisted of embedding seven physical activity strategies into the school community (more active physical education (PE) lessons; development of personal physical activity plans; delivery of a 10 week enhanced school sport program; conducting supervised recess and/or lunch physical activity opportunities; supportive school physical activity policy; and linking with the community and linking with parents). Six implementation strategies were delivered (an in-school physical activity consultant, executive support, teacher training, resources, prompts and monitoring reports).
	Comparator	Usual practice, physical activity curriculum
	Target population	All enrolled students in the target Grade (Grade 7) across the five intervention schools at baseline (n = 837). Scenarios regarding rollout: 254,923 students from 487 NSW schools.

	Perspective	Societal
	Time horizon	Within-trial, two years
	Model specification (if applicable)	NA
	Measurement and valuation of outcomes	Minutes of MVPA per day gained, measured using accelerometry; MET hour gained per person/ day; BMI (measured); BMIz
	Measurement and valuation of resources and cost	Retrospective using trial records. Resource use categories included personnel costs, materials and printing. Personnel costs included opportunity costs for the delivery of strategies by school staff and community sport and fitness providers. Personnel costs for the implementation of strategies that occurred outside of PE and sport time were valued using the opportunity cost of forgone time. Costs incurred for the intervention implementation strategies included personnel costs, equipment and travel/venue/meal expenses. Personnel costs included in-school consultant salary, payment of consultants to deliver PE teacher training, teacher relief to allow PE teachers to attend training, and opportunity costs (forgone time) associated with additional committee meetings about intervention implementation.
	Analytics and assumptions	Intention to treat; ICERs (cost per student per mean minute of MVPA gained and cost per student per MET minute gained, expenditure per student per BMI unit avoided, cost per student per 0.1 unit (10 %) BMI z-score reduction.
	Discount rate	Not specified
	Methods for uncertainty	Not undertaken as ICERs estimated using aggregated costs across schools and so not undertaken.
	Methods for sensitivity	Univariate sensitivity analyses included: variation in the costs of specific intervention components, variation in the magnitude of effect size using the upper and lower confidence interval limits, test assuming physical activity strategy 4 (recess and lunchtime activities) is extended to 10 % of students beyond the target grade, with a reduced effect on daily minutes of MVPA compared to students in the target grade, and assuming the benefits of physical activity strategy 1 (active PE), strategy

		5 (physical activity policy) and implementation strategy 1 (change agent), 2 (executive support) and 3 (resources) are extended to all students (100 %) outside the target year (in Grades 7–10), with a reduced effect on daily minutes of MVPA compared to students in the target grade.<br><br>Two additional scenario analyses explored the potential cost effectiveness of state-wide implementation of the intervention across NSW.<br><br>Statewide rollout (current model): total cost of intervention based on trial cost, extrapolated to population (current implementation support model), assumed benefit to 100% of students, effect size based on the results of the sensitivity analysis conducted within-trial (imputation of missing data), 254,923 students from 487 NSW schools.<br><br>Statewide rollout (alternate model) - addressed potential logistical challenges of scale and based on questions posed to principals of participating schools, adopted a real-world model where PA practice is support by an existing in-school teacher (rather than employed PA consultant in the trial). Cost of intervention modified to reflect alternate model of school support and a reduction in equipment cost per school. Reduction of equipment cost due to fact that trial results suggested schools were well-stocked with equipment. Effect size based on the results of the sensitivity analysis conducted within-trial (imputation of missing data), 254,923 students from 487 NSW schools.
	Brief summary of results (incl. sensitivity results)	The intervention cost AUD $329,952 over 24 months, or AUD$394 per student in the intervention group. This resulted in a cost effectiveness ratio of AUD$56 ($35–$147) per additional minute of MVPA, AUD$1 ($0.6–$2.7) per MET hour gained per person per day, AUD$1408 ($788–$6,570) per BMI unit avoided, and AUD$563 ($282–$3,942) per 10 % reduction in BMI z-score.

		Sensitivity analyses extending the intervention benefit outside the target grade resulted in ICERs of $60 ($37, $150) and $28 ($15, $154) respectively.
	Limitations	Calculation of DALYs out of scope; sensitivity analyses are hypothetical; sustainability of effects unknown
	Funding source	New South Wales Ministry of Health - Health Promotion Demonstration grant
	Conflicts of interest	None declared
**Te Velde et al.(50)**		
48	Study aim	To assess the cost-effectiveness of a nation wide implementation of two school-based interventions promoting fruit and vegetable intake among primary schoolchildren in the Netherlands compared to 'no intervention' and to each other.
	Country	Netherlands
	Currency unit and year	EUR 2003
	Study design	Modelled CUA
	Setting	Schools, home
	Intervention	Pro Children intervention consisting of three main components that targeted children in the last grades of primary education (10–12 years olds). The school component consisted of providing a piece of fruit, a carrot or a tomato for free twice a week, the classroom curriculum consisted of worksheets and a web-based computer tailored feedback tool. The family component encouraged parents to be involved in the project by means of their children's homework assignments, parental newsletters and a parent version of the web-based computer tailored tool.<br><br>The Schoolgruiten intervention was comparable to the Pro Children intervention in that the main strategy to improve fruit and vegetable intake was by means of better availability and accessibility of fruit and vegetables at school through a free fruit and vegetables scheme.

		Additionally, the schools were encouraged, but not obliged, to use a curriculum to increase knowledge and skills related to fruit and vegetables consumption.
	Comparator	No intervention
	Target population	Primary school children, all 10 years olds in the Netherlands
	Perspective	Health care payer
	Time horizon	Lifetime
	Model specification (if applicable)	Proportional multi-state life table
	Measurement and valuation of outcomes	Effect estimates from RCT on fruit and vegetable intake-DALYs<br>Estimate of 30% lifelong effect was based on moderate tracking of F&V consumption from adolescence to young adulthood, but because this was from an observational study the estimate remains essentially arbitrary.
	Measurement and valuation of resources and cost	Costs for the development and implementation of the Pro Children Study were reported in special forms and divided in two categories: (1) curriculum and materials and (2) the fruit and vegetables scheme. Costs for the development and implementation of the Schoolgruiten project were retrieved from AGF-promotion, the organisation responsible for the implementation of the project. To estimate the costs for a nation wide implementation among all 10 years old in the Netherlands, the costs per participating child were multiplied by the total number of 10 years old in the Netherlands as obtained from Statistics Netherlands.<br>Healthcare cost-savings from cases of disease averted.
	Discount rate	3%
	Methods for uncertainty	A bootstrap was performed (10,000 iterations) to estimate the uncertainty around the point estimates
	Methods for sensitivity	In one-way sensitivity analyses, the proportion of the intervention effect that lasts lifelong, the costs, the discounting rates for both costs and health effects and for health effects alone, and finally the value of one DALY were varied.
	Brief summary of results (incl. sensitivity results)	The ICER for the Pro Children intervention in comparison with no intervention was estimated at 5728 perDALY and the ICER for the

		Schoolgruiten intervention in comparison with no intervention at 10,674 per DALY.<br><br>The Pro Children had a 70% chance of being dominant over the Schoolgruiten intervention in terms of cost-effectiveness.<br><br>In the one-way sensitivity analyses the estimated ICERs for the Pro Children intervention in comparison with no intervention varied between cost-saving and €27,000/DALY and the NMBs varied between €−1.9 million and €103.0 million<br><br>When comparing the Pro Children intervention with the Schoolgruiten intervention, all estimates were in favor of the Pro Children intervention, also in the probabilistic sensitivity analyses.
	Limitations	The uncertainty regarding the long-term changes in the consumption of fruits and vegetables.
	Funding source	Fifth Framework Program of the European Commission, Netherlands Organization for Health Research and Development (ZonMW), The Dutch Ministry for Health, Welfare and Sport and Holland Produce Promotion in Zoetermeer, NHMRC
	Conflicts of interest	None declared
**Tran et al.(51)**		
49	Study aim	To assess the cost-effectiveness of the R&C community-wide early childhood obesity prevention intervention if delivered across Australia.
	Country	Australia
	Currency unit and year	AUD 2018
	Study design	Modelled CEA and CUA
	Setting	Community, ECEC
	Intervention	Romp&Chomp, a community-wide obesity prevention intervention emphasising community capacity building and sustainable changes in policy, sociocultural and physical environments in ECEC settings.

	Comparator	No intervention
	Target population	Assumed that the R&C intervention was scaled up and delivered nationally to all Australian children aged from 0 to 5 years (n = 1 906 075)
	Perspective	Funder
	Time horizon	10 years, to age 15 years of the child
	Model specification (if applicable)	Early Prevention of Obesity in Childhood micro-simulation model
	Measurement and valuation of outcomes	BMI (measured). Intervention effect was estimated using a repeat cross-sectional quasi-experimental design to measure the differences in outcomes between the population exposed to the R&C intervention (the intervention sample) compared to the comparison population drawn from other LGAs across Victoria.
	Measurement and valuation of resources and cost	Retrospective costing of intervention costs, using trial records. All assumptions on how the intervention would be implemented at scale were based on the existing literature on community-wide obesity prevention interventions, the management structure reporting in trial records and in consultation with members of the R&C team.<br>Time costs, travel costs, equipment costs; healthcare costs modelled using estimates from the literature adjusted by weight status.
	Discount rate	5%
	Methods for uncertainty	Bootstrapping
	Methods for sensitivity	Univariate and multivariate sensitivity analyses. In sensitivity analysis 1, intervention costs were varied, assuming that they were borne only by the population in which the intervention effect was modelled (i.e., children aged 4–5 years, n = 642 178). In sensitivity analysis 2, higher intervention costs were assumed, based on: (i) intervention resources for participants being paper-based; (ii) the Health Promotion Officer at 562 LGAs being employed at 1 FTE; (iii) the allocation of 2 h each year for early childhood carers and educators to attend training and perform the sweet drink demonstration; (iv) the allocation of 1.5 h each year for dentists to engage with parents and staff training and (v) a longer

		distance of 31.2 km was travelled by Health Promotion Officers within LGAs to deliver training and to attend festivals.41 In sensitivity analysis 3, a "worst case" scenario was also examined, using the low confidence interval (CI) of the intervention effect on BMI (i.e., 0.01 kg/m^2) and the higher intervention cost. In sensitivity analysis 4, the discount rate was reduced from 5% to 3%.
	Brief summary of results (incl. sensitivity results)	Total estimated intervention cost and annual cost per participant were AUD178 million and AUD93, respectively, if implemented nationally. The ICERs were AUD1 126 per BMI unit avoided and AUD26 399 per QALY gained (64% probability of being cost-effective measured against a AUD50 000 per QALY threshold).<br><br>The intervention was not cost-effective when intervention costs were borne only by children aged 4 to 5 years (sensitivity analysis 1), as a result of the higher intervention cost per participant (AUD276; approximately three times higher than the base case cost). The intervention was also not cost-effective under worst case assumptions (sensitivity analysis 3), as a result of the intervention effect being six times lower and the mean intervention cost per participant more than five times higher than in the base case analysis.
	Limitations	Assumptions required to extrapolate costs and effects nationally. No analyses examining potential decay of intervention effect. Probabilistic uncertainty analysis of the range and distribution of intervention effect size and intervention cost per participant was not conducted; instead multiple one-way sensitivity analyses were carried out to test the impact of changes in input assumptions pertaining to costs and effects. Finally, the long-term health burden associated with high BMI was not estimated.
	Funding source	Alfred Deakin Postdoctoral Research Fellowship; National Health and Medical Research Council
	Conflicts of interest	None declared

Vieira et al.(52)		
50	Study aim	To conduct a cost-consequence analysis to evaluate the costs and the health benefits of the ‘Planning Health in School’ programme (PHS-pro).
	Country	Portugal
	Currency unit and year	EUR 2012
	Study design	Pre/post study CCA
	Setting	Elementary schools
	Intervention	Eight learning modules of 45 min (monthly over the academic year), to encourage children to make healthy choices in their daily life.
	Comparator	Non-randomised control group;
	Target population	Elementary school students, Northern region of Portugal, intervention delivered to 449 children aged 10–14 years old. Costing at scale population: 170 elementary schools and 42,953 children in 1,652 classes
	Perspective	Societal
	Time horizon	1 year (pre/post)
	Model specification (if applicable)	NA
	Measurement and valuation of outcomes	Primary outcomes: measured height, weight, waist circumference, body mass index and waist-height ratio collected before and after the intervention to compare the two groups. Eating behaviours were used as secondary outcomes to evaluate change from baseline to follow-up and were self-reported by children using food frequency questionnaires.
	Measurement and valuation of resources and cost	Costed Phase 1 (designing and developing the intervention) and Phase 2 (implementation of the intervention). Human and material resources associated with intervention. Time costs, transportation costs, equipment for assessments and printing documents.<br><br>Per capita intervention costs estimated, where total intervention costs were divided by the number of children who received the intervention. No additional time for extra school lessons was required as the intervention was designed to be included in the science curriculum.
	Discount rate	NA

	Methods for uncertainty	Not undertaken
	Methods for sensitivity	Not undertaken
	Brief summary of results (incl. sensitivity results)	Intervention total costs were an estimated EUR7915.53 per year with a cost of EUR36.14 per year per child attending the program.<br><br>A scale-up costing projection for implementing the intervention to a larger population was estimated to be even lower: EUR18.18 per year per child.
	Limitations	Staff training costs not considered.<br>Based on the field experience acquired during implementation, that authors recommended that the intervention be delivered in each school by at least two health educators, a nutritionist and a trainee assistant.
	Funding source	Foundation for Science and Technology (SFRH/BD/ 79512/2011) and CIEC (UID/CED/00317/2013)
	Conflicts of interest	Not stated
**Wright et al.(53)**		
51	Study aim	To quantify the health and economic impacts of a multi-component regulatory obesity policy intervention in licensed US child care facilities.
	Country	United States
	Currency unit and year	USD 2015
	Study design	Modelled CEA
	Setting	ECEC policy
	Intervention	A hypothetical state-level regulatory policy intervention was developed, which was based on current recommendations regarding healthy behavior practices in child care programs and current state and local child care initiatives. The three components of the interventions were:1) The beverage component: Water made freely available throughout the program day, SSBs replaced with water, 100% juice limited to 6 ounces per child per day, Whole milk be replaced with reduced-fat milk. 2) The physical activity component: Programs to provide opportunities for at least 90 minutes of MVPA over the course of the program day for

		children in full-time care. 3) The screen time component: TV and computer time be educational in nature and limited to 30 minutes per week.
	Comparator	No intervention
	Target population	US preschool-aged children, cohort of up to 6.5 million preschool-aged children attending child care facilities
	Perspective	Societal perspective
	Time horizon	10 years
	Model specification (if applicable)	Childhood Obesity Intervention Cost-Effectiveness Study (CHOICES) model, a Markov-based cohort simulation model
	Measurement and valuation of outcomes	Data from an RCT were used to estimate the change in body weight (kg) resulting from eliminating SSB consumption during child care.<br>For those facilities that would benefit from the policy change, the daily increase in children’s MVPA was calculated by multiplying extra scheduled PA time (30 minutes) by an estimate of adherence to scheduled PA time (94%) and estimates from the published literature of the percentage of PA time provided that children spend in MVPA. The net change in BMI associated with the estimated daily increase in MVPA was calculated using results from a randomized trial that related a change in MVPA during the school day to a change in BMI.<br>The effects of the screen time component of the policy intervention were modeled for the ECEC with no regulatory limits on screen time and for facilities that do not restrict screen time to educational content only. Baseline screen time viewing estimates were obtained from two studies on screen time in child care facilities. The impact of screen time viewing on BMI was calculated using estimates from a randomized trial that related a change in the number of hours of TV watched per day to a change in BMI.
	Measurement and valuation of resources and cost	Policy implementation costs.<br>Three categories of intervention costs were estimated: licensing, training, and beverage costs.

		It was assumed that new regulations would increase costs in existing child care regulatory agencies due to increased inspection time and administrative time for licensing supervisors.<br>The cost of training facility administrators on policy adherence was accounted for, assuming administrators would receive an additional 90–120 minutes of professional development training biannually.<br>Cost savings to facilities from changes in beverages served was accounted for.<br>Therefore, the children in the model who would be in the intervention for less than the full 2 years (e.g., those aged 4 and 5 years who start kindergarten in Year 2 of the hypothetical intervention) were estimated to receive only half the modelled BMI benefits and incur half the costs of the 2-year intervention.
	Discount rate	3%
	Methods for uncertainty	Monte Carlo simulation
	Methods for sensitivity	Alternative baseline behaviour estimates and levels of policy adherence. Additionally, scenario analyses were conducted using alternative policy adherence estimates and outcomes.
	Brief summary of results (incl. sensitivity results)	National implementation could reach 3.69 million children out of the 6.5 million eligible population to watch less TV, get more minutes of moderate and vigorous physical activity, and consume fewer sugar-sweetened beverages.<br><br>The cost was $4.82 million in the first year which would result in 0.0186 fewer BMI units (95% UI1⁄40.00592 kg/m2, 0.0434 kg/m2) per eligible child at a cost of $57.80 per BMI unit avoided.<br>These effects would result in net healthcare cost savings of $51.6 (95% UI$14.2, $134) million over 10 years and 94.7% likely to be cost saving by 2025.<br><br>Sensitivity analyses that assumed higher levels of policy adherence reduced total costs (–$5.92 million in sensitivity analyses vs $4.82

		million in the base case). The sensitivity analysis that considered lower baseline estimates of whole milk and SSB consumption resulted in higher total intervention costs ($8.08 million vs $4.82 million), but did not result in significant differences in effectiveness measures.
	Limitations	The total impact of the intervention was estimated by summing the independent effects of the beverage, physical activity, and screen time components of the intervention, when each component may not be independent and could overestimate the total impact of policy changes on health.<br>Model inputs came from a variety of sources. Some effectiveness estimates were from small, non- representative studies or were based on randomized trials among children slightly older than the preschool-aged child target population.<br>Base case beverage consumption estimates for the model were from an observational study in one state.<br>Baseline estimates for PA time offered were from a self- report survey and there is no consensus as to whether increased outdoor playtime consistently leads to increased MVPA.
	Funding source	Robert Wood Johnson Foundation, Donald and Sue Pritzker Nutrition and Fitness Initiative, JPB Foundation, CDC, including the Nutrition and Obesity Policy Research and Evaluation Network.
	Conflicts of interest	Not stated

Table notes: [#] Summary focus on economic evaluations that are not referenced as primary studies and already included. AASC= Active After-school Communities; ACE= Assessing Cost-Effectiveness; AUD= Australian dollars; BAEW= Be Active Eat Well; BMI= body mass index; BMIZ= body mass index z-score; CBA= cost-benefit analysis; CBI= community-based obesity prevention interventions; CDC= Centers for Disease Control and Prevention; CDM= RIVM Chronic Diseases Model; CDP= chronic disease prevention; CEA= cost-effectiveness analysis; CHAT= Communicating Healthy Beginnings Advice by Telephone; CHC= child health care; CHOICES= Childhood Obesity Intervention Cost Effectiveness Study; CI= confidence interval; CUA= cost-utility analysis; CRE= Centre for Research Excellence; CVD= cardiovascular disease; DALY= disability-adjusted life year; ECE = early childhood education; ECEC: early childhood education and care; EDNP= energy dense nutrient poor; EUR= Euros; FTE= full time equivalent; F&V= fruit and vegetables; GBP= British pounds; GP= general practitioner; HALY= health-adjusted life year; HFSS= high in fat, sugar and salt; HSR=Health Star Rating; HRQoL= Health-related quality of life; ICER=

incremental cost-effectiveness ratio; IRSD= Index of Relative Socio-economic Disadvantage; kg= kilogram; km= kilometre; LGA= local government area; LYS= life years saved; M= million; m= metre; min= minutes; MET= metabolic equivalent task; MVPA= moderate to vigorous physical activity; NA= not applicable; NCD= non-communicable disease; NGO= non-government organisation;NMB= net monetary benefits; NSLP= National School Lunch Program; NSW= New South Wales; NZ= New Zealand; OECD= Organisation for Economic Co-operation and Development; MOVE= Measurement of the Value of Exercise; PA=physical activity; PE=physical education; QALY= quality adjusted life year; RCT= randomised controlled trial; ROI= return on investment; R&C= Romp & Chomp; SBP= systolic blood pressure; SBP=systolic blood pressure; SEIFA= Socio-Economic Indexes for Areas; SEP= socioeconomic position; SMS= short message service; SNAP= Supplemental Nutrition Assistance Program; SPHeP-NCD= Strategic Public Health Planning for NCDs; SSB=sugar-sweetened beverage; TV= television; UI= uncertainty interval; UK= United Kingdom; US: United States; USA= United States of America; USD= United States dollars; VAT= value-added tax; VPA = vigorous physical activity; WHO= World Health Organization; WIC= women, infants and children; WSB= Walking School Bus.

REFERENCES

1. Tricco AC, Lillie E, Zarin W, O'Brien KK, Colquhoun H, Levac D, et al. PRISMA extension for scoping reviews (PRISMA-ScR): checklist and explanation. Annals of internal medicine. 2018;169(7):467-73.
2. Mhairi C, Joanne EM, Amanda S, Srinivasa Vittal K, Sue EB, Simon E, et al. Synthesis without meta-analysis (SWiM) in systematic reviews: reporting guideline. BMJ. 2020;368:l6890.
3. An R, Xue H, Wang L, Wang Y. Projecting the impact of a nationwide school plain water access intervention on childhood obesity: a cost-benefit analysis. Pediatr Obes. 2018;13(11):715-23.
4. Ananthapavan J, Nguyen PK, Bowe SJ, Sacks G, Mantilla Herrera AM, Swinburn B, et al. Cost-effectiveness of community-based childhood obesity prevention interventions in Australia. International Journal of Obesity. 2019;43(5):1102-12.
5. Ananthapavan J, Sacks G, Brown V, Moodie M, Nguyen P, Veerman L, et al. Priority-setting for obesity prevention-The Assessing Cost-Effectiveness of obesity prevention policies in Australia (ACE-Obesity Policy) study. PLoS One. 2020;15(6):e0234804.
6. Robinson E, Nguyen P, Jiang H, Livingston M, Ananthapavan J, Lal A, Sacks G. Increasing the Price of Alcohol as an Obesity Prevention Measure: The Potential Cost-Effectiveness of Introducing a Uniform Volumetric Tax and a Minimum Floor Price on Alcohol in Australia. Nutrients [Internet]. 2020; 12(3).
7. Brown V, Moodie M, Cobiac L, Mantilla Herrera AM, Carter R. Obesity-related health impacts of fuel excise taxation- an evidence review and cost-effectiveness study. BMC Public Health. 2017;17(1):359.
8. Gao L, Flego A, Dunstan DW, Winkler EAH, Healy GN, Eakin EG, et al. Economic evaluation of a randomized controlled trial of an intervention to reduce office workers' sitting time: the "Stand Up Victoria" trial. Scandinavian Journal of Work, Environment & Health. 2018(5):503-11.
9. Huse O, Ananthapavan J, Sacks G, Cameron AJ, Zorbas C, Peeters A, et al. The potential cost-effectiveness of mandatory restrictions on price promotions for sugar-sweetened beverages in Australia. International Journal of Obesity. 2020;44(5):1011-20.
10. Brown V, Ananthapavan J, Veerman L, Sacks G, Lal A, Peeters A, et al. The Potential Cost-Effectiveness and Equity Impacts of Restricting Television Advertising of Unhealthy Food and Beverages to Australian Children. Nutrients. 2018;10(5).
11. Crino M, Herrera AMM, Ananthapavan J, Wu JHY, Neal B, Lee YY, et al. Modelled Cost-Effectiveness of a Package Size Cap and a Kilojoule Reduction Intervention to Reduce Energy Intake from Sugar-Sweetened Beverages in Australia. Nutrients. 2017;9(9).
12. Lal A, Mantilla-Herrera AM, Veerman L, Backholer K, Sacks G, Moodie M, et al. Modelled health benefits of a sugar-sweetened beverage tax across different socioeconomic groups in Australia: A cost-effectiveness and equity analysis. PLOS Medicine. 2017;14(6):e1002326.
13. Mantilla Herrera AM, Crino M, Erskine HE, Sacks G, Ananthapavan J, Mhurchu CN, Lee YY. Cost-Effectiveness of Product Reformulation in Response to the Health Star Rating Food Labelling System in Australia. Nutrients [Internet]. 2018; 10(5).
14. Babey SH, Wu S, Cohen D. How can schools help youth increase physical activity? An economic analysis comparing school-based programs. Preventive Medicine. 2014;69:S55-S60.
15. Barrett JL, Gortmaker SL, Long MW, Ward ZJ, Resch SC, Moodie ML, et al. Cost Effectiveness of an Elementary School Active Physical Education Policy. American Journal of Preventive Medicine. 2015;49(1):148-59.

16. Basto-Abreu A, Barrientos-Gutiérrez T, Vidaña-Pérez D, Colchero MA, Hernández-F M, Hernández-Ávila M, et al. Cost-Effectiveness Of The Sugar-Sweetened Beverage Excise Tax In Mexico. Health Affairs. 2019;38(11):1824-31.
17. Basu S, Seligman H, Bhattacharya J. Nutritional policy changes in the supplemental nutrition assistance program: a microsimulation and cost-effectiveness analysis. Med Decis Making. 2013;33(7):937-48.
18. Bemelmans W, van Baal P, Wendel-Vos W, Schuit J, Feskens E, Ament A, Hoogenveen R. The costs, effects and cost-effectiveness of counteracting overweight on a population level. A scientific base for policy targets for the Dutch national plan for action. Preventive Medicine. 2008;46(2):127-32.
19. Edward B, Alison LM, Yvette DM, Adrian GB, Brianna SF, Nicholas G. The cost-effectiveness of the MobileMums intervention to increase physical activity among mothers with young children: a Markov model informed by a randomised controlled trial. BMJ Open. 2015;5(4):e007226.
20. Cecchini M, Sassi F, Lauer JA, Lee YY, Guajardo-Barron V, Chisholm D. Tackling of unhealthy diets, physical inactivity, and obesity: health effects and cost-effectiveness. Lancet. 2010;376(9754):1775-84.
21. Cobiac LJ, Vos T, Barendregt JJ. Cost-effectiveness of interventions to promote physical activity: a modelling study. PLoS Med. 2009;6(7):e1000110.
22. Cobiac LJ, Vos T, Veerman JL. Cost-effectiveness of interventions to promote fruit and vegetable consumption. PLoS One. 2010;5(11):e14148.
23. Cobiac LJ, Tam K, Veerman L, Blakely T. Taxes and Subsidies for Improving Diet and Population Health in Australia: A Cost-Effectiveness Modelling Study. PLOS Medicine. 2017;14(2):e1002232.
24. Cradock AL, Barrett JL, Kenney EL, Giles CM, Ward ZJ, Long MW, et al. Using cost-effectiveness analysis to prioritize policy and programmatic approaches to physical activity promotion and obesity prevention in childhood. Prev Med. 2017;95 Suppl(Suppl):S17-s27.
25. Dallongeville J, Dauchet L, de Mouzon O, Réquillart V, Soler LG. Increasing fruit and vegetable consumption: a cost-effectiveness analysis of public policies. Eur J Public Health. 2011;21(1):69-73.
26. Döring N, Zethraeus N, Tynelius P, de Munter J, Sonntag D, Rasmussen F. Economic Evaluation of PRIMROSE—A Trial-Based Analysis of an Early Childhood Intervention to Prevent Obesity. Frontiers in Endocrinology. 2018;9.
27. Ekwaru JP, Ohinmaa A, Dabravolskaj J, Maximova K, Veugelers PJ. Cost-effectiveness and return on investment of school-based health promotion programmes for chronic disease prevention. European Journal of Public Health. 2021;31(6):1183-9.
28. Emma JF, Mobeen B, Khine W, Alice S, Anna L, Miranda P, Peymane A. Cost-effectiveness of a community-based physical activity programme for adults (Be Active) in the UK: an economic analysis within a natural experiment. British Journal of Sports Medicine. 2014;48(3):207.
29. Goryakin Y, Aldea A, Lerouge A, Romano Spica V, Nante N, Vuik S, et al. Promoting sport and physical activity in Italy: a cost-effectiveness analysis of seven innovative public health policies. Ann Ig. 2019;31(6):614-25.
30. Graziose MM, Koch PA, Wang YC, Lee Gray H, Contento IR. Cost-effectiveness of a Nutrition Education Curriculum Intervention in Elementary Schools. Journal of Nutrition Education and Behavior. 2017;49(8):684-91.e1.
31. Gulliford MC, Charlton J, Bhattarai N, Charlton C, Rudisill C. Impact and cost-effectiveness of a universal strategy to promote physical activity in primary care: population-based cohort study and Markov model. Eur J Health Econ. 2014;15(4):341-51.

32. Hayes A, Lung T, Wen LM, Baur L, Rissel C, Howard K. Economic evaluation of "healthy beginnings" an early childhood intervention to prevent obesity. Obesity. 2014;22(7):1709-15.
33. Kenney EL, Cradock AL, Long MW, Barrett JL, Giles CM, Ward ZJ, Gortmaker SL. Cost-Effectiveness of Water Promotion Strategies in Schools for Preventing Childhood Obesity and Increasing Water Intake. Obesity (Silver Spring). 2019;27(12):2037-45.
34. Kenney EL, Mozaffarian RS, Long MW, Barrett JL, Cradock AL, Giles CM, et al. Limiting Television to Reduce Childhood Obesity: Cost-Effectiveness of Five Population Strategies. Child Obes. 2021;17(7):442-8.
35. Killedar A, Wen LM, Tan EJ, Marshall S, Taki S, Buchanan L, et al. Economic evaluation of the Communicating Healthy Beginnings Advice by Telephone trial for early childhood obesity prevention. Obesity. 2022;30(11):2256-64.
36. Long MW, Gortmaker SL, Ward ZJ, Resch SC, Moodie ML, Sacks G, et al. Cost Effectiveness of a Sugar-Sweetened Beverage Excise Tax in the U.S. Am J Prev Med. 2015;49(1):112-23.
37. Long MW, Polacsek M, Bruno P, Giles CM, Ward ZJ, Cradock AL, Gortmaker SL. Cost-Effectiveness Analysis and Stakeholder Evaluation of 2 Obesity Prevention Policies in Maine, US. J Nutr Educ Behav. 2019;51(10):1177-87.
38. Magnus A, Moodie ML, Ferguson M, Cobiac LJ, Liberato SC, Brimblecombe J. The economic feasibility of price discounts to improve diet in Australian Aboriginal remote communities. Australian and New Zealand Journal of Public Health. 2016;40(S1):S36-S41.
39. Magnus A, Haby MM, Carter R, Swinburn B. The cost-effectiveness of removing television advertising of high-fat and/or high-sugar food and beverages to Australian children. International Journal of Obesity. 2009;33(10):1094-102.
40. Mizdrak A, Telfer K, Direito A, Cobiac LJ, Blakely T, Cleghorn CL, Wilson N. Health Gain, Cost Impacts, and Cost-Effectiveness of a Mass Media Campaign to Promote Smartphone Apps for Physical Activity: Modeling Study. JMIR Mhealth Uhealth. 2020;8(6):e18014.
41. Moodie M, Haby M, Galvin L, Swinburn B, Carter R. Cost-effectiveness of active transport for primary school children - Walking School Bus program. International Journal of Behavioral Nutrition and Physical Activity. 2009;6(1):63.
42. Moodie ML, Carter RC, Swinburn BA, Haby MM. The Cost-effectiveness of Australia's Active After-school Communities Program. Obesity. 2010;18(8):1585-92.
43. Moodie M, Haby MM, Swinburn B, Carter R. Assessing cost-effectiveness in obesity: active transport program for primary school children--TravelSMART Schools Curriculum program. J Phys Act Health. 2011;8(4):503-15.
44. Moodie ML, Herbert JK, de Silva-Sanigorski AM, Mavoa HM, Keating CL, Carter RC, et al. The cost-effectiveness of a successful community-based obesity prevention program: the be active eat well program. Obesity (Silver Spring). 2013;21(10):2072-80.
45. Over EA, Wendel-Vos GW, van den Berg M, Reenen HH, Tariq L, Hoogenveen RT, van Baal PH. Cost-effectiveness of counseling and pedometer use to increase physical activity in the Netherlands: a modeling study. Cost Eff Resour Alloc. 2012;10(1):13.
46. Roux L, Pratt M, Tengs TO, Yore MM, Yanagawa TL, Van Den Bos J, et al. Cost Effectiveness of Community-Based Physical Activity Interventions. American Journal of Preventive Medicine. 2008;35(6):578-88.
47. Rush E, Obolonkin V, McLennan S, Graham D, Harris JD, Mernagh P, Weston AR. Lifetime cost effectiveness of a through-school nutrition and physical programme: Project Energize. Obesity Research & Clinical Practice. 2014;8(2):e115-e22.

48. Sonneville KR, Long MW, Ward ZJ, Resch SC, Wang YC, Pomeranz JL, et al. BMI and Healthcare Cost Impact of Eliminating Tax Subsidy for Advertising Unhealthy Food to Youth. Am J Prev Med. 2015;49(1):124-34.
49. Sutherland R, Reeves P, Campbell E, Lubans DR, Morgan PJ, Nathan N, et al. Cost effectiveness of a multi-component school-based physical activity intervention targeting adolescents: the 'Physical Activity 4 Everyone' cluster randomized trial. International Journal of Behavioral Nutrition and Physical Activity. 2016;13(1):94.
50. te Velde SJ, Lennert Veerman J, Tak NI, Bosmans JE, Klepp KI, Brug J. Modeling the long term health outcomes and cost-effectiveness of two interventions promoting fruit and vegetable intake among schoolchildren. Econ Hum Biol. 2011;9(1):14-22.
51. Tran HNQ, Killedar A, Tan EJ, Moodie M, Hayes A, Swinburn B, et al. Cost-effectiveness of scaling up a whole-of-community intervention: The Romp & Chomp early childhood obesity prevention intervention. Pediatric Obesity. 2022;17(9):e12915.
52. Vieira M, Carvalho GS. Costs and benefits of a school-based health intervention in Portugal. Health Promotion International. 2019;34(6):1141-8.
53. Wright DR, Kenney EL, Giles CM, Long MW, Ward ZJ, Resch SC, et al. Modeling the Cost Effectiveness of Child Care Policy Changes in the U.S. Am J Prev Med. 2015;49(1):135-47.
